# Synthesis of heteroatom-containing pyrrolidine derivatives based on Ti(O-*i*Pr)_4_ and EtMgBr-catalyzed carbocyclization of allylpropargyl amines with Et_2_Zn†

**DOI:** 10.1039/d0ra02677h

**Published:** 2020-05-07

**Authors:** Rita N. Kadikova, Ilfir R. Ramazanov, Azat M. Gabdullin, Oleg S. Mozgovoj, Usein M. Dzhemilev

**Affiliations:** Institute of Petrochemistry and Catalysis of Russian Academy of Sciences 141 Prospekt Oktyabrya Ufa 450075 Russian Federation kadikritan@gmail.com

## Abstract

The Ti(O-*i*Pr)_4_ and EtMgBr-catalyzed regio and stereoselective carbocyclization of *N*-allyl-substituted 2-alkynylamines with Et_2_Zn, followed by deuterolysis or hydrolysis, affords the corresponding methylenepyrrolidine derivatives in high yields. It was found that Ti–Mg-catalyzed carbocyclization of *N*-allyl-substituted 2-alkynylamines with Et_2_Zn is equally selective in dichloromethane, hexane, toluene, and diethyl ether. The reaction was tolerant to the presence of aryl, alkyl, trimethylsilyl, methoxymethyl and aminomethyl substituents on the alkyne. A selective method was proposed for the preparation of bis-pyrrolidine derivatives using Ti–Mg-catalyzed carbocyclization of bis-allylpropargyl amines with Et_2_Zn.

## Abstract 2

The Ti(O-*i*Pr)_4_ and EtMgBr-catalyzed regio and stereoselective carbocyclization of *N*-allyl-substituted 2-alkynylamines with Et_2_Zn, followed by deuterolysis or hydrolysis, affords the corresponding methylenepyrrolidine derivatives in high yields.

## Graphical abstract

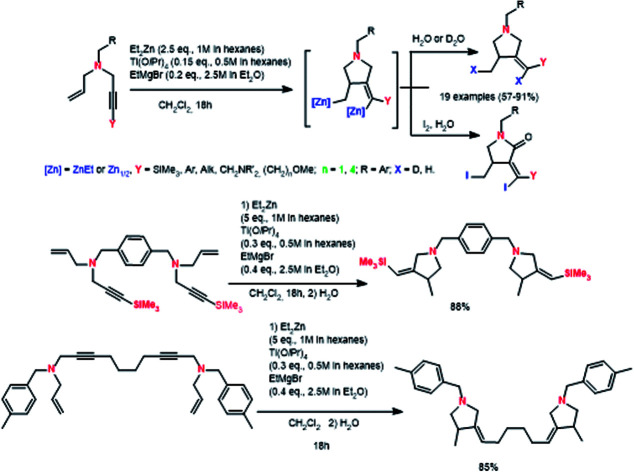

## Introduction

Five- and six-membered nitrogen-containing heterocycles are frequently encountered structural elements of lots of natural and biologically active compounds,^1–4^ such as pyrrolizidine alkaloids^5,6^ and carbapenems.^7,8^ For example, nitrogen heterocycles with an aryl moiety in the β- or γ-position relative to nitrogen are of considerable interest as neuroactive compounds, as they are conformational constrained analogues of neurotransmitters such as serotonin and dopamine and opiate receptor ligands.^9,10^ Of particular interest are 3-benzylpyrrolidine derivatives exhibiting biological activities such as protein kinase C inhibitors,^11,12^ NK-3 receptor antagonists,^13^ and dopamine receptor antagonists.^14^ Thus, development of new efficient synthetic routes to five- and six-membered heterocycles is an important task. Transition metal-catalyzed cyclization of enynes is an efficient tool for the design of carbo and heterocyclic compounds.^15–17^ One of the earliest approach to intramolecular cyclization of enynes and substituted *N*-allylpropargylamines consists in the use of low-valent zirconium complexes generated by treating Cp_2_ZrCl_2_ with Mg and HgCl_2_, or 2 equiv. of alkyllithium, such as *n*-BuLi, or a Grignard reagent, such as EtMgBr.^18,19^ Low-valent iron complexes generated using Et_2_Zn were also active towards carbocyclization of activated enynes.^20^ A known method for the preparation of acyl-substituted pyrrolidines is Me_2_Zn/Ni(0)-promoted cyclization of nitrogen-containing alkynyl enones.^21^ The cyclization of *N*-allyl-*N*-benzyl-3-(trimethylsilyl)-2-propynylamine on treatment with triallylmanganate is not stereoselective and gives a 1 : 1 mixture of Z/*E* isomers in 55% overall yield.^22^ Meanwhile, the reaction of *N*-benzyl-*N*-(3-(trimethylsilyl)prop-2-yn-1-yl)prop-2-en-1-amine with four equivalents of methallylmagnesium chloride in the presence of catalytic amounts of CrCl_3_ is stereoselective, giving the *E*-isomer in 73% yield.^23^ The synthesis of acyl-substituted pyrrolidine derivatives with an exocyclic double bond by treatment of allyl-substituted propargylamines with methylmanganese carbonyl complex was reported.^24^ An unusual Pd(ii)-catalyzed carbocyclization of nitrogen-containing 1,6-enynes accompanied by Pd-initiated migration of β-hydrogen to give 1,3- and 1,4-dienes was described.^25^ Palladium-catalyzed intramolecular cyclization of nitrogen-containing enynyl acetate in the presence of ZnCl_2_ followed by cross-coupling with alkenylstannane results in the selective formation of pyrrolidine derivative.^26^ A pyrrolidine derivative with a conjugated double bond is formed in a similar way upon Pd-catalyzed reaction of nitrogen-containing acetoxyenyne with tris(isopropenyl)indium.^27^ An example involving low-valent titanium complex is the reaction of the Sato reagent, (η^2^-propene)Ti(O-*i*-Pr)_2_ (obtained from Ti(O-*i*-Pr)_4_ and *i*-PrMgCl in 1 : 2 ratio), with *N*-(4-methylbenzyl)-*N*-(prop-2-yn-1-yl)prop-2-en-1-amine with a terminal double bond giving the cyclization product in 53% yield.^28^ Now we demonstrate that the reaction of *N*,*N*-dialkyl-substituted 2-alkynylamines with Et_2_Zn catalyzed by the Ti(*i*-OPr)_4_–EtMgBr system results in the selective formation of 2-zincoethylzincation products.^28,29^ Relying on analysis of the above literature, we assumed that Ti–Mg-catalyzed carbozincation of functionalized allyl-substituted propargylamines could serve for the development of selective one-pot methods for the synthesis of various methylenepyrrolidine derivatives. As a continuation of research on the Ti–Mg-catalyzed carbozincation of functionally substituted alkyne substrates, we studied here carbocyclization of a variety of allyl-substituted propargylamines.

**Scheme 1:**
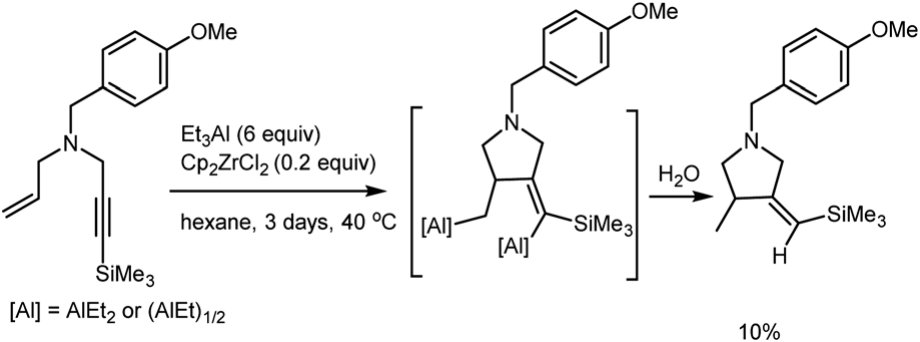
Ti–Mg-catalyzed reaction of *N*-(4-methoxybenzyl)-*N*-(3-(trimethylsilyl)prop-2-yn-1-yl)prop-2-en-1-amine with Et_2_Zn.

Previously, we have showed that Zr-catalyzed carbocyclization of dialkyl-substituted propargylamines results in the selective formation of products of 2-aluminum ethylalumination in high yields.^30^ We were interested in studying the behavior of allyl-substituted propargylamines towards Zr-catalyzed cycloalumination. However, after 24 hours of the reaction of *N*-(4-methoxybenzyl)-*N*-(3-(trimethylsilyl)prop-2-yn-1-yl)prop-2-en-1-amine, prepared from ethynyltrimethylsilane and *N*-(4-methoxybenzyl)prop-2-en-1-amine,^31^ with 3 equivalents of Et_3_Al in the presence of 20 mol% Cp_2_ZrCl_2_ in hexane at 40 °C, the yield of the carbocyclization product did not exceed 5–10% (Scheme 1).

The increase in the amount of Et_3_Al taken in the reaction to 6 equivalents and increase in the reaction time to 3 days did not result in higher yields of the target product. The observed low conversion of the nitrogen-containing substrate may be attributable to steric and electronic factors involved in the coordination of low-valent Cp_2_Zr to a enyne molecule.^30^ It should be borne in mind that coordination of the Et_3_Al aluminum atom to the nitrogen lone pair may not only decrease the triple bond nucleophilicity, but also generate additional steric hindrance for coupling of the olefinic and acetylenic moieties of the enyne substrate with the zirconium atom bearing bulky cyclopentadienyl ligand. The steric hindrance arising at the stage of coupling of the olefinic and acetylenic enyne moieties with the low-valent zirconocene may also be enhanced due to the presence of bulky benzyl substituent at the nitrogen atom.

## Results and discussion

**Scheme 2:**
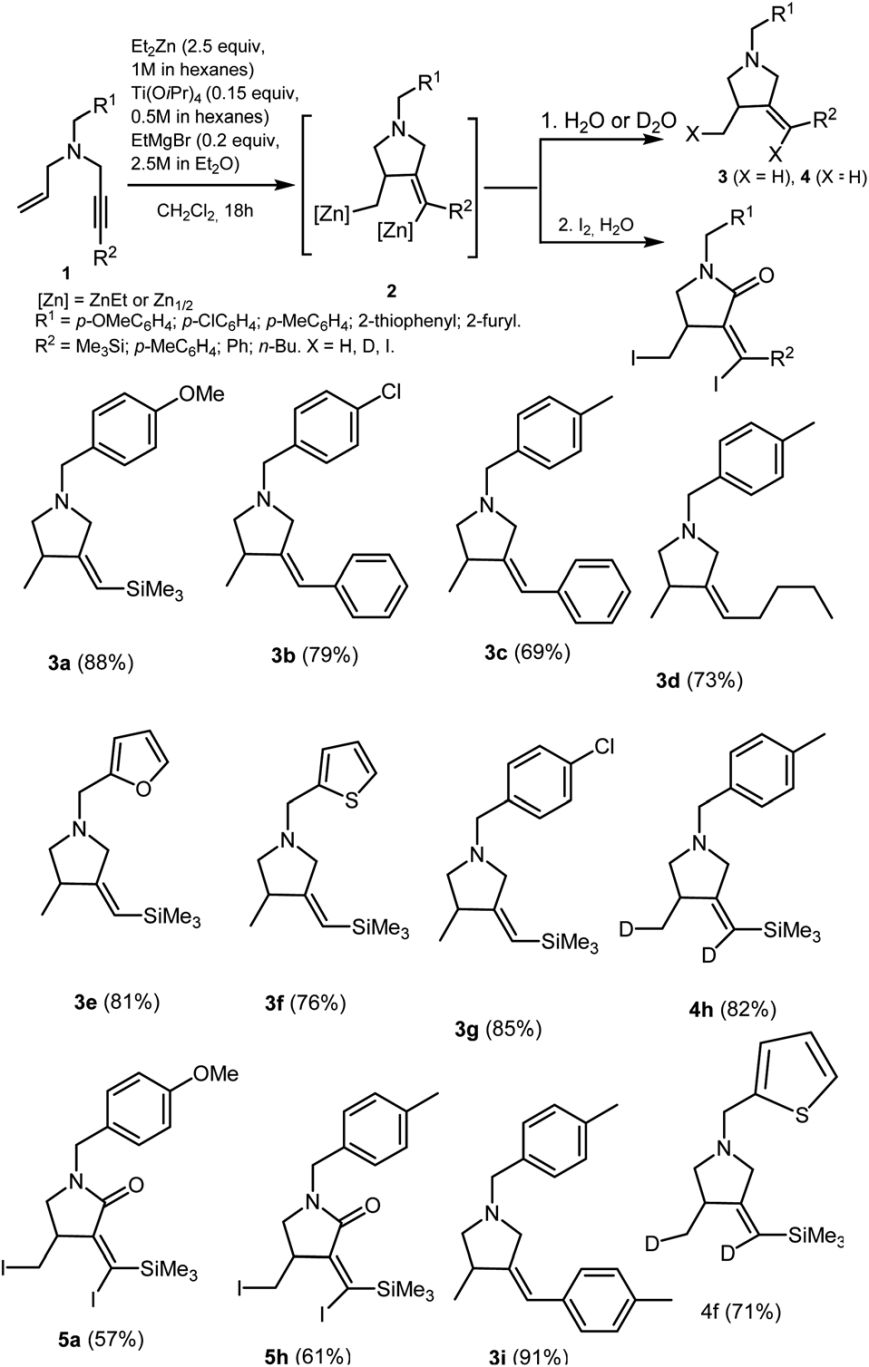
Ti–Mg-catalyzed carbocyclization of allyl substituted propargylamines with Et_2_Zn in CH_2_Cl_2_.

We found that the reaction of *N*-allyl-substituted propargylamines 1 with 2.5 equivalents of Et_2_Zn (1 M in hexane) in the presence of 15 mol% of Ti(O-*i*Pr)_4_ (0.5 M in hexane) and 20 mol% of EtMgBr (2.5 M in Et_2_O) conducted in dichloromethane at room temperature for 18 hours gives, after deuterolysis or hydrolysis, methylenepyrrolidine derivatives 3 and 4 with *Z*-configuration of the double bond (Scheme 2). The structure of the resulting methylenepyrrolidine derivatives was established by 1D and 2D NMR spectroscopy of products of their hydrolysis 3a–g,i and deuterolysis 4h,f (Scheme 2). The *Z*-configuration of the double bond of 3-methyl-4-methylenepyrrolidines 3a–3g, 3i, 4h,f was established by NOESY experiments. For example, the NOESY spectra of 3a and 3b clearly show the cross-peaks between the hydrogen atom at the double bond (*δ* = 5.31 and *δ* = 6.32, respectively) and the hydrogen atoms of the methyl substituent (*δ* = 1.09 and *δ* = 1.30, respectively) of the pyrrolidine ring, that indicates the formation of *Z*-diastereomers in the reaction mixtures. The selective formation of methylenepyrrolidine derivatives 3 and 4 with the *Z*-configuration of the double bond is supported by the mechanism of the Ti–Mg-catalyzed allylpropargyl amines carbometallation reaction using Et_2_Zn (the mechanism is described below in Scheme 7).

Our study demonstrated that the presence of furan (3e), thiophene (3f), *para*-methoxybenzyl (3a), and *para*-chlorobenzyl (3b) substituents at nitrogen atom does not prevent regio and stereoselective carbocyclization of enynes on treatment with Et_2_Zn in the presence of catalytic amounts of Ti(O-*i*Pr)_4_ and EtMgBr.

The carbocyclization of enynes with an aryl (3b, 3c, 3i), trimethylsilyl (3a, 3e, 3f, 3g, 4h, 5a), or butyl (3d) substituent at the triple bond is equally regio and stereoselective. The presence of two deuterium atoms (4h) or two iodine atoms (5a,h) in the methylenepyrrolidine molecules obtained after deuterolysis and iodinolysis attests to the oganometallic nature of intermediate 2. Similarly, Ti–Mg-catalyzed carbocyclization of non-activated and oxygen-containing enynes with Et_2_Zn proceeds.^32^ To our surprise, the reaction of the organozinc intermediate 2 with I_2_ leads to the selective formation of diiodo-substituted pyrrolidin-2-ones – cyclic amides 5a,h. It should be noted that tertiary cyclic amides are widespread structural units of many pharmaceuticals, such as lenalidomide, piperine, evodiamine, diazepam, *etc.*^33–37^ It is known that the alpha-carbon atom of tertiary amines can by easily oxidized to a carbonyl group under the action of such oxidizing agents as iodosobenzene, PhCO_3_^*t*^Bu, ^*t*^BuOOH and RuO_2_/NaIO_4_,^38–42^ as well as under the action of O_2_ in the presence of Ru-, Au-, Fe- and Cu-containing catalysts.^43–47^ However, the mechanism of the formation of the substituted pyrrolidin-2-ones 5a,h in our case will be the subject of our futher study. This issue deserves special consideration, as the obtained transformation is selective method for the preparation of cyclic amides of different structures. Thus, Ti–Mg-catalyzed carbocyclization of nitrogen-containing enynes on treatment with Et_2_Zn has benefits such as tolerance to bulky groups in the unsaturated substrate molecule and the possibility of carbometallation of enynes with a variety of heterofunctional substituents.

It is known that the reaction of Sato reagent (η^2^-propene)Ti(O*i*-Pr)_2_ (obtained from Ti(O*i*-Pr)_4_ and *i*-PrMgCl in a ratio of 1 : 2) with *N*-(4-methylbenzyl)-*N*-(prop-2-yn-1-yl)prop-2-en-1-amine that has a terminal triple bond, gives a cyclization product with 53% yield.^25^ However, our attempts to cyclize the 1,6-enynes described in the article using one equivalent of Ti(O*i*-Pr)_4_ and two equivalents of EtMgBr in the absence of Et_2_Zn led to the non-selective formation of a mixture of unidentified reaction products. The reaction of 1,6-enynes with 2 equivalents of EtMgBr in the presence of catalytic amounts of Ti(O*i*-Pr)_4_ does not occur. Using a stoichiometric amount of Et_2_Zn and catalytic amounts of Ti(O*i*-Pr)_4_ and EtMgBr the reaction gave only one reaction product in high yield. The question of the reasons for this selectivity is complex and we do not have enough data to provide a convincing theory. However coordination effects play a significant role in the reaction under study. So, the conversion of 2-alkynylamines and 1-alkynylphosphines^28^ is excellent, but the reaction with decyne-5 proceeds poorly. Further, the reaction goes well in Et_2_O, CH_2_Cl_2_, hexane, toluene, benzene and anisole but does not proceed in 1,4-dioxane, tetrahydrofuran, 1,2-dichloroethane, 1,2-dimethoxyethane, chloroform, and triethylamine.^29^

**Scheme 3:**
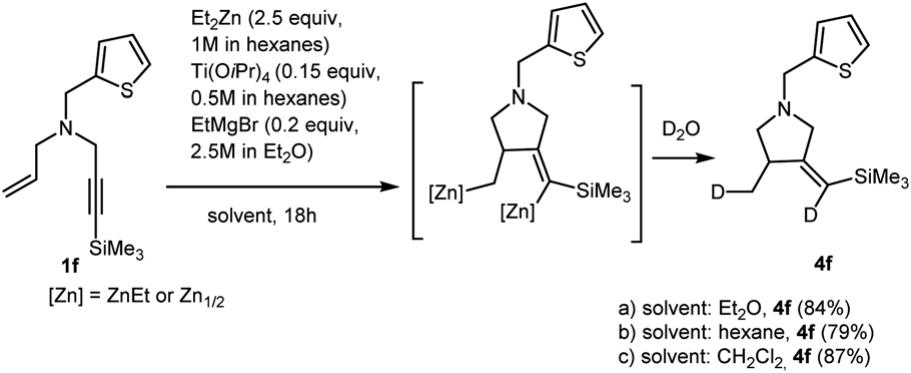
Ti–Mg-catalyzed carbocyclization reactions of *N*-(4-methylbenzyl)-*N*-(3-(trimethylsilyl)prop-2-yn-1-yl)prop-2-en-1-amine with Et_2_Zn in various solvents.

We studied carbocyclization of nitrogen-containing enynes in various solvents. At the same time, it must be taken into account that hexane (Et_2_Zn (1 M in hexane) and Ti(O-*i*Pr)_4_ (0.5 M in hexane)), and Et_2_O (EtMgBr (2.5 M in Et_2_O)) are always present in the reaction mixture. Quite recently, we reported that Ti–Mg-catalyzed 2-zincoethylzincation of substituted 2-alkynylamines with Et_2_Zn proceeds equally selectively in diethyl ether, anisole, dichloromethane, hexane, benzene, and toluene.^29^ In this study, we found that the formation of carbocyclization product 4f upon the reaction of *N*-(thiophen-2-ylmethyl)-*N*-(3-(trimethylsilyl)prop-2-yn-1-yl)prop-2-en-1-amine 1f with 2.5 equivalents of Et_2_Zn in the presence of 15 mol% of Ti(O-*i*Pr)_4_, (0.5 M in hexane) and 20 mol% of EtMgBr (2.5 M in Et_2_O) is regio and stereoselective not only in dichloromethane (as shown in Scheme 2), but also in diethyl ether, hexane, and toluene (Scheme 3).

**Scheme 4:**
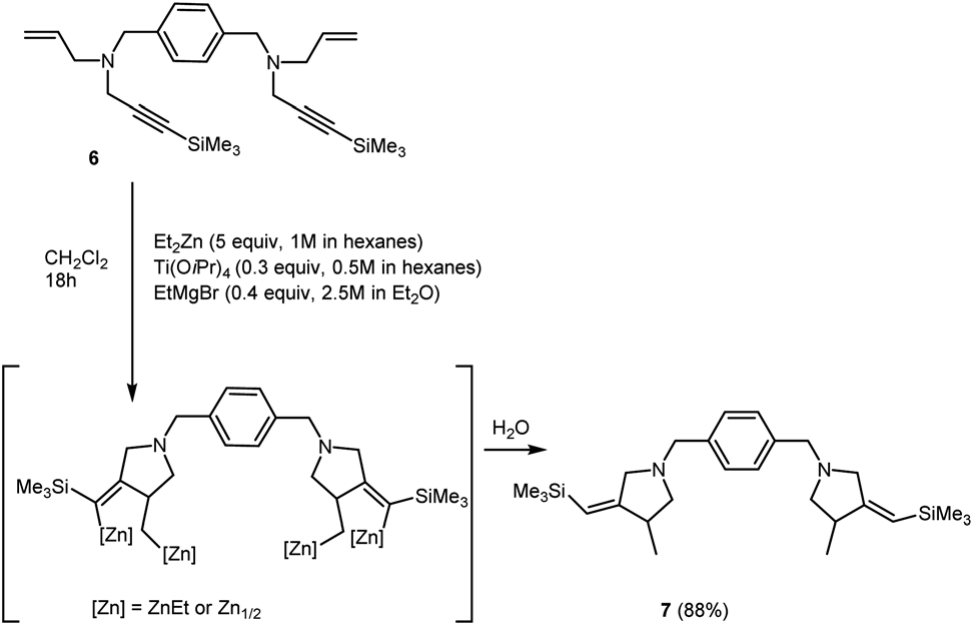
Ti–Mg-catalyzed carbocyclization of *N*,*N*′-(1,4-phenylenebis(methylene))bis(*N*-(3-(trimethylsilyl)prop-2-yn-1-yl)prop-2-en-1-amine) with Et_2_Zn in CH_2_Cl_2_.

We obtained bis-methylenepyrrolidine derivative 7 by the reaction of *N*,*N*′-(1,4-phenylenebis(methylene))bis(*N*-(3-(trimethylsilyl)prop-2-yn-1-yl)prop-2-en-1-amine) 6 with 5 equivalents of Et_2_Zn in the presence of 30 mol% of Ti(O-*i*Pr)_4_, (0.5 M in hexane) and 40 mol% of EtMgBr (2.5 M in Et_2_O) in dichloromethane (Scheme 4).

**Scheme 5:**
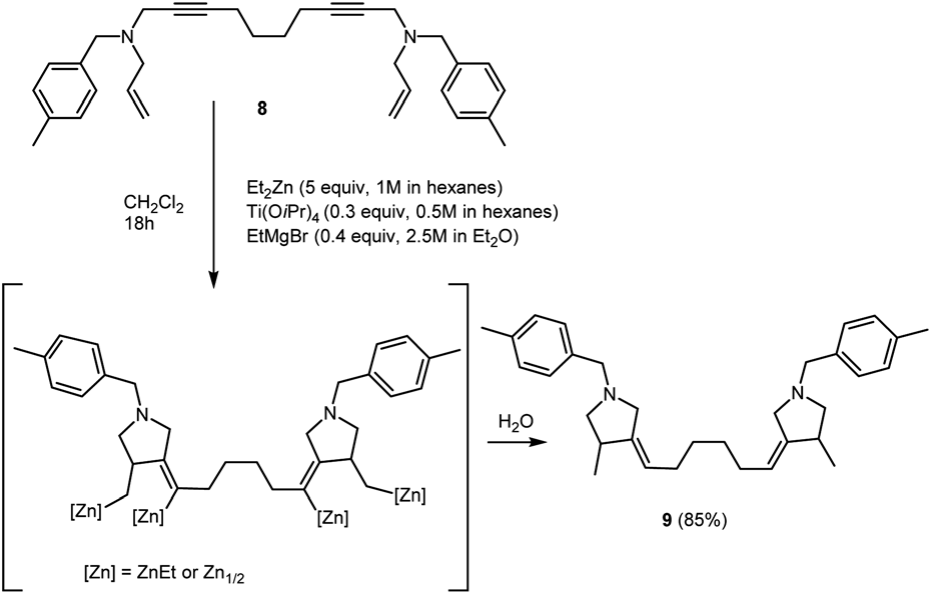
Ti–Mg-catalyzed carbocyclization of *N*^1^,*N*^10^-diallyl-*N*^1^,*N*^10^-bis(4-methylbenzyl)deca-2,8-diyne-1,10-diamine with Et_2_Zn in CH_2_Cl_2_.

We also succeeded to perform the carbocyclization of *N*^1^,*N*^10^-diallyl-*N*^1^,*N*^10^-bis(4-methylbenzyl)deca-2,8-diyne-1,10-diamine 8, prepared from 1,7-octadiyne, to give bis-methylenepyrrolidine derivative 9 (1,6-bis(4-methyl-1-(4-methylbenzyl)pyrrolidin-3-ylidene)hexane) (Scheme 5).

**Scheme 6:**
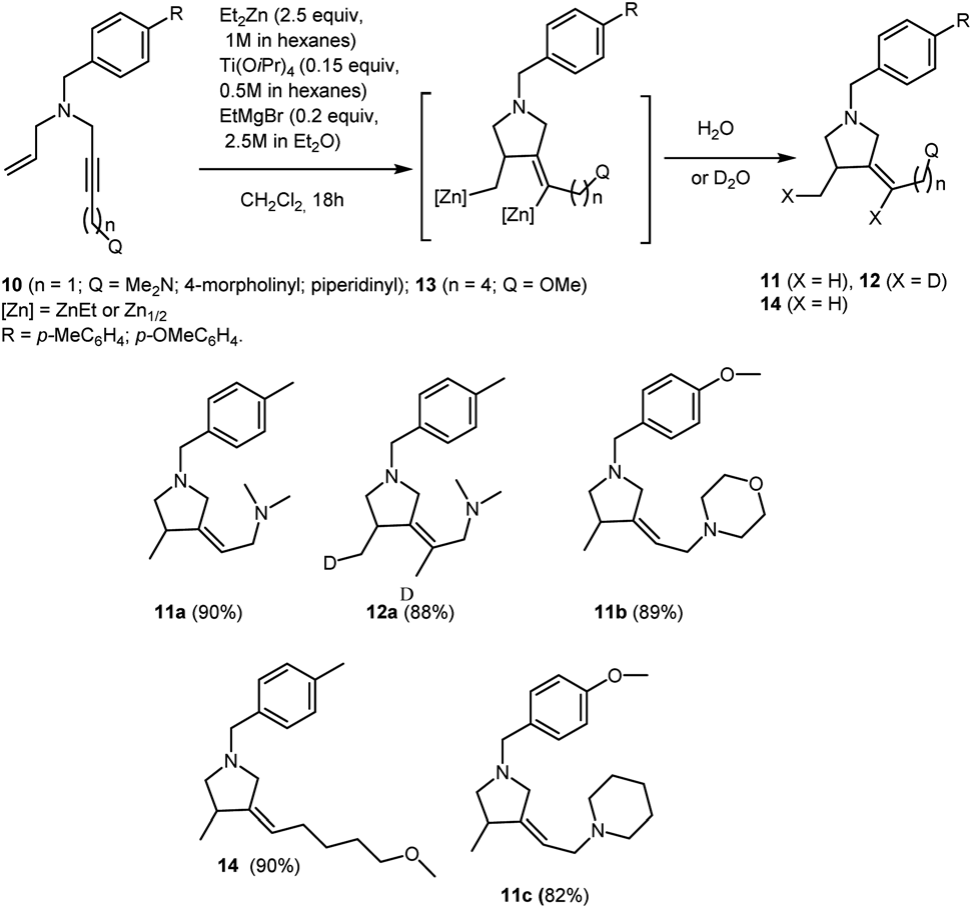
Ti–Mg-catalyzed carbocyclization of allyl substituted but-2-yne-1,4-diamines and *N*-allyl-substituted of oxygen-containing but-2-yn-1-amine with Et_2_Zn in CH_2_Cl_2_.

This study indicates that Ti–Mg-catalyzed carbocyclization of nitrogen-containing enynes with Et_2_Zn is equally efficient both for enynes with alkyl substituent at the triple bond (*e.g.*, 3d and 9) and enynes with aryl and trimethylsilyl substituents at the triple bond (*e.g.*, 3a–c, 3e–g, 3i, 4h,f, 5a,h). For example, it is known that cyclization of unfunctionalized enynes in the course of the Cp_2_ZrCl_2_-catalyzed cycloalumination occurs selectively if directing groups such as phenyl or trimethylsilyl group are present at the triple bond.^48^ Perhaps, the agostic interaction between the *ortho*-hydrogen atom of the phenyl group,^49^ or the trimethylsilyl group, and the zirconium atom may be a favorable factor promoting the cyclization of enynes in the course of cycloalumination. From this standpoint, it was of interest to study the Ti–Mg-catalyzed reaction of Et_2_Zn with nitrogen-containing 1,6-enynes containing additional heterofunctional substituents at the triple bond. Carbocyclization of enynes with a bifunctionally substituted triple bond may serve, in the future, for the development of one-pot syntheses of polyfunctionalized pyrrolidine derivatives. Additionally, study of the behavior of these enyne substrates towards carbocyclization would shed light on the electronic and steric effects of substituents at the triple bond in the reaction. We found that the reaction of *N*-allyl-substituted but-2-yn-1,4-diamines 10 with 2.5 equivalents of Et_2_Zn in the presence of 15 mol% of Ti(O-*i*Pr)_4_, (0.5 M in hexane) and 20 mol% of EtMgBr (2.5 M in Et_2_O) in dichloromethane results in the regio and stereoselective formation of carbocyclization products 11 and 12 in high yields (Scheme 6). Thus, the presence of the second aminomethyl group at the triple bond in the nitrogen-containing enyne molecules 10 does not prevent the intramolecular cyclization.

The reaction of acetylenic ether 13 with 2.5 equivalents of Et_2_Zn in the presence of 15 mol% of Ti(O-*i*Pr)_4_, (0.5 M in hexane) and 20 mol% of EtMgBr (2.5 M in Et_2_O) in dichloromethane results in regio and stereoselective formation of methoxy-substituted pyrrolidine derivative 14 (Scheme 6). It is worth noting that, unlike *N*,*N*-dialkyl substituted 2-alkynylamines, substituted acetylenic alcohols and their ethers are unreactive towards Ti–Mg-catalyzed 2-zincoethylzincation.^28,29^

**Scheme 7:**
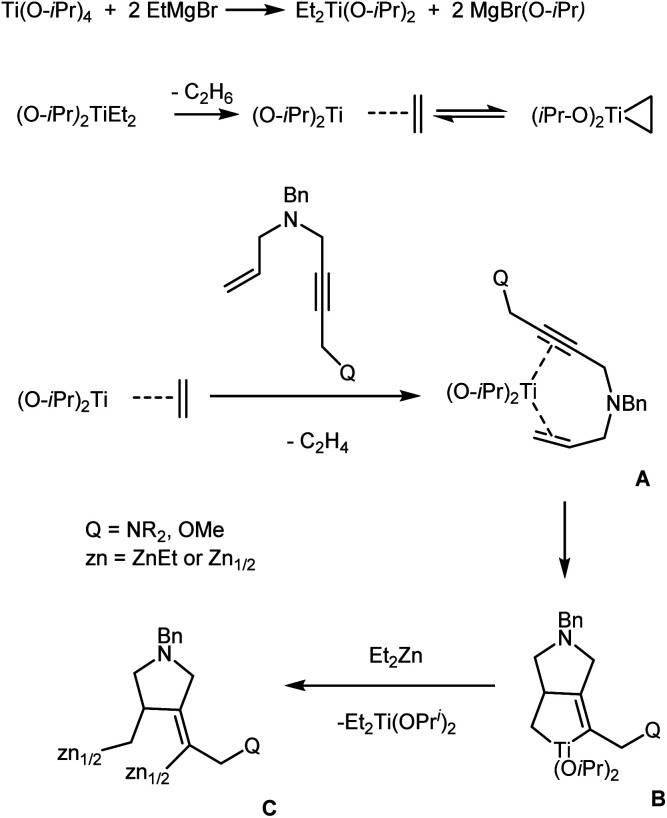
Putative mechanism of Ti–Mg-catalyzed reaction of allyl substituted but-2-yn-1,4-diamines with Et_2_Zn.

According to the reaction scheme that we proposed (Scheme 7), ligand exchange between Ti(O-*i*Pr)_4_ and EtMgBr gives (O-*i*Pr)_2_TiEt_2_, which is converted to titanium(ii)–ethylene complex (titanacyclopropane intermediate). The displacement of ethylene from the titanium coordination sphere by a enyne molecule affords intermediate complex A. The subsequent coupling of the acetylene and ethylene moieties of the enyne molecule gives titanacyclopentene intermediate B, which undergoes transmetallation with Et_2_Zn to be converted to organozinc intermediate C. The deuterolysis (or hydrolysis) of the latter furnishes the pyrrolidine derivative.

## Conclusions

We report a regio and stereoselective Ti(O-*i*Pr)_4_ and EtMgBr-catalyzed carbocyclization of allyl propargyl amines with Et_2_Zn. It was demonstrated that the presence of amine and ether groups at the enyne triple bond does not prevent carbocyclization of allyl-substituted 2-alkynylamines. The study resulted in the development of an efficient method for the synthesis of a variety of heteroatom-containing pyrrolidine derivatives *via* organozinc synthesis. The observed tolerance of the Ti–Mg-catalyzed carbocyclization of enynes to the presence of various heterofunctional substituents opens prospects for further use of organozinc synthesis to develop one-pot syntheses of polyfunctional pyrrolidine derivatives. In connection with the obtained carbocyclization reaction we plan to develop an effective method for preparing methylenepiperidine derivatives based on Ti–Mg-catalyzed carbocyclization of *N*-homoallyl substituted 2-alkynylamines.

## Experimental section

### General information

**Fig. 1:**
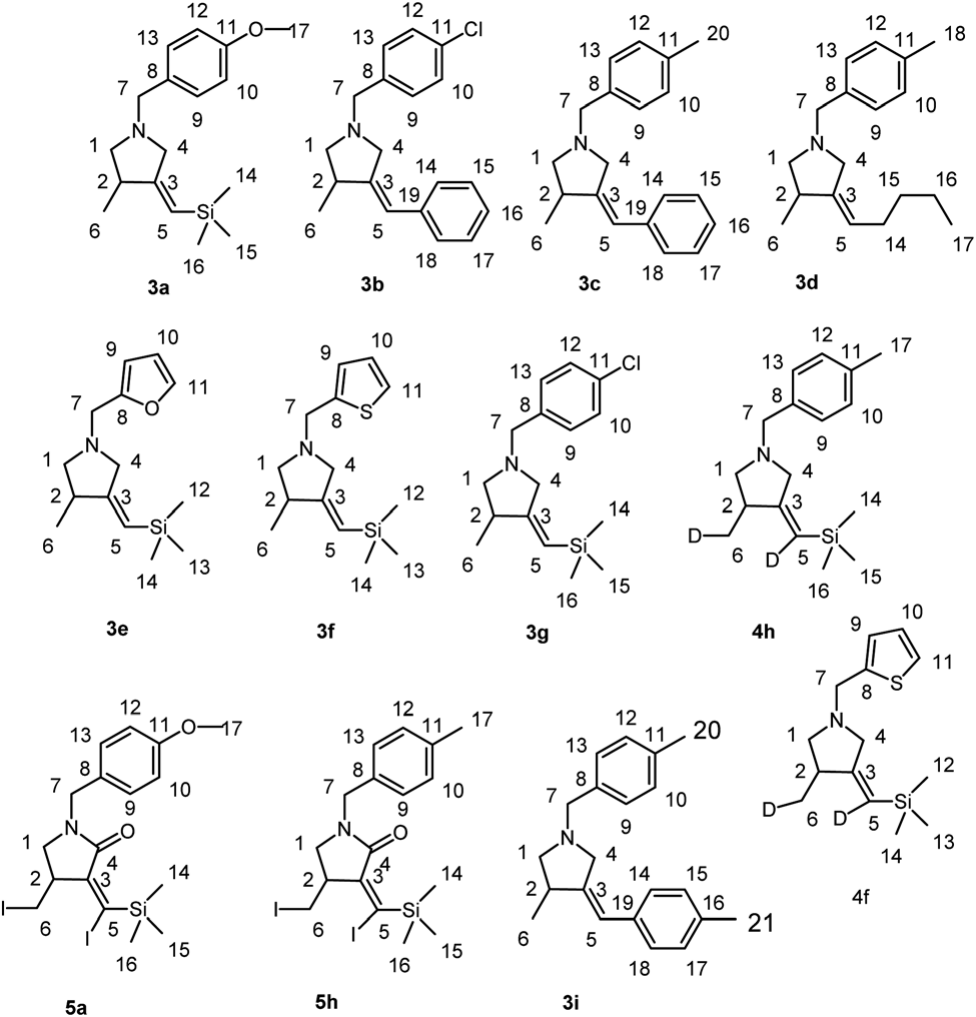
The numbering of atoms in the ^13^C- and ^1^H-NMR spectra of the compounds 3a–g, 3i, 4h,f and 5a,h.

**Fig. 2:**
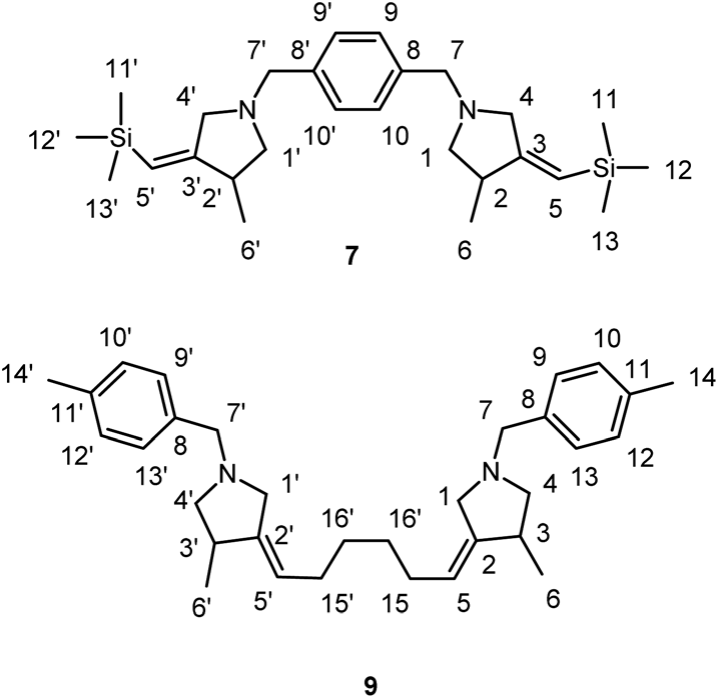
The numbering of atoms in the ^13^C- and ^1^H-NMR spectra of the compounds 7 and 9.

**Fig. 3:**
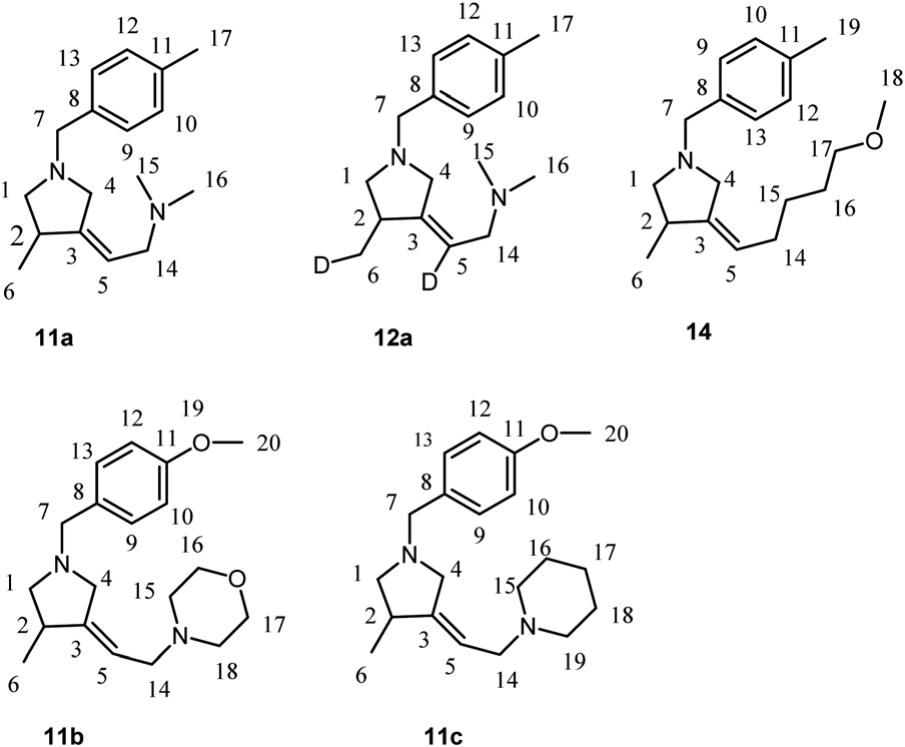
The numbering of atoms in the ^13^C- and ^1^H-NMR spectra of the compounds 11a–c, 12a and 14.

The reagents were obtained from Sigma-Aldrich or Acros. Hexane and dichloromethane were distilled over P_2_O_5_. Diethyl ether, tetrahydrofuran, 1,4-dioxane, toluene, benzene and anisole were dried over sodium. Dried 1,2-dimethoxyethane was obtained from Sigma-Aldrich. 2-Alkynylamines 1a–i and 6, 8 were prepared by aminomethylation of terminal alkynes with aqueous formaldehyde and secondary *N*-aryl-substituted allyl amines under CuBr catalysis.^50^ Nitrogen-containing 1,6-enynes with terminal propargyl and allyl groups were prepared by alkylation of *N*-aryl-substituted allyl amines with propargyl bromide under NaH.^51^ Allyl substituted but-2-yne-1,4-diamines 10 were prepared by aminomethylation of nitrogen-containing 1,6-enynes (with terminal propargyl and allyl groups) by bisamine.^52^ Acetylenic ethers 13 were prepared by aminomethylation of ethers of acetylenic alcohols with aqueous formaldehyde and secondary *N*-aryl-substituted allyl amines under CuBr catalysis.^50^ Nuclear magnetic resonance spectroscopy was performed on a Bruker Avance 500. The ^1^H NMR spectra were recorded at 500 MHz and ^13^C–{^1^H} NMR spectra at 100 MHz in CDCl_3_. The chemical shifts are reported in ppm relative to tetramethylsilane (TMS) as the internal standard. The numbering of atoms in the ^13^C–{^1^H} and ^1^H NMR spectra of the compounds 3a–g, 3i, 4h,f, 5a, 5h, 7, 9, 11a–c, 12a, 14 is shown in Fig. 1–3. Elemental analysis was performed using a Carlo-Erba CHN 1106 elemental analyser. Mass spectra were obtained on a Finnigan 4021 instrument. The yields were calculated from the isolated amount of pyrrolidine and pyrrolidone derivatives obtained from starting nitrogen-containing 1,6-enynes.

### Preparation of 3-methyl-4-methylenepyrrolidines 3a–g, 3i, 4h,f and 5a,h*via* Ti–Mg-catalyzed carbocyclization of *N*-allyl substituted propargylamines with Et_2_Zn in CH_2_Cl_2_

#### (*Z*)-1-(4-Methoxybenzyl)-3-methyl-4-((trimethylsilyl)methylene)pyrrolidine; typical procedure

To a solution of *N*-(4-methoxybenzyl)-*N*-(3-(trimethylsilyl)prop-2-yn-1-yl)prop-2-en-1-amine (574 mg, 2 mmol) and Et_2_Zn (1 M in hexanes, 5 mL, 5 mmol) in CH_2_Cl_2_ (6 mL) was added Ti(O-*i*Pr)_4_ (0.5 M in hexanes, 0.6 mL, 0.3 mmol). Ethylmagnesiurn bromide (2.5 M in Et_2_O, 0.16 mL, 0.4 mmol) was then added and the reaction mixture rapidly turned black. After 18 h at 23 °C, the reaction mixture was diluted with Et_2_O (5 mL), and 25 wt% KOH solution (3 mL) was added dropwise while the reaction flask was cooled in an ice bath. The aqueous layer was extracted with diethyl ether (3 × 5 mL). The combined organic layers were washed with brine (10 mL), dried over anhydrous CaCl_2_. The reaction mixture was filtered through a filter paper and concentrated *in vacuo* to give crude product as a yellow oil. Evaporation of solvent and purification of the residue by column chromatography (diethyl ether : isopropyl alcohol : hexane = 1 : 1 : 8) gave 3a (509 mg, 88%) as colorless oil. *R*_f_ 0.70.

^1^H NMR (500 MHz, CDCl_3_): *δ* = 0.09 (s, 9H, C(14, 15, 16)H_3_), 1.09 (d, *J* = 7 Hz, 3H, C(6)H_3_), 1.99 (t, *J* = 8 Hz, 1H(A), C(1)H_2_), 2.68 (q, *J* = 7 Hz, 1H, C(2)H), 2.98 (t, *J* = 8 Hz, 1H(B), C(1)H_2_), 3.03 (dt, *J* = 14 Hz, *J* = 2 Hz, 2H, C(4)H_2_), 3.56 (d, *J* = 12 Hz, 1H(A), C(7)H_2_), 3.63 (d, *J* = 12 Hz, 1H(B), C(7)H_2_), 3.82 (s, 3H, C(17)H_3_), 5.31 (q, *J* = 2 Hz, 1H, C(5)H), 6.89 (d, *J* = 8 Hz, 2H, C(10, 12)H), 7.28 (d, *J* = 8 Hz, 2H, C(9, 13)H).

^13^C–{^1^H} NMR (500 MHz, CDCl_3_): *δ* = −0.40 (C(14, 15, 16)), 17.34 (C(6)), 40.28 (C(2)), 55.22 (C(17)), 59.32 (C(4)), 60.12 (C(7)), 61.15 (C(1)), 113.63 (C(10, 12)), 116.74 (C(5)), 130.04 (C(9, 13)), 131.66 (C(8)), 158.68 (C(11)), 162.68 (C(3)).

MS (EI): *m*/*z*, % = 289 (1) [M^+^], 287 (11), 214 (11), 166 (8), 121 (100).

Anal. calcd for C_17_H_27_NOSi, (%): C, 70.53; H, 9.40; N, 4.84. Found, %: C, 70.76; H, 9.57; N, 5.07.

#### (*Z*)-3-Benzylidene-1-(4-chlorobenzyl)-4-methylpyrrolidine (3b)

Using the procedure described above *N*-(4-chlorobenzyl)-*N*-(3-phenylprop-2-yn-1-yl)prop-2-en-1-amine (592 mg, 2 mmol) gave crude product that was purified by column chromatography (diethyl ether : isopropyl alcohol : hexane = 1 : 1 : 8) to afford 3b (471 mg, 79%) as colorless oil. *R*_f_ 0.59.

^1^H NMR (500 MHz, CDCl_3_): *δ* = 1.30 (t, *J* = 6 Hz, 3H, C(6)H_3_), 2.19 (m, 1H(A), C(1)H_2_), 2.97 (s, 1H, C(2)H), 3.05 (m, 1H(B), C(1)H_2_), 3.40 (d, *J* = 14 Hz, 1H(A), C(4)H_2_), 3.70 (s, 2H, C(7)H_2_), 3.82 (d, *J* = 14 Hz, 1H(B), C(4)H_2_), 6.32 (s, 1H, C(5)H), 7.25 (m, 1H, C(16)H), 7.26 (m, 2H, C(14, 18)H), 7.36 (m, 2H, C(9, 13)H), 7.38 (m, 2H, C(10, 12)H), 7.40 (m, 2H, C(15, 17)H).

^13^C–{^1^H} NMR (500 MHz, CDCl_3_): *δ* = 18.10 (C(6)), 39.20 (C(2)), 58.43 (C(4)), 59.93 (C(7)), 61.23 (C(1)), 120.69 (C(5)), 126.27 (C(16)), 127.97 (C(14, 18)), 128.45 (C(15, 17)), 128.54 (C(10, 12)), 130.12 (C(9, 13)), 132.79 (C(11)), 137.40 (C(8)), 138.05 (C(19)), 146.98 (C(3)).

Anal. calcd for C_19_H_20_ClN, (%): C, 76.62; H, 6.77; N, 4.70. Found, %: C, 76.45; H, 6.91; N, 4.75.

#### (*Z*)-3-Benzylidene-4-methyl-1-(4-methylbenzyl)pyrrolidine (3c)

Using the procedure described above *N*-(4-methylbenzyl)-*N*-(3-phenylprop-2-yn-1-yl)prop-2-en-1-amine (380 mg, 2 mmol) gave crude product that was purified by column chromatography (diethyl ether : isopropyl alcohol : hexane = 1 : 1 : 8) to afford 3c (382 mg, 69%) as colorless oil. *R*_f_ 0.61.

^1^H NMR (500 MHz, CDCl_3_): *δ* = 1.33 (d, *J* = 7 Hz, 3H, C(6)H_3_), 2.23 (t, *J* = 8 Hz, 1H(A), C(1)H_2_), 2.45 (s, 3H, C(20)H_3_), 3.02 (q, *J* = 7 Hz, 1HC(2)H), 3.11 (t, *J* = 8 Hz, 1H(B), C(1)H_2_), 3.46 (d, *J* = 15 Hz, 1H(A), C(4)H_2_), 3.77 (s, 2H, C(7)H), 3.92 (d, *J* = 15 Hz, 1H(B), C(4)H_2_), 6.34 (s, 1H, C(5)H), 7.25 (d, *J* = 8 Hz, 2H, C(10, 12)H), 7.27 (m, 1H, C(16)H), 7.30 (d, *J* = 8 Hz, 2H, C(14, 18)H), 7.37 (d, *J* = 8 Hz, 2H, C(9, 13)H), 7.42 (d, *J* = 8 Hz, 2H, C(15, 17)H).

^13^C–{^1^H} NMR (500 MHz, CDCl_3_): *δ* = 18.13 (C(6)), 21.23 (C(20)), 39.31 (C(2)), 58.59 (C(4)), 60.46 (C(7)), 61.32 (C(1)), 120.45 (C(5)), 126.14 (C(16)), 128.00 (C(14, 18)), 128.41 (C(15, 17)), 128.75 (C(9, 13)), 129.09 (C(10, 11)), 136.00 (C(8)), 136.58 (C(11)), 138.23 (C(19)), 147.59 (C(3)).

MS (*m*/*z*, %): 277 (41) [M]^+^, 262 (19), 172 (10), 129 (13), 105 (100).

Anal. calcd for C_20_H_23_N, (%): C, 86.59; H, 8.36; N, 5.05. Found, %: C, 86.62; H, 8.43; N, 4.85.

#### (*Z*)-3-Methyl-1-(4-methylbenzyl)-4-pentylidenepyrrolidine (3d)

Using the procedure described above *N*-allyl-*N*-(4-methylbenzyl)hept-2-yn-1-amine (510 mg, 2 mmol) gave crude product that was purified by column chromatography (diethyl ether : isopropyl alcohol : hexane = 1 : 1 : 8) to afford 3d (494 mg, 73%). *R*_f_ 0.68.

^1^H NMR (500 MHz, CDCl_3_): *δ* = 0.91 (m, 3H, C(17)H_3_), 1.08 (d, *J* = 7 Hz, 3H, C(6)H_3_), 1.31 (m, 2H, C(16)H_2_), 1.33 (m, 2H, C(14)H_2_), 1.92 (q, *J* = 7 Hz, 2H, C(15)H_2_), 2.05 (m, 1H(A), C(1)H_2_), 2.37 (s, 3H, C(18)H_3_), 2.69 (q, *J* = 7 Hz, 1H, C(2)H), 2.98 (d, *J* = 14 Hz, 1H(A), C(4)H_2_), 3.01 (t, *J* = 8 Hz, 1H(B), C(1)H_2_), 3.50 (d, *J* = 14H, 1H(B), C(4)H_2_), 3.62 (d, *J* = 13 Hz, 1H(A), C(7)H_2_), 3.66 (d, *J* = 13 Hz, 1H(B), C(7)H_2_), 5.15 (m, 1H, C(5)H), 7.26 (d, *J* = 8 Hz, 2H, C(9, 13)H), 7.16 (d, *J* = 8 Hz, 2H, C(10, 12)H).

^13^C–{^1^H} NMR (500 MHz, CDCl_3_): *δ* = 14.03 (C(17)), 17.59 (C(6)), 21.12 (C(18)), 22.35 (C(16)), 29.14 (C(15)), 31.75 (C(14)), 37.17 (C(2)), 56.59 (C(4)), 60.43 (C(7)), 62.07 (C(1)), 120.05 (C(5)), 136.61 (C(8)), 128.87 (C(9, 13)), 128.96 (C(10, 12)), 143.76 (C(3)).

MS (*m*/*z*, %): 257 (14) [M]^+^, 200 (25), 152 (10), 105 (100).

Anal. calcd for C_18_H_27_N, (%): C, 83.99; H, 10.57; N, 5.44. Found, %: C, 84.28; H, 10.73; N, 5.30.

#### (*Z*)-1-(Furan-2-ylmethyl)-3-methyl-4-((trimethylsilyl)methylene)pyrrolidine (3e)

Using the procedure described above *N*-(furan-2-ylmethyl)-*N*-(3-(trimethylsilyl)prop-2-yn-1-yl)prop-2-en-1-amine (494 mg, 2 mmol) gave crude product that was purified by column chromatography (diethyl ether : isopropyl alcohol : hexane = 1 : 1 : 8) to afford 3e (403 mg, 81%). *R*_f_ 0.74.

^1^H NMR (500 MHz, CDCl_3_): *δ* = 0.09 (s, 9H, C(12, 13, 14)H_3_), 1.09 (d, *J* = 7 Hz, 3H, C(6)H_3_), 2.05 (t, *J* = 9 Hz, 1H(A), C(1)H_2_), 2.70 (q, *J* = 7 Hz, C(2)H), 3.04 (m, 1H(B), C(1)H_2_), 3.06 (m, 1H(A), C(4)H_2_), 3.59 (dd, *J* = 14 Hz, *J* = 2 Hz, 1H(B), C(4)H_2_), 3.65 (d, *J* = 14 Hz, 1H(A), C(7)H_2_), 3.68 (d, *J* = 14 Hz, 1H(B), C(7)H_2_), 5.30 (m, 1H, C(5)H), 6.22 (d, *J* = 3 Hz, 1H, C(9)H), 6.34 (m, 1H, C(10)H), 7.39 (dd, *J* = 2 Hz, *J* = 1 Hz, 1H, C(11)H).

^13^C–{^1^H} NMR (500 MHz, CDCl_3_): *δ* = −0.45 (C(12, 13, 14)), 17.17 (C(6)), 40.25 (C(2)), 52.19 (C(7)), 58.96 (C(4)), 107.86 (C(9)), 110.06 (C(10)), 116.84 (C(5)), 141.97 (C(11)), 152.45 (C(8)), 162.29 (C(3)).

MS (*m*/*z*, %): 249 (16) [M]^+^, 176 (76), 152 (9), 81 (100).

Anal. calcd for C_14_H_23_NOSi, (%): C, 67.42; H, 9.29; N, 5.62. Found, %: C, 67.07; H, 9.14; N, 5.39.

#### (*Z*)-3-Methyl-1-(thiophen-2-ylmethyl)-4-((trimethylsilyl)methylene)pyrrolidine (3f)

Using the procedure described above *N*-(thiophen-2-ylmethyl)-*N*-(3-(trimethylsilyl)prop-2-yn-1-yl)prop-2-en-1-amine (526 mg, 2 mmol) gave crude product that was purified by column chromatography (diethyl ether : isopropyl alcohol : hexane = 1 : 1 : 8) to afford 3f (403 mg, 76%). *R*_f_ 0.80.

^1^H NMR (500 MHz, CDCl_3_): *δ* = 0.09 (s, 9H, C(12, 13, 14)H_3_), 1.10 (d, *J* = 7 Hz, 3H, C(6)H_3_), 2.07 (t, *J* = 8 Hz, 1H(A), C(1)H_2_), 2.70 (m, 1H, C(2)H), 3.04 (t, *J* = 8 Hz, 1H(B), C(1)H_2_), 3.09 (dt, *J* = 14 Hz, *J* = 2 Hz, 1H(A), C(4)H_2_), 3.61 (dd, *J* = 14 Hz, *J* = 2 Hz, 1H(B), C(4)H_2_), 3.84 (d, *J* = 14 Hz, 1H(A), C(7)H_2_), 3.88 (d, *J* = 14 Hz, 1H(B), C(7)H_2_), 5.32 (m, 1H, C(5)H), 6.96 (m, 1H, C(11)H), 6.98 (t, *J* = 3 Hz, 1H, C(10)H), 7.25 (dd, *J* = 5 Hz, *J* = 1 Hz, 1H, C(9)H).

^13^C–{^1^H} NMR (500 MHz, CDCl_3_): *δ* = −0.42 (C(12, 13, 14)), 17.34 (C(6)), 40.35 (C(2)), 54.69 (C(7)), 59.06 (C(4)), 61.04 (C(1)), 116.89 (C(5)), 124.79 (C(9)), 125.50 (C(11)), 126.41 (C(10)), 142.10 (C(8)), 162.44 (C(3)).

MS (*m*/*z*, %): 265 (4) [M]^+^, 192 (31), 97 (100), 73 (20).

Anal. calcd for C_14_H_23_NSSi, (%): C, 63.34; H, 8.73; N, 5.28. Found, %: C, 63.39; H, 8.64; N, 5.11.

#### (*Z*)-1-(4-Chlorobenzyl)-3-methyl-4-((trimethylsilyl)methylene)pyrrolidine (3g)

Using the procedure described above *N*-(4-chlorobenzyl)-*N*-(3-(trimethylsilyl)prop-2-yn-1-yl)prop-2-en-1-amine (584 mg, 2 mmol) gave crude product that was purified by column chromatography (diethyl ether : isopropyl alcohol : hexane = 1 : 1 : 8) to afford 3g (500 mg, 85%). *R*_f_ 0.68.

^1^H NMR (500 MHz, CDCl_3_): *δ* = 0.08 (s, 9H, C(14, 15, 16)H_3_), 1.09 (d, *J* = 7 Hz, 3H, C(6)H_3_), 2.01 (t, *J* = 8 Hz, 1H(A), C(1)H_2_), 2.67 (p, *J* = 7 Hz, 1H, C(2)H), 2.95 (t, *J* = 8 Hz, 1H(B), C(1)H_2_), 3.03 (dt, *J* = 14 Hz, *J* = 2 Hz, 1H(A), C(4)H_2_), 3.52 (dd, *J* = 14 Hz, *J* = 2 Hz, 1H(B), C(4)H_2_), 3.58 (d, *J* = 13 Hz, 1H(A), C(7)H_2_), 3.63 (d, *J* = 13 Hz, 1H(B), C(7)H_2_), 5.32 (m, 1H, C(5)H), 7.30 (d, *J* = 3 Hz, 4H, C(9, 10, 12, 13)H).

^13^C–{^1^H} NMR (500 MHz, CDCl_3_): *δ* = −0.43 (C(14, 15, 16)), 17.45 (C(6)), 40.36 (C(2)), 59.32 (C(4)), 60.01 (C(7)), 61.26 (C(1)), 116.96 (C(5)), 128.39 (C(10, 12)), 130.06 (C(9, 13)), 132.63 (C(11)), 137.46 (C(8)), 162.45 (C(3)).

MS (*m*/*z*, %): 294 (4) [M]^+^, 293 (9), 220 (73), 168 (13), 125 (100), 89 (13), 73 (29).

Anal. calcd for C_16_H_24_ClNSi, (%): C, 65.39; H, 8.23; N, 4.77. Found, %: C, 65.43; H, 8.27; N, 5.01.

#### (*Z*)-3-(Methyl-*d*)-1-(4-methylbenzyl)-4-((trimethylsilyl)methylene-*d*)pyrrolidine (4h)

Using the procedure described above *N*-(4-methylbenzyl)-*N*-(3-(trimethylsilyl)prop-2-yn-1-yl)prop-2-en-1-amine (542 mg, 2 mmol) and D_2_O (instead of H_2_O)gave crude product that was purified by column chromatography (diethyl ether : isopropyl alcohol : hexane = 1 : 1 : 8) to afford 4h (226 mg, 82%). *R*_f_ 0.63.

^1^H NMR (500 MHz, CDCl_3_): *δ* = 0.10 (s, 1H, C(14, 15, 16)H_3_), 1.09 (t, *J* = 8 Hz, 2H, C(6)DH_2_), 2.01 (t, *J* = 8 Hz, 1H(A), C(1)H_2_), 2.38 (s, 3H, C(17)H_3_), 2.68 (p, *J* = 7 Hz, 1H, C(2)H), 2.99 (t, *J* = 8 Hz, 1H(B), C(1)H_2_), 3.06 (d, *J* = 14 Hz, 2H, C(4)H_2_), 3.58 (d, *J* = 13 Hz, 1H(A), C(7)H_2_), 3.67 (d, *J* = 13 Hz, 1H(B), C(7)H_2_), 7.17 (d, *J* = 8 Hz, 2H, C(10, 12)H), 7.26 (d, *J* = 8 Hz, 2H, C(9, 13)H).

^13^C–{^1^H} NMR (500 MHz, CDCl_3_): *δ* = −0.41 (C(14, 15, 16)), 17.07 (t, *J* = 20 Hz, C(6)), 21.14 (C(17)), 40.22 (C(2)), 59.39 (C(4)), 60.49 (C(7)), 61.17 (C(1)), 116.70 (C(5)), 128.82 (C(9, 13)), 128.96 (C(10, 12)), 135.67 (C(8)), 136.53 (C(11)), 162.69 (C(3)).

MS (*m*/*z*, %): 276 (<1) [M]^+^, 275 (<1), 258 (6), 200 (41), 105 (100), 73 (15).

Anal. calcd for C_17_H_25_D_2_NSi, (%): C, 74.11; N, 5.08. Found, %: C, 74.53; N, 5.30.

#### (*Z*)-3-(Methyl-*d*)-1-(thiophen-2-ylmethyl)-4-((trimethylsilyl)methylene-*d*)pyrrolidine (4f)

Using the procedure described above *N*-(thiophen-2-ylmethyl)-*N*-(3-(trimethylsilyl)prop-2-yn-1-yl)prop-2-en-1-amine (526 mg, 2 mmol) and D_2_O gave crude product that was purified by column chromatography (diethyl ether : isopropyl alcohol : hexane = 1 : 1 : 8) to afford 4f (400 mg, 71%). *R*_f_ 0.80.

^1^H NMR (500 MHz, CDCl_3_): *δ* = 0.09 (s, 9H, C(12, 13, 14)H_3_), 1.08 (d, *J* = 7 Hz, 3H, C(6)DH_2_), 2.06 (t, *J* = 8 Hz, 1H(A), C(1)H_2_), 2.69 (m, 1H, C(2)H), 3.04 (t, *J* = 8 Hz, 1H(B), C(1)H_2_), 3.09 (dt, *J* = 14 Hz, *J* = 2 Hz, 1H(A), C(4)H_2_), 3.61 (d, *J* = 14 Hz, 1H(B), C(4)H_2_), 3.84 (d, *J* = 14 Hz, 1H(A), C(7)H_2_), 3.88 (d, *J* = 14 Hz, 1H(B), C(7)H_2_), 6.95 (m, 1H, C(11)H), 6.97 (t, *J* = 3 Hz, 1H, C(10)H), 7.25 (d, *J* = 5 Hz, 1H, C(9)H).

^13^C–{^1^H} NMR (500 MHz, CDCl_3_): *δ* = −0.44 (C(12, 13, 14)), 17.03 (t, *J* = 19 Hz, C(6)), 40.23 (C(2)), 54.68 (C(7)), 59.01 (C(4)), 61.00 (C(1)), 116.51 (t, C(5)), 124.79 (C(9)), 125.53 (C(11)), 126.41 (C(10)), 142.07 (C(8)), 162.37 (C(3)).

MS (*m*/*z*, %): 268 (2) [M]^+^, 267 (7), 252 (6), 194 (62), 97 (100), 73 (40).

Anal. calcd for C_14_H_21_D_2_NSSi, (%): C, 62.86; N, 5.24. Found, %: C, 62.54; N, 5.20.

#### (*Z*)-3-Methyl-1-(4-methylbenzyl)-4-(4-methylbenzylidene)pyrrolidine (3i)

Using the procedure described above *N*-(4-methylbenzyl)-*N*-(3-(*p*-tolyl)prop-2-yn-1-yl)prop-2-en-1-amine (578 mg, 2 mmol) and H_2_O (instead of D_2_O)gave crude product that was purified by column chromatography (diethyl ether : isopropyl alcohol : hexane = 1 : 1 : 8) to afford 3i (265 mg, 91%). *R*_f_ 0.54.

^1^H NMR (500 MHz, CDCl_3_): *δ* = 1.27 (d, *J* = 7 Hz, 3H, C(6)H_3_), 2.17 (t, *J* = 8 Hz, 1H(A), C(1)H_2_), 2.39 (s, 3H, C(21)H_3_), 2.41 (s, 3H, C(20)H_3_), 2.96 (q, *J* = 7 Hz, 1H, C(2)H), 3.07 (t, *J* = 8 Hz, 1H(B), C(1)H_2_), 3.39 (d, *J* = 15 Hz, 1H(A), C(4)H_2_), 3.73 (s, 2H, C(7)H_2_), 3.86 (d, *J* = 15 Hz, 1H(B), C(4)H_2_), 6.26 (s, 1H, C(5)H), 7.17 (d, *J* = 5 Hz, 2H, C(14, 18)H), 7.18 (m, 2H, C(15, 17)H), 7.20 (d, *J* = 8 Hz, 2H, C(10, 12)H), 7.32 (d, *J* = 8 Hz, 2H, C(9, 13)H).

^13^C–{^1^H} NMR (500 MHz, CDCl_3_): *δ* = 18.04 (C(6)), 21.17 (C(20, 21)), 39.17 (C(2)), 58.54 (C(4)), 60.45 (C(7)), 61.33 (C(1)), 120.19 (C(5)), 127.86 (C(14, 18)), 128.72 (C(9, 13)), 129.03 (C(15, 17)), 129.07 (C(10, 12)), 135.38 (C(19)), 135.71 (C(16)), 136.54 (C(11)), 137.97 (C(8)).

MS (*m*/*z*, %): 291 (77) [M]^+^, 276 (30), 186 (11), 143 (15), 105 (100).

Anal. calcd for C_21_H_25_N, (%): C, 86.55; H, 8.65; N, 4.81. Found, %: C, 86.37; H, 8.60; N, 4.79.

#### (*E*)-3-(Iodo(trimethylsilyl)methylene)-4-(iodomethyl)-1-(4-methoxybenzyl)pyrrolidine (5a); typical procedure

To a solution of *N*-(4-methoxybenzyl)-*N*-(3-(trimethylsilyl)prop-2-yn-1-yl)prop-2-en-1-amine (754 mg, 2 mmol) and Et_2_Zn (1 M in hexanes, 5 mL, 5 mmol) in CH_2_Cl_2_ (6 mL) was added Ti(O-*i*Pr)_4_ (0.5 M in hexanes, 0.6 mL, 0.3 mmol). Ethylmagnesiurn bromide (2.5 M in Et_2_O, 0.16 mL, 0.4 mmol) was then added and the reaction mixture rapidly turned black. After 18 h at 23 °C, the reaction mixture was cooled to −78 °C, and a solution of I_2_ (1575 mg, 12.5 mmol) in THF (12.5 mL) was added *via* cannula. The reaction mixture was warmed to 23 °C, and stirred overnight. The mixture was then partitioned between 25% aqueous KOH and ether. The organic layer was washed with water and aqueous Na_2_S_2_O_3_, drying over MgSO_4_. Evaporation of solvent and purification of the residue by column chromatography (diethyl ether : isopropyl alcohol : hexane = 1 : 1 : 8) to afford 5a (617 mg, 57%). *R*_f_ 0.85.

^1^H NMR (500 MHz, CDCl_3_): *δ* = 0.40 (s, 9H, C(14, 15, 16)H_3_), 3.17 (m, 1H(A), C(6)IH_2_), 3.18 (m, 1H(A), C(1)H_2_), 3.27 (m, 1H, C(2)H), 3.45 (m, 1H(B), C(1)H_2_), 3.55 (dd, *J* = 10 Hz, *J* = 3 Hz, 1H(B), C(6)IH_2_), 3.83 (s, 3H, C(17)H_3_), 4.27 (d, *J* = 14 Hz, 1H(A), C(7)H_2_), 4.62 (d, *J* = 14 Hz, 1H(B), C(7)H_2_), 6.89 (d, *J* = 9 Hz, 2H, C(10, 12)H), 7.20 (d, *J* = 9 Hz, 2H, C(9, 13)H).

^13^C–{^1^H} NMR (500 MHz, CDCl_3_): *δ* = 2.21 (C(14, 15, 16)), 7.77 (C(6)), 47.03 (C(7)), 48.98 (C(2)), 49.22 (C(1)), 55.31 (C(17)), 114.20 (C(10, 12)), 127.77 (C(8)), 129.72 (C(9, 13)), 153.11 (C(3)), 159.31 (C(11)), 162.60 (C(4)).

Anal. calcd for C_17_H_23_I_2_NO_2_Si, (%): C, 36.77; H, 4.18; N, 2.52. Found, %: C, 36.21; H, 4.42; N, 2.39.

#### (*E*)-3-(Iodo(trimethylsilyl)methylene)-4-(iodomethyl)-1-(4-methylbenzyl)pyrrolidine (5h)

Using the procedure described above *N*-(4-methylbenzyl)-*N*-(3-(trimethylsilyl)prop-2-yn-1-yl)prop-2-en-1-amine (542 mg, 2 mmol) gave crude product that was purified by flash chromatography (diethyl ether : isopropyl alcohol : hexane = 1 : 1 : 8) to afford 5h (641 mg, 61%). *R*_f_ 0.87.

^1^H NMR (500 MHz, CDCl_3_): *δ* = 0.41 (s, 9H, C(14, 15, 16)H_3_), 2.36 (s, 3H, C(17)H_3_), 3.17 (m, 1H(A), C(6)IH_2_), 3.19 (m, 1H(A), C(1)H_2_), 3.27 (m, 1H, C(2)H), 3.46 (m, 1H(B), C(1)H_2_), 3.56 (dd, *J* = 10 Hz, *J* = 3 Hz, 1H(B), C(6)IH_2_), 4.28 (d, *J* = 14 Hz, 1H(A), C(7)H_2_), 4.66 (d, *J* = 14 Hz, 1H(B), C(7)H_2_), 7.17 (s, 4H, C(9, 10, 12, 13)H).

^13^C–{^1^H} NMR (500 MHz, CDCl_3_): *δ* = 2.22 (C(14, 15, 16)), 7.78 (C(6)), 21.17 (C(17)), 47.37 (C(7)), 49.09 (C(2)), 49.29 (C(1)), 125.63 (C(5)), 128.36 (C(9, 13)), 129.52 (C(10, 12)), 132.63 (C(8)), 137.64 (C(11)), 153.08 (C(3)), 162.66 (C(4)).

MS (*m*/*z*, %): 539 (4) [M]^+^, 420 (8), 396 (8), 292 (8), 105 (100), 79 (15).

Anal. calcd for C_17_H_23_I_2_NOSi, (%): C, 37.86; H, 4.30; N, 2.60. Found, %: C, 38.08; H, 4.27; N, 2.44.

### Preparation of bis-3-methyl-4-methylenepyrrolidines 7 and 9*via* Ti–Mg-catalyzed carbocyclization of bis-*N*-allyl substituted propargylamines with Et_2_Zn in CH_2_Cl_2_

#### 1,4-bis(((*Z*)-3-Methyl-4-((trimethylsilyl)methylene)pyrrolidin-1-yl)methyl)benzene (7); typical procedure

To a solution of *N*,*N*′-(1,4-phenylenebis(methylene))bis(*N*-(3-(trimethylsilyl)prop-2-yn-1-yl)prop-2-en-1-amine) (874 mg, 2 mmol) and Et_2_Zn (1 M in hexanes, 5 mL, 10 mmol) in CH_2_Cl_2_ (6 mL) was added Ti(O-*i*Pr)_4_ (0.5 M in hexanes, 1.2 mL, 0.6 mmol). Ethylmagnesiurn bromide (2.5 M in Et_2_O, 0.32 mL, 0.8 mmol) was then added and the reaction mixture rapidly turned black. After 18 h at 23 °C, the reaction mixture was diluted with Et_2_O (5 mL), and 25 wt% KOH solution (3 mL) was added dropwise while the reaction flask was cooled in an ice bath. The aqueous layer was extracted with diethyl ether (3 × 5 mL). The combined organic layers were washed with brine (10 mL), dried over anhydrous CaCl_2_. The reaction mixture was filtered through a filter paper and concentrated *in vacuo* to give crude product as a yellow oil. Evaporation of solvent and purification of the residue by column chromatography (diethyl ether : isopropyl alcohol : hexane = 1 : 1 : 8) gave 7 (776 mg, 88%) as colorless oil. *R*_f_ 0.52.

^1^H NMR (500 MHz, CDCl_3_): *δ* = 0.08 (s, 18H, C(11, 12, 13, 11′, 12′, 13′)H_3_), 1.09 (d, *J* = 7 Hz, 6H, C(6, 6′)H_3_), 2.01 (t, *J* = 9 Hz, 2H(A), C(1, 1′)H_2_), 2.67 (q, *J* = 7 Hz, 2H, C(2, 2′)H), 2.98 (t, *J* = 8 Hz, 2H(B), C(1, 1′)H_2_), 3.03 (d, *J* = 14 Hz, 2H(A), C(4, 4′)H_2_), 3.55 (d, *J* = 14 Hz, 2H(B), C(4, 4′)H_2_), 3.60 (d, *J* = 13 Hz, 2H(A), C(7, 7′)H_2_), 3.66 (d, *J* = 13 Hz, 2H(B), C(7, 7′)H_2_), 5.30 (s, 2H, C(5, 5′)H), 7.31 (s, 4H, C(9, 10, 9′, 10′)H).

^13^C–{^1^H} NMR (500 MHz, CDCl_3_): *δ* = −0.43 (C(11, 12, 13, 11′, 12′, 13′)), 17.36 (C(6, 6′)), 40.32 (2, 2′), 59.42 (C(4, 4′)), 60.55 (C(7, 7′)), 61.31 (C(1, 1′)), 116.69 (C(5, 5′)), 128.78 (C(9, 10, 9′, 10′)), 137.65 (C(8, 8′)), 162.77 (C(3, 3′)).

MS (EI): *m*/*z*, % = 441 (16) [M]^+^, 440 (39), 367 (100), 272 (66), 207 (44), 168 (34), 104 (85), 73 (67), 44 (47).

Anal. calcd for C_26_H_44_N_2_Si_2_, (%): C, 70.84; H, 10.06; N, 6.35. Found, %: C, 71.07; H, 9.95; N, 6.39.

#### (4*Z*,4′*Z*)-4,4′-(Octane-2,7-diylidene)bis(3-ethyl-1-(4-methylbenzyl)pyrrolidine) (9)

Using the procedure described above *N*^1^,*N*^10^-diallyl-*N*^1^,*N*^10^-bis(4-methylbenzyl)deca-2,8-diyne-1,10-diamine (906 mg, 2 mmol) gave crude product that was purified by flash chromatography (diethyl ether : isopropyl alcohol : hexane = 1 : 1 : 8) to afford 9 (872 mg, 85%). *R*_f_ 0.80.

^1^H NMR (500 MHz, CDCl_3_): *δ* = 1.10 (d, *J* = 7 Hz, 6H, C(6, 6′)H_3_), 1.36 (s, 4H, C(16, 16′)H_2_), 1.92 (d, *J* = 5 Hz, 4H, C(15, 15′)H_2_), 2.04 (t, *J* = 9 Hz, 2H(A), C(4, 4′)H_2_), 2.38 (s, 6H, C(14, 14′)), 2.70 (q, *J* = 7 Hz, 2H, C(3, 3′)H), 2.97 (d, *J* = 14 Hz, 2H(A), C(1, 1′)H_2_), 3.00 (t, *J* = 8 Hz, 2H(B), C(4, 4′)H_2_), 3.48 (d, *J* = 14 Hz, 2H(B), C(1, 1′)H_2_), 3.61 (d, *J* = 13 Hz, 2H(A), C(7, 7′)H_2_), 3.65 (d, *J* = 13 Hz, 2H(B), C(7, 7′)H_2_), 5.15 (m, 2H, C(5, 5′)H), 7.17 (d, *J* = 8 Hz, 4H, C(10, 12, 10′,12′)H), 7.28 (d, *J* = 8 Hz, 4H, C(9, 13, 9′, 13′)H).

^13^C–{^1^H} NMR (500 MHz, CDCl_3_): *δ* = 17.75 (C(6, 6′)), 21.15 (C(14, 14′)), 29.24 (C(16, 16′)), 29.37 (C(15, 15′)), 37.33 (C(3, 3′)), 56.82 (C(1, 1′)), 60.61 (C(7, 7′)), 62.28 (C(4, 4′)), 119.69 (C(5, 5′)), 128.77 (C(9, 13, 9′, 13′)), 128.94 (C(10, 12, 10′, 12′)), 136.07 (C(8, 8′)), 136.46 (C(11, 11′)), 144.09 (C(2, 2′)).

MS (*m*/*z*, %): 457 (3) [M]^+^, 456 (3), 351 (1), 200 (10), 105 (100), 79 (6).

Anal. calcd for C_32_H_44_N_2_, (%): C, 84.16; H, 9.71; N, 6.13. Found, %: C, 83.89; H, 9.50; N, 6.17.

### Preparation of 3-methyl-4-methylenepyrrolidines 11a–c, 12a and 14a*via* Ti–Mg-catalyzed carbocyclization of allyl substituted but-2-yne-1,4-diamines with Et_2_Zn in CH_2_Cl_2_

#### (*Z*)-*N*,*N*-Dimethyl-2-(4-methyl-1-(4-methylbenzyl)pyrrolidin-3-ylidene)ethan-1-amine (11a)

To a solution of *N*^1^-allyl-*N*^4^,*N*^4^-dimethyl-*N*^1^-(4-methylbenzyl)but-2-yne-1,4-diamine (512 mg, 2 mmol) and Et_2_Zn (1 M in hexanes, 5 mL, 5 mmol) in CH_2_Cl_2_ (6 mL) was added Ti(O-*i*Pr)_4_ (0.5 M in hexanes, 0.6 mL, 0.3 mmol). Ethylmagnesiurn bromide (2.5 M in Et_2_O, 0.16 mL, 0.4 mmol) was then added and the reaction mixture rapidly turned black. After 18 h at 23 °C, the reaction mixture was diluted with Et_2_O (5 mL), and 25 wt% KOH solution (3 mL) was added dropwise while the reaction flask was cooled in an ice bath. The aqueous layer was extracted with diethyl ether (3 × 5 mL). The combined organic layers were washed with brine (10 mL), dried over anhydrous CaCl_2_. The reaction mixture was filtered through a filter paper and concentrated *in vacuo* to give crude product as a yellow oil. Evaporation of solvent and purification of the residue by column chromatography (diethyl ether : isopropyl alcohol : hexane = 1 : 1 : 8) gave 11a (464 mg, 90%) as colorless oil. *R*_f_ 0.85.

^1^H NMR (500 MHz, CDCl_3_): *δ* = 1.12 (d, *J* = 7 Hz, 3H, C(6)H_3_), 2.07 (t, *J* = 9 Hz, 1H(A), C(1)H_2_), 2.35 (s, 6H, C(15, 16)H_3_), 2.36 (s, 3H, C(17)H_3_), 2.75 (q, *J* = 7 Hz, 1H, C(2)H), 2.99 (m, 1H(A), C(4)H_2_), 3.00 (m, 2H, C(14)H_2_), 3.02 (m, 1H(B), C(1)H_2_), 3.50 (d, *J* = 14 Hz, 1H(B), C(4)H_2_), 3.60 (d, *J* = 13 Hz, 1H(A), C(7)H_2_), 3.65 (d, *J* = 12 Hz, 1H(B), C(7)H_2_), 5.31 (m, 1H, C(5)H), 7.15 (d, *J* = 8 Hz, 2H, C(10, 12)H), 7.24 (d, *J* = 8 Hz, 2H, C(9, 13)H).

^13^C–{^1^H} NMR (500 MHz, CDCl_3_): *δ* = 17.49 (C(6)), 21.21 (C(17)), 37.69 (C(2)), 44.87 (C(15, 16)), 56.55 (C(4)), 57.66 (C(14)), 60.25 (C(7)), 61.64 (C(1)), 114.34 (C(5)), 128.77 (C(9, 13)), 129.02 (C(10, 12)), 135.36 (C(8)), 136.73 (C(11)), 149.76 (C(3)).

MS (EI): *m*/*z*, % = 258 (<1) [M^+^], 257 (<1), 213 (80), 198 (57), 105 (100).

Anal. calcd for C_17_H_26_N_2_, (%): C, 79.02; H, 10.14; N, 10.84. Found, %: C, 78.86; H, 10.09; N, 11.0.

#### (*Z*)-4-(2-(1-(4-Methoxybenzyl)-4-methylpyrrolidin-3-ylidene)ethyl)morpholine (11b)

Using the procedure described above *N*-allyl-*N*-(4-methoxybenzyl)-4-morpholinobut-2-yn-1-amine (628 mg, 2 mmol) gave crude product that was purified by column chromatography (diethyl ether : isopropyl alcohol : hexane = 1 : 1 : 8) to afford 11b (534 mg, 89%). *R*_f_ 0.48.

^1^H NMR (500 MHz, CDCl_3_): *δ* = 1.09 (d, *J* = 7 Hz, 3H, C(6)H_3_), 2.01 (t, *J* = 8 Hz, 1H(A), C(1)H_2_), 2.42 (s, 4H, C(15, 18)H_2_), 2.71 (q, *J* = 8 Hz, 1H, C(2)H), 2.88 (d, *J* = 6 Hz, 2H, C(14)H_2_), 2.93 (d, *J* = 14 Hz, 1H(A), C(4)H_2_), 2.97 (t, *J* = 8 Hz, 1H(B), C(1)H_2_), 3.47 (d, *J* = 14 Hz, 1H(B), C(4)H_2_), 3.55 (d, *J* = 13 Hz, 1H(A), C(7)H_2_), 3.59 (d, *J* = 13 Hz, 1H(B), C(7)H_2_), 3.71 (s, 4H, C(16, 17)H_2_), 3.80 (s, 3H, C(19)H_3_), 5.25 (s, 1H, C(5)H), 6.87 (d, *J* = 8 Hz, 2H, C(10, 12)H), 7.25 (d, *J* = 8 Hz, 2H, C(9, 13)H).

^13^C–{^1^H} NMR (500 MHz, CDCl_3_): *δ* = 17.53 (C(6)), 37.66 (C(2)), 53.61 (C(15, 18)), 55.21 (C(19)), 56.74 (C(4)), 57.82 (C(14)), 60.04 (C(7)), 61.78 (C(1)), 66.99 (C(16, 17)), 113.63 (C(10, 12)), 115.49 (C(5)), 129.89 (C(9, 13)), 130.93 (C(8)), 148.32 (C(3)), 158.66 (C(11)).

MS (EI): *m*/*z*, % = 316 (<1) [M]^+^, 229 (39), 121 (100), 77 (4).

Anal. calcd for C_19_H_28_N_2_O_2_, (%): C, 72.12; H, 8.92; N, 8.85. Found, %: C, 72.15; H, 8.79; N, 8.49.

#### (*Z*)-1-(2-(1-(4-Methoxybenzyl)-4-methylpyrrolidin-3-ylidene)ethyl)piperidine (11c)

Using the procedure described above *N*-allyl-*N*-(4-methoxybenzyl)-4-(piperidin-1-yl)but-2-yn-1-amine (624 mg, 2 mmol) gave crude product that was purified by column chromatography (diethyl ether : isopropyl alcohol : hexane = 1 : 1 : 8) to afford 11c (515 mg, 82%). *R*_f_ 0.79.

^1^H NMR (500 MHz, CDCl_3_): *δ* = 1.04 (d, *J* = 7 Hz, 3H, C(6)H_3_), 1.44 (s, 2H, C(17)H_2_), 1.59 (p, *J* = 6 Hz, 4H, C(16, 18)), 1.99 (t, *J* = 9 Hz, 1H(A), C(1)H_2_), 2.36 (s, 4H, C(15, 19)H_2_), 2.71 (q, *J* = 8 Hz, 1H, C(2)H), 2.84 (d, *J* = 7 Hz, 2H, C(14)H_2_), 2.93 (d, *J* = 14 Hz, 1H(A), C(4)H_2_), 2.97 (t, *J* = 8 Hz, 1H(B), C(1)H_2_), 3.47 (d, *J* = 14 Hz, 1H(B), C(4)H_2_), 3.55 (d, *J* = 13 Hz, 1H(A), C(7)H_2_), 3.59 (d, *J* = 13 Hz, 1H(B), C(7)H_2_), 3.81 (s, 3H, C(20)H_3_), 5.29 (m, 1H, C(5)H), 6.87 (d, *J* = 8 Hz, 2H, C(10, 12)H), 7.26 (d, *J* = 8 Hz, 2H, C(9, 13)H).

^13^C–{^1^H} NMR (500 MHz, CDCl_3_): *δ* = 17.49 (C(6)), 24.39 (C(17)), 25.95 (C(16, 18)), 37.62 (C(2)), 54.52 (C(15, 19)), 55.23 (C(20)), 56.77 (C(4)), 58.24 (C(14)), 60.10 (C(7)), 61.86 (C(1)), 113.61 (C(10, 12)), 116.53 (C(5)), 129.91 (C(9, 13)), 131.05 (C(8)), 147.19 (C(3)), 158.63 (C(11)).

MS (EI): *m*/*z*, % = 314 (<1) [M^+^], 121 (100), 77 (5).

Anal. calcd for C_20_H_30_N_2_O, (%): C, 76.39; H, 9.62; N, 8.91. Found, %: C, 76.44; H, 9.86; N, 8.59.

#### (*Z*)-*N*,*N*-Dimethyl-2-(4-(methyl-*d*)-1-(4-methylbenzyl)pyrrolidin-3-ylidene)ethan-1-amine-2-*d* (12a)

Using the procedure described above *N*^1^-allyl-*N*^4^,*N*^4^-dimethyl-*N*^1^-(4-methylbenzyl)but-2-yne-1,4-diamine (512 mg, 2 mmol) and D_2_O (instead of H_2_O)gave crude product that was purified by column chromatography (diethyl ether : isopropyl alcohol : hexane = 1 : 1 : 8) to afford 12a (480 mg, 88%). *R*_f_ 0.85.

^1^H NMR (500 MHz, CDCl_3_): *δ* = 1.10 (m, 2H, C(6)DH_2_), 2.04 (t, *J* = 8 Hz, 1H(A), C(1)H_2_), 2.23 (s, 6H, C(15, 16)H_3_), 2.37 (s, 3H, C(17)H_3_), 2.73 (p, *J* = 7 Hz, 1H, C(2)H), 2.83 (s, 2H, C(14)H_2_), 2.96 (d, *J* = 14 Hz, 1H(A), C(4)H_2_), 2.99 (t, *J* = 8 Hz, 1H(B), C(1)H_2_), 3.49 (d, *J* = 14 Hz, 1H(B), C(4)H_2_), 3.59 (d, *J* = 13 Hz, 1H(A), C(7)H_2_), 3.64 (d, *J* = 13 Hz, 1H(B), C(7)H_2_), 7.15 (d, *J* = 8 Hz, 2H, C(10, 12)H), 7.25 (d, *J* = 8 Hz, 2H, C(9, 13)H).

^13^C–{^1^H} NMR (500 MHz, CDCl_3_): *δ* = 17.39 (t, *J* = 19 Hz, C(6)), 21.12 (C(17)), 37.50 (C(2)), 45.11 (C(15, 16)), 56.69 (C(4)), 58.29 (C(14)), 60.45 (C(7)), 61.88 (C(1)), 128.72 (C(9, 13)), 128.96 (C(10, 12)), 135.84 (C(8)), 136.55 (C(11)), 147.53 (C(3)).

MS (EI): *m*/*z*, % = 260 (<1) [M]^+^, 215 (36), 199 (30), 105 (100), 79 (7).

Anal. calcd for C_17_H_24_D_2_N_2_, (%): C, 78.41; N, 10.76. Found, %: C, 78.48; N, 11.08.

#### (*Z*)-3-(5-Methoxypentylidene)-4-methyl-1-(4-methylbenzyl)pyrrolidine (14)

Using the procedure described above *N*-allyl-7-methoxy-*N*-(4-methylbenzyl)hept-2-yn-1-amine (570 mg, 2 mmol) and H_2_O (instead of D_2_O)gave crude product that was purified by column chromatography (diethyl ether : isopropyl alcohol : hexane = 1 : 1 : 8) to afford 14 (517 mg, 90%). *R*_f_ 0.63.

^1^H NMR (500 MHz, CDCl_3_): *δ* = 1.08 (d, *J* = 7 Hz, 3H, C(6)H_3_), 1.42 (p, *J* = 8 Hz, 2H, C(15)H_2_), 1.58 (p, *J* = 8 Hz, 2H, C(16)H_2_), 1.95 (q, *J* = 7 Hz, 2H, C(14)H_2_), 2.03 (t, *J* = 8 Hz, 1H(A), C(1)H_2_), 2.37 (s, 3H, C(19)H_3_), 2.96 (d, *J* = 13 Hz, 1H(A), C(4)H_2_), 2.99 (t, *J* = 8 Hz, C(1)H_2_), 3.34 (s, 3H, C(18)H_3_), 3.37 (t, *J* = 7 Hz, 2H, C(17)H_2_), 3.47 (d, *J* = 13 Hz, 1H(B), C(4)H_2_), 3.59 (d, *J* = 13 Hz, 1H(A), C(7)H_2_), 3.64 (d, *J* = 13 Hz, 1H(B), C(7)H_2_), 5.14 (m, 1H, C(5)H), 7.15 (d, *J* = 8 Hz, 2H, C(10, 12)H), 7.26 (d, *J* = 8 Hz, 2H, C(9, 13)H).

^13^C–{^1^H} NMR (500 MHz, CDCl_3_): *δ* = 17.68 (C(6)), 21.12 (C(19)), 26.08 (C(15)), 29.20 (C(14)), 29.25 (C(16)), 37.30 (C(2)), 56.73 (C(4)), 58.55 (C(18)), 60.54 (C(7)), 62.22 (C(1)), 119.43 (C(5)), 128.77 (C(9, 13)), 128.93 (C(10, 12)), 135.95 (C(8)), 136.47 (C(11)), 144.32 (C(3)).

MS (EI): *m*/*z*, % = 287 (18) [M^+^], 200 (38), 105 (100), 79 (9).

Anal. calcd for C_19_H_29_NO, (%): C, 79.39; H, 10.17; N, 4.87. Found, %: C, 79.11; H, 10.00; N, 4.53.

## Conflicts of interest

The authors declare no competing financial interest.

## Supplementary Material

RA-010-D0RA02677H-s001
