# Supplementary material for: Synthesis of heteroatom-containing pyrrolidine derivatives based on Ti(O-iPr)4 and EtMgBr-catalyzed carbocyclization of allylpropargyl amines with Et2Zn
Source: RSC Adv. 2020 May 7;10(30):17881–91. doi: 10.1039/d0ra02677h (PMC9053609; doi:10.1039/d0ra02677h)

Electronic Supplementary Material (ESI) for RSC Advances.
This journal is © The Royal Society of Chemistry 2020

Synthesis of Heteroatom-Containing Pyrrolidine Derivatives Based on Ti(O-*i*Pr)$_4$ and EtMgBr-Catalyzed Carbocyclization of Allylpropargyl Amines with Et_2Zn

*Rita N. Kadikova, Ilfir R. Ramazanov, Azat M. Gabdullin, Oleg S. Mozgovoj, Usein M. Dzhemilev

Institute of Petrochemistry and Catalysis of Russian Academy of Sciences, 141 Prospekt Oktyabrya, Ufa 450075, Russian Federation

Tel./fax: +7-347-284-2750

E-mail address: kadikritan@gmail.com

Supporting information

Reagents and methods

The reagents were obtained from Sigma-Aldrich or Acros. Hexane and dichloromethane were distilled over P_2O_5. Diethyl ether, tetrahydrofuran, 1,4-dioxane, toluene, benzene and anisole were dried over sodium. Dried 1,2-dimethoxyethane was obtained from Sigma-Aldrich. 2-Alkynylamines **1a**-**i** and **6,8** were prepared by aminomethylation of terminal alkynes with aqueous formaldehyde and secondary *N*-aryl-substituted allyl amines under CuBr catalysis [1]. Nitrogen-containing 1,6-enynes with terminal propargyl and allyl groups were prepared by alkylation of *N*-aryl-substituted allyl amines with propargyl bromide under NaH [2]. Allyl substituted but-2-yne-1,4-diamines **10** were prepared by aminomethylation of nitrogen-containing 1,6-enynes (with terminal propargyl and allyl groups) by bisamine [3]. Acetylenic ethers **13** were prepared by aminomethylation of ethers of acetylenic alcohols with aqueous formaldehyde and secondary *N*-aryl- substituted allyl amines under CuBr catalysis [1]. Nuclear magnetic resonance spectroscopy was performed on a Brucker Avance 500. The ^1H NMR spectra were recorded at 500 MHz and ^{13}C-{^1H} NMR spectra at 100 MHz in $CDCl_3$. The chemical shifts are reported in ppm relative to tetramethylsilane (TMS) as the internal

standard. The numbering of atoms in the ^{13}C-{^{1}H} and ^{1}H NMR spectra of the compounds **3a-g**, **3i**, **4h**,**f** **5a**, **5h**, **7**, **9**, **11a-c**, **12a**, **14** is shown in Figures 1,2,3. Elemental analysis was performed using a Carlo-Erba CHN 1106 elemental analyser. Mass spectra were obtained on a Finnigan 4021 instrument. The yields were calculated from the isolated amount of pyrrolidine and pyrrolidone derivatives obtained from starting nitrogen-containing 1,6-enynes.

Preparation of 3-methyl-4-methylenepyrrolidines 3a-g, 3i, 4h,f and 5a,h via Ti-Mg-catalyzed carbozincation of *N*-allyl substituted propargylamines with Et_2Zn in CH_2Cl_2.

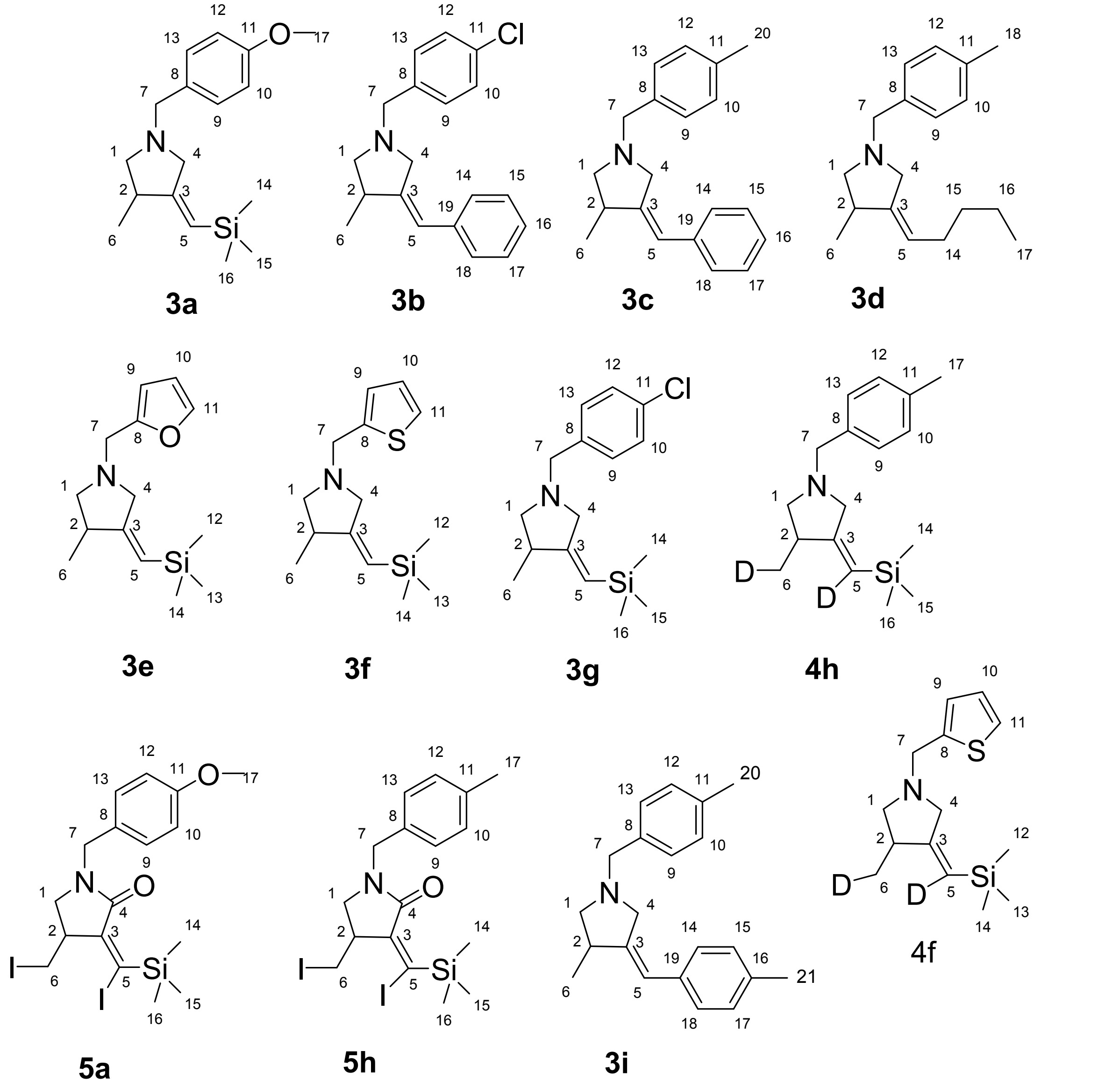

Figure 1 The numbering of atoms in the ^{13}C- and ^{1}H-NMR spectra of the compounds **3a-g, 3i, 4h,f and 5a,h.**

(*Z*)-1-(4-methoxybenzyl)-3-methyl-4-((trimethylsilyl)methylene)pyrrolidine; Typical Procedure.

To a solution of *N*-(4-methoxybenzyl)-*N*-(3-(trimethylsilyl)prop-2-yn-1-yl)prop-2-en-1-amine (574 mg, 2 mmol) and Et_2Zn (1 M in hexanes, 5 mL, 5 mmol) in CH_2Cl_2 (6 mL) was added Ti(O-*i*Pr)$_4$ (0.5 M in hexanes, 0.6 mL, 0.3 mmol). Ethylmagnesiurn bromide (2.5 M in Et_2O, 0.16 mL, 0.4 mmol) was then added and the reaction mixture rapidly turned black. After 18 h at 23 ^oC, the reaction mixture was diluted with Et_2O (5 mL), and 25 wt% KOH solution (3 mL) was added dropwise while the reaction flask was cooled in an ice bath. The aqueous layer was extracted with diethyl ether (3×5 mL). The combined organic layers were washed with brine (10 mL), dried over anhydrous $CaCl_2$. The reaction mixture was filtered through a filter paper and concentrated in vacuo to give crude product as a yellow oil. Evaporation of solvent and purification of the residue by column chromatography (diethyl ether : isopropyl alcohol : hexane = 1 : 1 :8) gave **3a** (509 mg, 88 %) as colorless oil. R_f 0.70.

^{1}H NMR (500MHz, $CDCl_3$): δ = 0.09 (s, 9H, C(14, 15,16)H$_3$), 1.09 (d, *J* = 7 Hz, 3H, C(6)H$_3$), 1.99 (t, *J* = 8 Hz, 1H(A), C(1)H$_2$), 2.68 (q, *J* = 7 Hz, 1H, C(2)H), 2.98 (t, *J* = 8 Hz, 1H(B), C(1)H$_2$), 3.03 (dt, *J* = 14 Hz, *J* = 2 Hz, 2H, C(4)H$_2$), 3.56 (d, *J* = 12Hz, 1H(A), C(7)H$_2$), 3.63 (d, *J* = 12Hz, 1H(B), C(7)H$_2$), 3.82 (s, 3H, C(17)H$_3$), 5.31 (q, *J* = 2 Hz, 1H, C(5)H), 6.89 (d, *J* = 8 Hz, 2H, C(10, 12)H), 7.28 (d, *J* = 8 Hz, 2H, C(9, 13)H).

^{13}C-{^{1}H} NMR (500MHz, $CDCl_3$): δ = -0.40 (C(14, 15, 16)), 17.34 (C(6)), 40.28 (C(2)), 55.22 (C(17)), 59.32 (C(4)), 60.12 (C(7)), 61.15 (C(1)), 113.63 (C(10, 12)), 116,74 (C(5)), 130.04 (C(9, 13)), 131.66 (C(8)), 158.68 (C(11)), 162.68 (C(3)).

MS (EI): m/z, % = 289 (1) [M$^+$], 287 (11), 214 (11), 166 (8), 121 (100).

Anal. calcd for $C_{17}H_{27}NOSi$, (%): C, 70.53; H, 9.40; N, 4.84. Found, %: C, 70.76; H, 9.57; N, 5.07.

***(Z)*-3-benzylidene-1-(4-chlorobenzyl)-4-methylpyrrolidine (3b)**

Using the procedure described above *N*-(4-chlorobenzyl)-*N*-(3-phenylprop-2-yn-1-yl)prop-2-en-1-amine (592 mg, 2 mmol) gave crude product that was purified by column chromatography (diethyl ether : isopropyl alcohol : hexane = 1 : 1 :8) to afford **3b** (471 mg, 79%) as colorless oil. R_f 0.59.

^{1}H NMR (500MHz, $CDCl_3$): δ = 1.30 (t, *J* = 6 Hz, 3H, C(6)H$_3$), 2.19 (m, 1H(A), C(1)H$_2$), 2.97 (s, 1H, C(2)H), 3.05 (m, 1H(B), C(1)H$_2$), 3.40 (d, *J* = 14 Hz, 1H(A), C(4)H$_2$), 3.70 (s, 2H, C(7)H$_2$), 3.82 (d, *J* = 14 Hz, 1H(B), C(4)H$_2$), 6.32 (s, 1H, C(5)H), 7.25 (m, 1H, C(16)H), 7.26 (m, 2H, C(14, 18)H), 7.36 (m, 2H, C(9, 13)H), 7.38 (m, 2H, C(10, 12)H), 7.40 (m, 2H, C(15, 17)H).

^{13}C-{^{1}H} NMR (500MHz, $CDCl_3$): δ = 18.10 (C(6)), 39.20 (C(2)), 58.43 (C(4)), 59.93 (C(7)), 61.23 (C(1)), 120.69 (C(5)), 126.27 (C(16)), 127.97 (C(14, 18)),

128.45 (C(15, 17)), 128.54 (C(10, 12)), 130.12 (C(9, 13)), 132.79 (C(11)), 137.40 (C(8)), 138.05 (C(19)), 146.98 (C(3)).

Anal. calcd for $C_{19}H_{20}ClN$, (%): C, 76.62; H, 6.77; N, 4.70. Found, %: C, 76.45; H, 6.91; N, 4.75.

(*Z*)-3-benzylidene-4-methyl-1-(4-methylbenzyl)pyrrolidine (3c)

Using the procedure described above *N*-(4-methylbenzyl)-*N*-(3-phenylprop-2-yn-1-yl)prop-2-en-1-amine (380 mg, 2 mmol) gave crude product that was purified by column chromatography (diethyl ether : isopropyl alcohol : hexane = 1 : 1 :8) to afford **3c** (382 mg, 69%) as colorless oil. R_f 0.61.

^{1}H NMR (500MHz, $CDCl_3$): δ = 1.33 (d, *J* = 7 Hz, 3H, C(6)H_3), 2.23 (t, *J* = 8 Hz, 1H(A), C(1)H_2), 2.45 (s, 3H, C(20)H_3), 3.02 (q, *J* = 7 Hz, 1HC(2)H), 3.11 (t, *J* = 8 Hz, 1H(B), C(1)H_2), 3.46 (d, *J* = 15 Hz, 1H(A), C(4)H_2), 3.77 (s, 2H, C(7)H), 3.92 (d, *J* = 15 Hz, 1H(B), C(4)H_2), 6.34 (s, 1H, C(5)H), 7.25 (d, J = 8 Hz, 2H, C(10, 12)H), 7.27 (m, 1H, C(16)H), 7.30 (d, *J* = 8 Hz, 2H, C(14, 18)H), 7.37 (d, *J* = 8 Hz, 2H, C(9, 13)H), 7.42 (d, *J* = 8 Hz, 2H, C(15, 17)H).

^{13}C-{^{1}H} NMR (500MHz, $CDCl_3$): δ = 18.13 (C(6)), 21.23 (C(20)), 39.31 (C(2)), 58.59 (C(4)), 60.46 (C(7)), 61.32 (C(1)), 120.45 (C(5)), 126.14 (C(16)), 128.00 (C(14, 18)), 128.41 (C(15, 17)), 128.75 (C(9, 13)), 129.09 (C(10, 11)), 136.00 (C(8)), 136.58 (C(11)), 138.23 (C(19)), 147.59 (C(3)).

MS (*m/z*, %): 277 (41) $[M]^+$, 262 (19), 172 (10), 129 (13), 105 (100).

Anal. calcd for $C_{20}H_{23}N$, (%): C, 86.59; H, 8.36; N, 5.05. Found, %: C, 86.62; H, 8.43; N, 4.85.

(*Z*)-3-methyl-1-(4-methylbenzyl)-4-pentylidenepyrrolidine (3d)

Using the procedure described above *N*-allyl-*N*-(4-methylbenzyl)hept-2-yn-1-amine (510 mg, 2 mmol) gave crude product that was purified by column chromatography (diethyl ether : isopropyl alcohol : hexane = 1 : 1 :8) to afford **3d** (494 mg, 73 %). R_f 0.68.

^{1}H NMR (500MHz, $CDCl_3$): δ = 0.91 (m, 3H, C(17)H_3), 1.08 (d, *J* = 7 Hz, 3H, C(6)H_3), 1.31 (m, 2H, C(16)H_2), 1.33 (m, 2H, C(14)H_2), 1.92 (q, *J* = 7 Hz, 2H, C(15)H_2), 2.05 (m, 1H(A),C(1)H_2), 2.37 (s, 3H, C(18)H_3), 2.69 (q, *J* = 7 Hz, 1H, C(2)H), 2.98 (d, *J* = 14 Hz, 1H(A), C(4)H_2), 3.01 (t, *J* = 8 Hz, 1H(B), C(1)H_2), 3.50 (d, *J* = 14 H, 1H(B), C(4)H_2), 3.62 (d, *J* = 13 Hz, 1H(A), C(7)H_2), 3.66 (d, *J* = 13 Hz, 1H(B), C(7)H_2), 5.15 (m, 1H, C(5)H), 7.26 (d, *J* = 8 Hz, 2H, C(9, 13)H), 7.16 (d, *J* = 8 Hz, 2H, C(10, 12)H).

^{13}C-{^{1}H} NMR (500MHz, $CDCl_3$): δ = 14.03 (C(17)), 17.59 (C(6)), 21.12 (C(18)), 22.35 (C(16)), 29.14 (C(15)), 31.75 (C(14)), 37.17 (C(2)), 56.59 (C(4)), 60.43 (C(7)), 62.07 (C(1)), 120.05 (C(5)), 136.61 (C(8)), 128.87 (C(9, 13)), 128.96 (C(10, 12)), 143.76 (C(3)).

MS (*m/z*, %): 257 (14) $[M]^+$, 200 (25), 152 (10), 105 (100).

Anal.calcd for $C_{18}H_{27}N$, (%): C, 83.99; H, 10.57; N, 5.44. Found, %: C, 84.28; H, 10.73; N, 5.30.

(*Z*)-1-(furan-2-ylmethyl)-3-methyl-4-((trimethylsilyl)methylene)pyrrolidine (3e)

Using the procedure described above *N*-(furan-2-ylmethyl)-*N*-(3-(trimethylsilyl)prop-2-yn-1-yl)prop-2-en-1-amine (494 mg, 2 mmol) gave crude product that was purified by column chromatography (diethyl ether : isopropyl alcohol : hexane = 1 : 1 :8) to afford **3e** (403 mg, 81 %). R_f 0.74.

^{1}H NMR (500MHz, $CDCl_3$): δ = 0.09 (s, 9H, C(12, 13, 14)H_3), 1.09 (d, *J* = 7 Hz, 3H, C(6)H_3), 2.05 (t, *J* = 9 Hz, 1H(A), C(1)H_2), 2.70 (q, *J* = 7 Hz, C(2)H), 3.04 (m, 1H(B), C(1)H_2), 3.06 (m, 1H(A), C(4)H_2), 3.59 (dd, *J* = 14 Hz, *J* = 2Hz, 1H(B), C(4)H_2), 3.65 (d, *J* = 14 Hz, 1H(A), C(7)H_2), 3.68 (d, *J* = 14 Hz, 1H(B), C(7)H_2), 5.30 (m, 1H, C(5)H), 6.22 (d, *J* = 3 Hz, 1H, C(9)H), 6.34 (m, 1H, C(10)H), 7.39 (dd, *J* = 2 Hz, *J* = 1 Hz, 1H, C(11)H).

^{13}C-{^{1}H} NMR (500MHz, $CDCl_3$): δ = -0.45 (C(12, 13, 14)), 17.17 (C(6)), 40.25 (C(2)), 52.19 (C(7)), 58.96 (C(4)), 107.86 (C(9)), 110.06 (C(10)), 116.84 (C(5)), 141.97 (C(11)), 152.45 (C(8)), 162.29 (C(3)).

MS (*m/z*, %): 249 (16) $[M]^+$, 176 (76), 152 (9), 81 (100).

Anal.calcd for $C_{14}H_{23}NOSi$, (%): C, 67.42; H, 9.29; N, 5.62. Found, %: C, 67.07; H, 9.14; N, 5.39.

(*Z*)-3-methyl-1-(thiophen-2-ylmethyl)-4-((trimethylsilyl)methylene)pyrrolidine (3f)

Using the procedure described above *N*-(thiophen-2-ylmethyl)-N-(3-(trimethylsilyl)prop-2-yn-1-yl)prop-2-en-1-amine (526 mg, 2 mmol) gave crude product that was purified by column chromatography (diethyl ether : isopropyl alcohol : hexane = 1 : 1 :8) to afford **3f** (403 mg, 76 %). R_f 0.80.

^{1}H NMR (500MHz, $CDCl_3$): δ = 0.09 (s, 9H, C(12, 13, 14)H_3), 1.10 (d, *J* = 7 Hz, 3H, C(6)H_3), 2.07 (t, *J* = 8 Hz, 1H(A), C(1)H_2), 2.70 (m, 1H, C(2)H), 3.04 (t, *J* = 8 Hz, 1H(B), C(1)H_2), 3.09 (dt, *J* = 14 Hz, *J* = 2 Hz, 1H(A), C(4)H_2), 3.61 (dd, *J* = 14 Hz, *J* = 2 Hz, 1H(B), C(4)H_2), 3.84 (d, *J* = 14 Hz, 1H(A), C(7)H_2), 3.88 (d, *J* = 14 Hz, 1H(B), C(7)H_2), 5.32 (m, 1H, C(5)H), 6.96 (m, 1H, C(11)H), 6.98 (t, *J* = 3 Hz, 1H, C(10)H), 7.25 (dd, *J* = 5 Hz, *J* = 1 Hz, 1H, C(9)H).

^{13}C-{^{1}H} NMR (500MHz, $CDCl_3$): δ = -0.42 (C(12, 13, 14)), 17.34 (C(6)), 40.35 (C(2)), 54.69 (C(7)), 59.06 (C(4)), 61.04 (C(1)), 116.89 (C(5)), 124.79 (C(9)), 125.50 (C(11)), 126.41 (C(10)), 142.10 (C(8)), 162.44 (C(3)).

MS (*m/z*, %): 265 (4) $[M]^+$, 192 (31), 97 (100), 73 (20).

Anal.calcd for $C_{14}H_{23}NSSi$, (%): C, 63.34; H, 8.73; N, 5.28. Found, %: C, 63.39; H, 8.64; N, 5.11.

(*Z*)-1-(4-chlorobenzyl)-3-methyl-4-((trimethylsilyl)methylene)pyrrolidine (3g)

Using the procedure described above *N*-(4-chlorobenzyl)-*N*-(3-(trimethylsilyl)prop-2-yn-1-yl)prop-2-en-1-amine (584 mg, 2 mmol) gave crude product that was purified by column chromatography (diethyl ether : isopropyl alcohol : hexane = 1 : 1 :8) to afford **3g** (500 mg, 85 %). R_f 0.68.

^{1}H NMR (500MHz, $CDCl_3$): δ = 0.08 (s, 9H, C(14, 15, 16)H_3), 1.09 (d, *J* = 7 Hz, 3H, C(6)H_3), 2.01 (t, *J* = 8 Hz, 1H(A), C(1)H_2), 2.67 (p, *J* = 7 Hz, 1H, C(2)H), 2.95 (t, *J* = 8 Hz, 1H(B), C(1)H_2), 3.03 (dt, *J* = 14 Hz, *J* = 2 Hz, 1H(A), C(4)H_2), 3.52 (dd, *J* = 14 Hz, *J* = 2 Hz, 1H(B), C(4)H_2), 3.58 (d, *J* = 13 Hz, 1H(A), C(7)H_2), 3.63 (d, *J* = 13 Hz, 1H(B), C(7)H_2), 5.32 (m, 1H, C(5)H), 7.30 (d, *J* = 3 Hz, 4H, C(9, 10, 12, 13)H).

^{13}C-{^{1}H} NMR (500MHz, $CDCl_3$): δ = -0.3 (C(14, 15, 16)), 17.45 (C(6)), 40.36 (C(2)), 59.32 (C(4)), 60.01 (C(7)), 61.26 (C(1)), 116.96 (C(5)), 128.39 (C(10, 12)), 130.06 (C(9, 13)), 132.63 (C(11)), 137.46 (C(8)), 162.45 (C(3)).

MS (*m/z*, %): 294 (4) $[M]^+$, 293 (9), 220 (73), 168 (13), 125 (100), 89 (13), 73 (29).

Anal.calcd for $C_{16}H_{24}ClNSi$, (%): C, 65.39; H, 8.23; N, 4.77. Found, %: C, 65.43; H, 8.27; N, 5.01.

(*Z*)-3-(methyl-*d*)-1-(4-methylbenzyl)-4-((trimethylsilyl)methylene-*d*)pyrrolidine (4h)

Using the procedure described above *N*-(4-methylbenzyl)-*N*-(3-(trimethylsilyl)prop-2-yn-1-yl)prop-2-en-1-amine (542 mg, 2 mmol) and D_2O (instead of H_2O)gave crude product that was purified by column chromatography (diethyl ether : isopropyl alcohol : hexane = 1 : 1 :8) to afford **4h** (226 mg, 82 %). R_f 0.63.

^{1}H NMR (500MHz, $CDCl_3$): δ = 0.10 (s, 1H, C(14, 15, 16)H_3), 1.09 (t, *J* = 8 Hz, 2H, C(6)DH_2), 2.01 (t, *J* = 8 Hz, 1H(A), C(1)H_2), 2.38 (s, 3H, C(17)H_3), 2.68 (p, *J* = 7 Hz, 1H, C(2)H), 2.99 (t, *J* = 8 Hz, 1H(B), C(1)H_2), 3.06 (d, *J* = 14 Hz, 2H, C(4)H_2), 3.58 (d, *J* = 13 Hz, 1H(A), C(7)H_2), 3.67 (d, *J* = 13 Hz, 1H(B), C(7)H_2), 7.17 (d, *J* = 8 Hz, 2H, C(10, 12)H), 7.26 (d, *J* = 8 Hz, 2H, C(9,13)H).

^{13}C-{^{1}H} NMR (500MHz, $CDCl_3$): δ = -0.41 (C(14, 15, 16)), 17.07 (t, *J* = 20 Hz, C(6)), 21.14 (C(17)), 40.22 (C(2)), 59.39 (C(4)), 60.49 (C(7)), 61.17 (C(1)), 116.70 (C(5)), 128.82 (C(9, 13)), 128.96 (C(10, 12)), 135.67 (C(8)), 136.53 (C(11)), 162.69 (C(3)).

MS (*m/z*, %): 276 (<1) $[M]^+$, 275 (<1), 258 (6), 200 (41), 105 (100), 73 (15).

Anal.calcd for $C_{17}H_{25}D_2NSi$, (%): C, 74.11; N, 5.08. Found, %: C, 74.53; N, 5.30.

(*Z*)-3-(methyl-*d*)-1-(thiophen-2-ylmethyl)-4-((trimethylsilyl)methylene-*d*)pyrrolidine (4f)

Using the procedure described above *N*-(thiophen-2-ylmethyl)-*N*-(3-(trimethylsilyl)prop-2-yn-1-yl)prop-2-en-1-amine (526 mg, 2 mmol) and D_2O gave crude product that was purified by column chromatography (diethyl ether : isopropyl alcohol : hexane = 1 : 1 :8) to afford **4f** (400 mg, 71 %). R_f 0.80.

^{1}H NMR (500MHz, $CDCl_3$): δ = 0.09 (s, 9H, C(12, 13, 14)H_3), 1.08 (d, *J* = 7 Hz, 3H, C(6)DH_2), 2.06 (t, *J* = 8 Hz, 1H(A), C(1)H_2), 2.69 (m, 1H, C(2)H), 3.04 (t, *J* = 8 Hz, 1H(B), C(1)H_2), 3.09 (dt, *J* = 14 Hz, *J* = 2 Hz, 1H(A), C(4)H_2), 3.61 (d, *J* = 14 Hz, 1H(B), C(4)H_2), 3.84 (d, *J* = 14 Hz, 1H(A), C(7)H_2), 3.88 (d, *J* = 14 Hz, 1H(B), C(7)H_2), 6.95 (m, 1H, C(11)H), 6.97 (t, *J* = 3 Hz, 1H, C(10)H), 7.25 (d, *J* = 5 Hz, 1H, C(9)H).

^{13}C-{^{1}H} NMR (500MHz, $CDCl_3$): δ = -0.44 (C(12, 13, 14)), 17.03 (t, *J* = 19 Hz, C(6)), 40.23 (C(2)), 54.68 (C(7)), 59.01 (C(4)), 61.00 (C(1)), 116.51 (t, C(5)), 124.79 (C(9)), 125.53 (C(11)), 126.41 (C(10)), 142.07 (C(8)), 162.37 (C(3)).

MS (*m/z*, %): 268 (2) $[M]^+$, 267 (7), 252 (6), 194 (62), 97 (100), 73 (40).

Anal.calcd for $C_{14}H_{21}D_2NSSi$, (%): C, 62.86; N, 5.24. Found, %: C, 62.54; N, 5.20.

(*Z*)-3-methyl-1-(4-methylbenzyl)-4-(4-methylbenzylidene)pyrrolidine (3i)

Using the procedure described above *N*-(4-methylbenzyl)-*N*-(3-(*p*-tolyl)prop-2-yn-1-yl)prop-2-en-1-amine (578 mg, 2 mmol) and H_2O (instead of D_2O)gave crude product that was purified by column chromatography (diethyl ether : isopropyl alcohol : hexane = 1 : 1 :8) to afford **3i** (265 mg, 91 %). R_f 0.54.

^{1}H NMR (500MHz, $CDCl_3$): δ = 1.27 (d, *J* = 7 Hz, 3H, C(6)H_3), 2.17 (t, *J* = 8 Hz, 1H(A), C(1)H_2), 2.39 (s, 3H, C(21)H_3), 2.41 (s, 3H, C(20)H_3), 2.96 (q, *J* = 7 Hz, 1H, C(2)H), 3.07 (t, *J* = 8 Hz, 1H(B), C(1)H_2), 3.39 (d, *J* = 15 Hz, 1H(A), C(4)H_2), 3.73 (s, 2H, C(7)H_2), 3.86 (d, *J* = 15 Hz, 1H(B), C(4)H_2), 6.26 (s, 1H, C(5)H), 7.17 (d, *J* = 5Hz, 2H, C(14, 18)H), 7.18 (m, 2H, C(15, 17)H), 7.20 (d, *J* = 8 Hz, 2H, C(10, 12)H), 7.32 (d, *J* = 8 Hz, 2H, C(9, 13)H).

^{13}C-{^{1}H} NMR (500MHz, $CDCl_3$): δ = 18.04 (C(6)), 21.17 (C(20, 21)), 39.17 (C(2)), 58.54 (C(4)), 60.45 (C(7)), 61.33 (C(1)), 120.19 (C(5)), 127.86 (C(14, 18)), 128,72 (C(9, 13)), 129.03 (C(15, 17)), 129.07 (C(10, 12)), 135.38 (C(19)), 135.71 (C(16)), 136.54 (C(11)), 137.97 (C(8)).

MS (*m/z*, %): 291 (77) $[M]^+$, 276 (30), 186 (11), 143 (15), 105 (100).

Anal.calcd for $C_{21}H_{25}N$, (%): C, 86.55; H, 8.65; N, 4.81. Found, %: C, 86.37; H, 8.60; N, 4.79.

***(E)*-3-(iodo(trimethylsilyl)methylene)-4-(iodomethyl)-1-(4-methoxybenzyl)pyrrolidin-2-one (5a); Typical Procedure.**

To a solution of *N*-(4-methoxybenzyl)-*N*-(3-(trimethylsilyl)prop-2-yn-1-yl)prop-2-en-1-amine (754 mg, 2 mmol) and Et_2Zn (1 M in hexanes, 5 mL, 5 mmol) in CH_2Cl_2 (6 mL) was added Ti(O-*i*Pr)$_4$ (0.5 M in hexanes, 0.6 mL, 0.3 mmol). Ethylmagnesiurn bromide (2.5 M in Et_2O, 0.16 mL, 0.4 mmol) was then added and the reaction mixture rapidly turned black. After 18 h at 23 °C, the reaction mixture was cooled to -78 °C, and a solution of I_2 (1575 mg, 12,5 mmol) in THF (12,5 mL) was added via cannula. The reaction mixture was warmed to 23 °C, and stirred overnight. The mixture was then

partitioned between 25% aqueous KOH and ether. The organic layer was washed with water and aqueous $Na_2S_2O_3$, drying over $MgSO_4$. Evaporation of solvent and purification of the residue by column chromatography (diethyl ether : isopropyl alcohol : hexane = 1 : 1 :8) to afford **5a** (617 mg, 57 %). R_f 0.85.

^{1}H NMR (500MHz, $CDCl_3$): δ = 0.40 (s, 9H, C(14, 15, 16)H_3), 3.17 (m, 1H(A), C(6)IH_2), 3.18 (m, 1H(A), C(1)H_2), 3.27 (m, 1H, C(2)H), 3.45 (m, 1H(B), C(1)H_2), 3.55 (dd, *J* = 10 Hz, *J* = 3 Hz, 1H(B), C(6)IH_2), 3.83 (s, 3H, C(17)H_3), 4.27 (d, *J* = 14 Hz, 1H(A), C(7)H_2), 4.62 (d, *J* = 14 Hz, 1H(B), C(7)H_2), 6.89 (d, *J* = 9 Hz, 2H, C(10, 12)H, 7.20 (d, *J* = 9 Hz, 2H, C(9, 13)H).

^{13}C-{^{1}H} NMR (500MHz, $CDCl_3$): δ = 2.21 (C(14, 15, 16)), 7.77 (C(6)), 47.03 (C(7)), 48.98 (C(2)), 49.22 (C(1)), 55.31(C(17)), 114.20 (C(10, 12)), 127.77 (C(8)), 129.72 (C(9, 13)), 153.11 (C(3)), 159.31 (C(11)), 162.60 (C(4)).

Anal.calcd for $C_{17}H_{23}I_2NO_2Si$, (%): C, 36.77; H, 4.18; N, 2.52. Found, %: C, 36.21; H, 4.42; N, 2.39.

(*E*)-3-(iodo(trimethylsilyl)methylene)-4-(iodomethyl)-1-(4-methylbenzyl)pyrrolidin-2-one (5h)

Using the procedure described above *N*-(4-methylbenzyl)-*N*-(3-(trimethylsilyl)prop-2-yn-1-yl)prop-2-en-1-amine (542 mg, 2 mmol) gave crude product that was purified by flash chromatography (diethyl ether : isopropyl alcohol : hexane = 1 : 1 :8) to afford **5h** (641 mg, 61 %). R_f 0.87.

^{1}H NMR (500MHz, $CDCl_3$): δ = 0.41 (s, 9H, C(14, 15, 16)H_3), 2.36 (s, 3H, C(17)H_3), 3.17 (m, 1H(A), C(6)IH_2), 3.19 (m, 1H(A), C(1)H_2), 3.27 (m, 1H, C(2)H), 3.46 (m, 1H(B), C(1)H_2), 3.56 (dd, *J* = 10 Hz, *J* = 3 Hz, 1H(B), C(6)IH_2), 4.28 (d, *J* = 14 Hz, 1H(A), C(7)H_2), 4.66 (d, *J* = 14 Hz, 1H(B), C(7)H_2), 7.17 (s, 4H, C(9, 10, 12, 13)H).

^{13}C-{^{1}H} NMR (500MHz, $CDCl_3$): δ = 2.22 (C(14, 15, 16)), 7.78 (C(6)), 21.17 (C(17)), 47.37 (C(7)), 49.09 (C(2)), 49.29 (C(1)), 125.63 (C(5)), 128.36 (C(9,13)), 129.52 (C(10,12)), 132.63 (C(8)), 137.64 (C(11)), 153.08 (C(3)), 162.66 (C(4)).

MS (*m/z*, %): 539 (4) $[M]^+$, 420 (8), 396 (8), 292 (8), 105 (100), 79 (15).

Anal.calcd for $C_{17}H_{23}I_2NOSi$, (%): C, 37.86; H, 4.30; N, 2.60. Found, %: C, 38.08; H, 4.27; N, 2.44.

Preparation of bis-3-methyl-4-methylenepyrrolidines 7 and 9 via Ti-Mg-catalyzed carbozincation of bis-*N*-allyl substituted propargylamines with Et_2Zn in CH_2Cl_2.

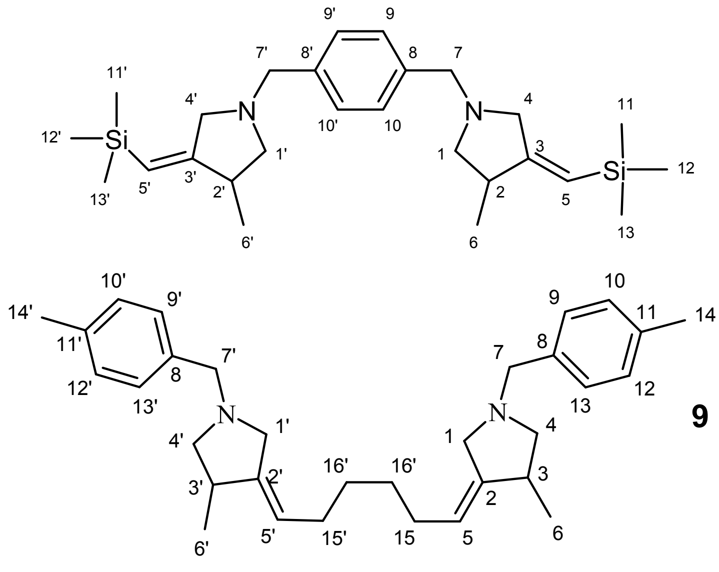

7

9

Figure 2 The numbering of atoms in the ^{13}C- and ^{1}H-NMR spectra of the compounds **7** and **9.**

1,4-bis(((*Z*)-3-methyl-4-((trimethylsilyl)methylene)pyrrolidin-1-yl)methyl)benzene (7); Typical Procedure.

To a solution of *N,N'*-(1,4-phenylenebis(methylene))bis(N-(3-(trimethylsilyl)prop-2-yn-1-yl)prop-2-en-1-amine) (874 mg, 2 mmol) and Et_2Zn (1 M in hexanes, 5 mL, 10 mmol) in CH_2Cl_2 (6 mL) was added Ti(O-*i*Pr)$_4$ (0.5 M in hexanes, 1.2 mL, 0.6 mmol). Ethylmagnesiurn bromide (2.5 M in Et_2O, 0.32 mL, 0.8 mmol) was then added and the reaction mixture rapidly turned black. After 18 h at 23 ^oC, the reaction mixture was diluted with Et_2O (5 mL), and 25 wt% KOH solution (3 mL) was added dropwise while the reaction flask was cooled in an ice bath. The aqueous layer was extracted with diethyl ether (3×5 mL). The combined organic layers were washed with brine (10 mL), dried over anhydrous $CaCl_2$. The reaction mixture was filtered through a filter paper and concentrated in vacuo to give crude product as a yellow oil. Evaporation of solvent and purification of the residue by column chromatography (diethyl ether : isopropyl alcohol : hexane = 1 : 1 :8) gave **7** (776 mg, 88 %) as colorless oil. R_f 0.52.

^{1}H NMR (500MHz, $CDCl_3$): δ = 0.08 (s, 18H, C(11, 12, 13, 11’, 12’, 13’)H_3), 1.09 (d, *J* = 7 Hz, 6H, C(6, 6’)H_3), 2.01 (t, *J* = 9 Hz, 2H(A), C(1, 1’)H_2), 2.67 (q, *J* = 7 Hz, 2H, C(2, 2’)H), 2.98 (t, *J* = 8 Hz, 2H(B), C(1, 1’)H_2), 3.03 (d, *J* = 14 Hz, 2H(A), C(4, 4’)H_2), 3.55 (d, *J* = 14 Hz, 2H(B), C(4, 4’)H_2), 3.60 (d, *J* = 13 Hz, 2H(A), C(7, 7’)H_2), 3.66 (d, *J* = 13 Hz, 2H(B), C(7, 7’)H_2), 5.30 (s, 2H, C(5, 5’)H), 7.31 (s, 4H, C(9, 10, 9’, 10’)H).

^{13}C-{^{1}H} NMR (500MHz, $CDCl_3$): δ = -0.43 (C(11, 12, 13, 11’, 12’, 13’)), 17.36 (C(6, 6’)), 40.32 (2, 2’), 59.42 (C(4, 4’)), 60.55 (C(7, 7’)), 61.31 (C(1, 1’)), 116.69 (C(5, 5’)), 128.78 (C(9, 10, 9’, 10’)), 137.65 (C(8, 8’)), 162.77 (C(3, 3’)).

MS (EI): m/z, % = 441 (16) [M^+], 440 (39), 367 (100), 272 (66), 207 (44), 168 (34), 104 (85), 73 (67), 44 (47).

Anal. calcd for $C_{26}H_{44}N_2Si_2$, (%): C, 70.84; H, 10.06; N, 6.35. Found, %: C, 71.07; H, 9.95; N, 6.39.

(4*Z*,4'*Z*)-4,4'-(octane-2,7-diylidene)bis(3-ethyl-1-(4-methylbenzyl)pyrrolidine) (9)

Using the procedure described above *N¹,N¹⁰*-diallyl-*N¹,N¹⁰*-bis(4-methylbenzyl)deca-*2,8*-diyne-1,10-diamine (906 mg, 2 mmol) gave crude product that was purified by flash chromatography (diethyl ether : isopropyl alcohol : hexane = 1 : 1 :8) to afford **9** (872 mg, 85 %). R_f 0.80.

^{1}H NMR (500MHz, $CDCl_3$): δ = 1.10 (d, *J* = 7 Hz, 6H, C(6, 6')H_3), 1.36 (s, 4H, C(16, 16')H_2), 1.92 (d, *J* = 5Hz, 4H, C(15, 15')H_2), 2.04 (t, *J* = 9 Hz, 2H(A), C(4, 4')H_2), 2.38 (s, 6H, C(14, 14'), 2.70 (q, *J* = 7 Hz, 2H, C(3, 3')H), 2.97 (d, *J* = 14 Hz, 2H(A), C(1, 1')H_2), 3.00 (t, *J* = 8 Hz, 2H(B), C(4, 4')H_2), 3.48 (d, *J* = 14 Hz, 2H(B), C(1, 1')H_2), 3.61 (d, *J* = 13 Hz, 2H(A), C(7, 7')H_2), 3.65 (d, *J* = 13 Hz, 2H(B), C(7, 7')H_2), 5.15 (m, 2H, C(5,5')H), 7.17 (d, *J* = 8 Hz, 4H, C(10, 12, 10',12')H), 7.28 (d, *J* = 8 Hz, 4H, C(9, 13, 9', 13')H).

^{13}C-{^{1}H} NMR (500MHz, $CDCl_3$): δ = 17.75 (C(6, 6')), 21.15 (C(14, 14')), 29.24 (C(16, 16')), 29.37 (C(15, 15')), 37.33 (C(3, 3')), 56.82 (C(1, 1')), 60.61 (C(7, 7')), 62.28 (C(4, 4')), 119.69 (C(5, 5')), 128,77 (C(9, 13, 9', 13')), 128.94 (C(10, 12, 10', 12')), 136.07 (C(8, 8')), 136.46 (C(11, 11')), 144.09 (C(2, 2')).

MS (*m/z*, %): 457 (3) $[M]^+$, 456 (3), 351 (1), 200 (10), 105 (100), 79 (6).

Anal.calcd for $C_{32}H_{44}N_2$, (%): C, 84.16; H, 9.71; N, 6.13. Found, %: C, 83.89; H, 9.50; N, 6.17.

Preparation of 3-methyl-4-methylenepyrrolidines 11a-c, 12a and 14 via Ti-Mg-catalyzed carbozincation of allyl substituted but-2-yne-1,4-diamines with Et_2Zn in CH_2Cl_2.

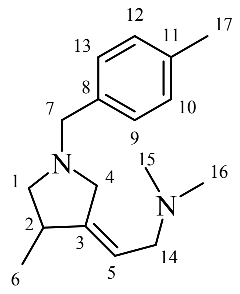

11a

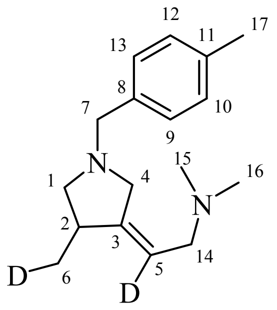

12a

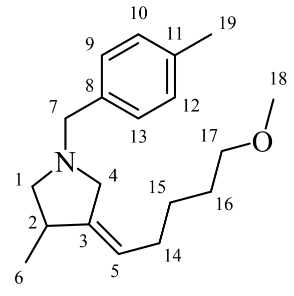

14

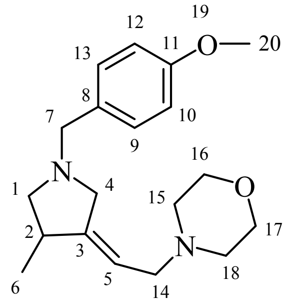

11b

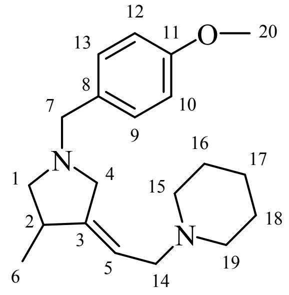

11c

Figure 3 The numbering of atoms in the ^{13}C- and ^{1}H-NMR spectra of the compounds **11a-c**, **12a** and **14**.

(*Z*)-*N*,*N*-dimethyl-2-(4-methyl-1-(4-methylbenzyl)pyrrolidin-3-ylidene)ethan-1-amine (11a)

To a solution of N^1-allyl-N^4,N^4-dimethyl-N^1-(4-methylbenzyl)but-2-yne-1,4-diamine (512 mg, 2 mmol) and Et_2Zn (1 M in hexanes, 5 mL, 5 mmol) in CH_2Cl_2 (6 mL) was added Ti(O-*i*Pr)$_4$ (0.5 M in hexanes, 0.6 mL, 0.3 mmol). Ethylmagnesiurn bromide (2.5 M in Et_2O, 0.16 mL, 0.4 mmol) was then added and the reaction mixture rapidly turned black. After 18 h at 23 ^{o}C, the reaction mixture was diluted with Et_2O (5 mL), and 25 wt% KOH solution (3 mL) was added dropwise while the reaction flask was cooled in an ice bath. The aqueous layer was extracted with diethyl ether (3×5 mL). The combined organic layers were washed with brine (10 mL), dried over anhydrous $CaCl_2$. The reaction mixture was filtered through a filter paper and concentrated in vacuo to give crude product as a yellow oil. Evaporation of solvent and purification of the residue by column chromatography (diethyl ether : isopropyl alcohol : hexane = 1 : 1 :8) gave **11a** (464 mg, 90 %) as colorless oil. R_f 0.47.

^{1}H NMR (500MHz, $CDCl_3$): δ = 1.12 (d, J = 7 Hz, 3H, C(6)H$_3$), 2.07 (t, J = 9 Hz, 1H(A), C(1)H$_2$), 2.35 (s, 6H, C(15, 16)H$_3$), 2.36 (s, 3H, C(17)H$_3$), 2.75 (q, J = 7 Hz, 1H, C(2)H), 2.99 (m, 1H(A), C(4)H$_2$), 3.00 (m, 2H, C(14)H$_2$), 3.02 (m, 1H(B), C(1)H$_2$), 3.50 (d, J = 14 Hz, 1H(B), C(4)H$_2$), 3.60 (d, J = 13 Hz, 1H(A), C(7)H$_2$),

3.65 (d, J = 12 Hz, 1H(B), C(7)H_2), 5.31 (m, 1H, C(5)H), 7.15 (d, J = 8 Hz, 2H, C(10, 12)H), 7.24 (d, J = 8Hz, 2H, C(9, 13)H).

^{13}C-{^{1}H} NMR (500MHz, $CDCl_3$): δ = 17.49 (C(6)), 21.21 (C(17)), 37.69 (C(2)), 44.87 (C(15, 16)), 56.55 (C(4)), 57.66 (C(14)), 60.25 (C(7)), 61.64 (C(1)), 114.34 (C(5)), 128.77 (C(9, 13)), 129.02 (C(10, 12)), 135.36 (C(8)), 136.73 (C(11)), 149.76 (C(3)).

MS (EI): m/z, % = 258 (<1) [M^+], 257 (<1), 213 (80), 198 (57), 105 (100).

Anal. calcd for $C_{17}H_{26}N_2$, (%): C, 79.02; H, 10.14; N, 10.84. Found, %: C, 78.86; H, 10.09; N, 11.0.

(*Z*)-4-(2-(1-(4-methoxybenzyl)-4-methylpyrrolidin-3-ylidene)ethyl)morpholine (11b)

Using the procedure described above *N*-allyl-*N*-(4-methylbenzyl)-4-morpholinobut-2-yn-1-amine (596 mg, 2 mmol) gave crude product that was purified by column chromatography (diethyl ether : isopropyl alcohol : hexane = 1 : 1 :8) to afford **11b** (534 mg, 89 %). R_f 0.48.

^{1}H NMR (500MHz, $CDCl_3$): δ = 1.09 (d, J = 7 Hz, 3H, C(6)H_3), 2.01 (t, J = 8 Hz, 1H(A), C(1)H_2), 2.42 (s, 4H, C(15, 18)H_2), 2.71 (q, J = 8 Hz, 1H, C(2)H), 2.88 (d, J = 6 Hz, 2H, C(14)H_2), 2.93 (d, J = 14 Hz, 1H(A), C(4)H_2), 2.97 (t, J = 8 Hz, 1H(B), C(1)H_2), 3.47 (d, J = 14 Hz, 1H(B), C(4)H_2), 3.55 (d, J = 13 Hz, 1H(A), C(7)H_2), 3.59 (d, J = 13 Hz, 1H(B), C(7)H_2), 3.71 (s, 4H, C(16, 17)H_2), 3.80 (s, 3H, C(19)H_3), 5.25 (s, 1H, C(5)H), 6.87 (d, J = 8 Hz, 2H, C(10, 12)H), 7.25 (d, J = 8 Hz, 2H, C(9, 13)H).

^{13}C-{^{1}H} NMR (500MHz, CDl_3): δ = 17.53 (C(6)), 37.66 (C(2)), 53.61 (C(15, 18)), 55.21 (C(19)), 56.74 (C(4)), 57.82 (C(14), 60.04 (C(7)), 61.78 (C(1)), 66.99 (C(16, 17)), 113.63 (C(10, 12)), 115.49 (C(5)), 129.89 (C(9, 13)), 130.93 (C(8)), 148.32 (C(3)), 158.66 (C(11)).

MS (EI): m/z, % = 316 (<1) [M^+], 229 (39), 121 (100), 77 (4).

Anal.calcd for $C_{19}H_{28}N_2O_2$, (%): C, 72.12; H, 8.92; N, 8.85. Found, %: C, 72.15; H, 8.79; N, 8.49.

(*Z*)-1-(2-(1-(4-methoxybenzyl)-4-methylpyrrolidin-3-ylidene)ethyl)piperidine (11c)

Using the procedure described above *N*-allyl-*N*-(4-methoxybenzyl)-4-(piperidin-1-yl)but-2-yn-1-amine (624 mg, 2 mmol) gave crude product that was purified by column chromatography (diethyl ether : isopropyl alcohol : hexane = 1 : 1 :8) to afford **11c** (515 mg, 82 %). R_f 0.79.

^{1}H NMR (500MHz, $CDCl_3$): δ = 1.04 (d, J = 7 Hz, 3H, C(6)H_3), 1.44 (s, 2H, C(17)H_2), 1.59 (p, J = 6 Hz, 4H, (C(16, 18)), 1.99 (t, J = 9 Hz, 1H(A), C(1)H_2), 2.36 (s, 4H, C(15, 19)H_2), 2.71 (q, J = 8 Hz, 1H, C(2)H), 2.84 (d, J = 7 Hz, 2H, C(14)H_2), 2.93 (d, J = 14 Hz, 1H(A), C(4)H_2), 2.97 (t, J = 8 Hz, 1H(B), C(1)H_2), 3.47 (d, J = 14Hz, 1H(B), C(4)H_2), 3.55 (d, J = 13 Hz, 1H(A), C(7)H_2), 3.59 (d, J

= 13Hz, 1H(B), C(7)H_2), 3.81 (s, 3H, C(20)H_3), 5.29 (m, 1H, C(5)H), 6.87 (d, J = 8 Hz, 2H, C(10, 12)H), 7.26 (d, J = 8 Hz, 2H, C(9, 13)H).

^{13}C-{^1H} NMR (500MHz, $CDCl_3$): δ = 17.49 (C(6)), 24.39 (C(17)), 25.95 (C(16, 18)), 37.62 (C(2)), 54.52 (C(15, 19)), 55.23 (C(20)), 56.77 (C(4)), 58.24 (C(14)), 60.10 (C(7)), 61.86 (C(1)), 113.61 (C(10, 12)), 116.53 (C(5)), 129.91 (C(9, 13)), 131.05 (C(8)), 147.19 (C(3)), 158.63 (C(11)).

MS (EI): m/z, % = 314 (<1) [M^+], 121 (100), 77 (5).

Anal.calcd for $C_{20}H_{30}N_2O$, (%): C, 76.39; H, 9.62; N, 8.91. Found, %: C, 76.44; H, 9.86; N, 8.59.

(*Z*)-*N*,*N*-dimethyl-2-(4-(methyl-*d*)-1-(4-methylbenzyl)pyrrolidin-3-ylidene)ethan-1-amine-2-*d* (12a)

Using the procedure described above N^1-allyl-N^4,N^4-dimethyl-N^1-(4-methylbenzyl)but-2-yne-1,4-diamine (512 mg, 2 mmol) and D_2O (instead of H_2O)gave crude product that was purified by column chromatography (diethyl ether : isopropyl alcohol : hexane = 1 : 1 :8) to afford **12a** (480 mg, 88 %). R_f 0.85.

^1H NMR (500MHz, $CDCl_3$): δ = 1.10 (m, 2H, C(6)DH_2), 2.04 (t, J = 8 Hz, 1H(A), C(1)H_2), 2.23 (s, 6H, C(15,16)H_3), 2.37 (s, 3H, C(17)H_3), 2.73 (p, J = 7 Hz, 1H, C(2)H), 2.83 (s, 2H, C(14)H_2), 2.96 (d, J = 14 Hz, 1H(A), C(4)H_2), 2.99 (t, J = 8 Hz, 1H(B), C(1)H_2), 3.49 (d, J = 14 Hz, 1H(B), C(4)H_2), 3.59 (d, J = 13 Hz, 1H(A), C(7)H_2), 3.64 (d, J = 13Hz,, 1H(B), C(7)H_2), 7.15 (d, J = 8 Hz, 2H, C(10, 12)H), 7.25 (d, J = 8 Hz, 2H, C(9, 13)H).

^{13}C-{^1H} NMR (500MHz, $CDCl_3$): δ = 17.39 (t, J = 19 Hz, C(6)), 21.12 (C(17)), 37.50 (C(2)), 45.11 (C(15, 16)), 56.69 (C(4)), 58.29 (C(14)), 60.45 (C(7)), 61.88(C(1)), 128.72 (C(9, 13)), 128.96 (C(10, 12)), 135.84 (C(8)), 136.55 (C(11)), 147.53 (C(3)).

MS (EI): m/z, % = 260 (<1) [M^+], 215 (36), 199 (30), 105 (100), 79 (7).

Anal.calcd for $C_{17}H_{24}D_2N_2$, (%): C, 78.41; N, 10.76. Found, %: C, 78.48; N, 11.08.

(*Z*)-3-(5-methoxypentylidene)-4-methyl-1-(4-methylbenzyl)pyrrolidine (14)

Using the procedure described above *N*-allyl-7-methoxy-*N*-(4-methylbenzyl)hept-2-yn-1-amine (570 mg, 2 mmol) and H_2O (instead of D_2O)gave crude product that was purified by column chromatography (diethyl ether : isopropyl alcohol : hexane = 1 : 1 :8) to afford **14** (517 mg, 90 %). R_f 0.63.

^1H NMR (500MHz, $CDCl_3$): δ = 1.08 (d, J = 7 Hz, 3H, C(6)H_3), 1.42 (p, J = 8 Hz, 2H, C(15)H_2), 1.58 (p, J = 8 Hz, 2H, C(16)H_2), 1.95 (qv, J = 7 Hz, 2H, C(14)H_2), 2.03 (t, J = 8 Hz, 1H(A), C(1)H_2), 2.37 (s, 3H, C(19)H_3), 2.96 (d, J = 13 Hz, 1H(A), C(4)H_2), 2.99 (t, J = 8 Hz, C(1)H_2), 3.34 (s, 3H, C(18)H_3), 3.37 (t, J = 7 Hz, 2H, C(17)H_2), 3.47 (d, J = 13 Hz, 1H(B), C(4)H_2), 3.59 (d, J = 13 Hz, 1H(A),

C(7)H_2), 3.64 (d, J = 13 Hz, 1H(B), C(7)H_2), 5.14 (m, 1H, C(5)H), 7.15 (d, *J* = 8 Hz, 2H, C(10, 12)H), 7.26 (d, *J* = 8 Hz, 2H, C(9, 13)H).

^{13}C-{^{1}H} NMR (500MHz, $CDCl_3$): δ = 17.68 (C(6)), 21.12 (C(19)), 26.08 (C(15)), 29.20 (C(14)), 29.25 (C(16)), 37.30 (C(2)), 56.73 (C(4)), 58.55 (C(18)), 60.54 (C(7)), 62.22 (C(1)), 119.43 (C(5)), 128.77 (C(9, 13)), 128.93 (C(10, 12)), 135.95 (C(8)), 136.47 (C(11)), 144.32 (C(3)).

MS (EI): m/z, % = 287 (18) [M^+], 200 (38), 105 (100), 79 (9).

Anal.calcd for $C_{19}H_{29}NO$, (%): C, 79.39; H, 10.17; N, 4.87. Found, %: C, 79.11; H, 10.00; N, 4.53.

This work was financially supported by the Russian Science Foundation (grant No. 19-73-10113).

References

1. Shao, Y.; Zhang, F.; Zhang, J.; Zhou, X. *Angew. Chem. Int. Ed.* **2016**, *55*, 11485.
2. Li, Hui-Jing; Guillot, Regis; Gandon, Vincent. *Journal of Organic Chemistry*, **2010**, *75*(24), 8435.
3. Shaibakova, M. G.; Titova, I. G.; Ibragimov, A. G.; Dzhemilev, U.M. *Russ. J. Org. Chem.* **2008**, *44*, 1126.

^{1}H-NMR spectrum of (*Z*)-1-(4-methoxybenzyl)-3-methyl-4-((trimethylsilyl)methylene)pyrrolidine (**3a**)

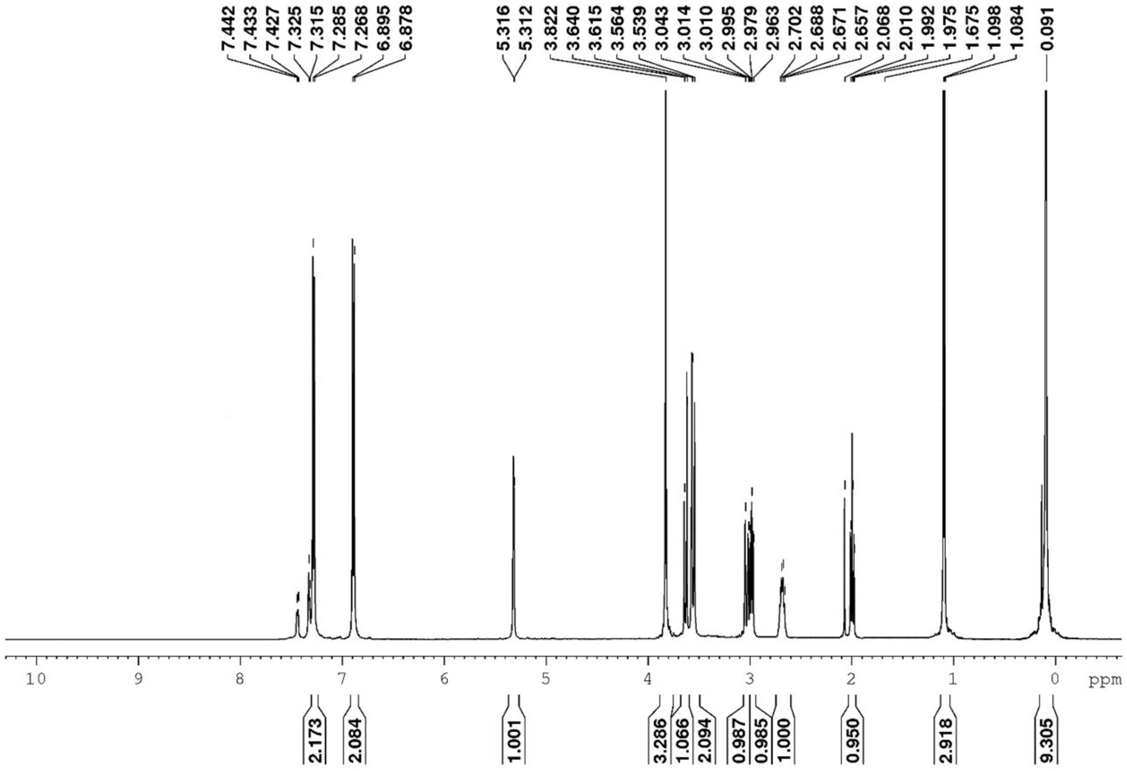

^{13}C-NMR spectrum of (*Z*)-1-(4-methoxybenzyl)-3-methyl-4-((trimethylsilyl)methylene)pyrrolidine (**3a**)

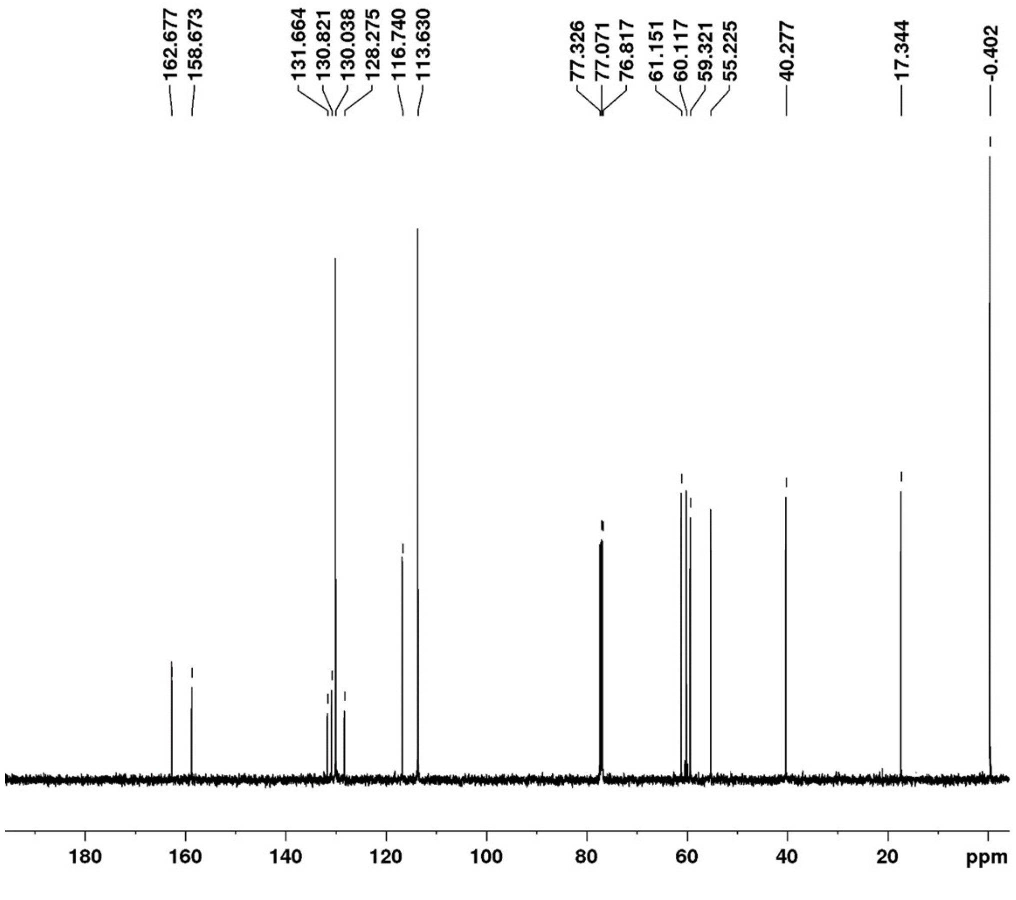

NOESY spectrum of (*Z*)-1-(4-methoxybenzyl)-3-methyl-4-((trimethylsilyl)methylene)pyrrolidine (**3a**)

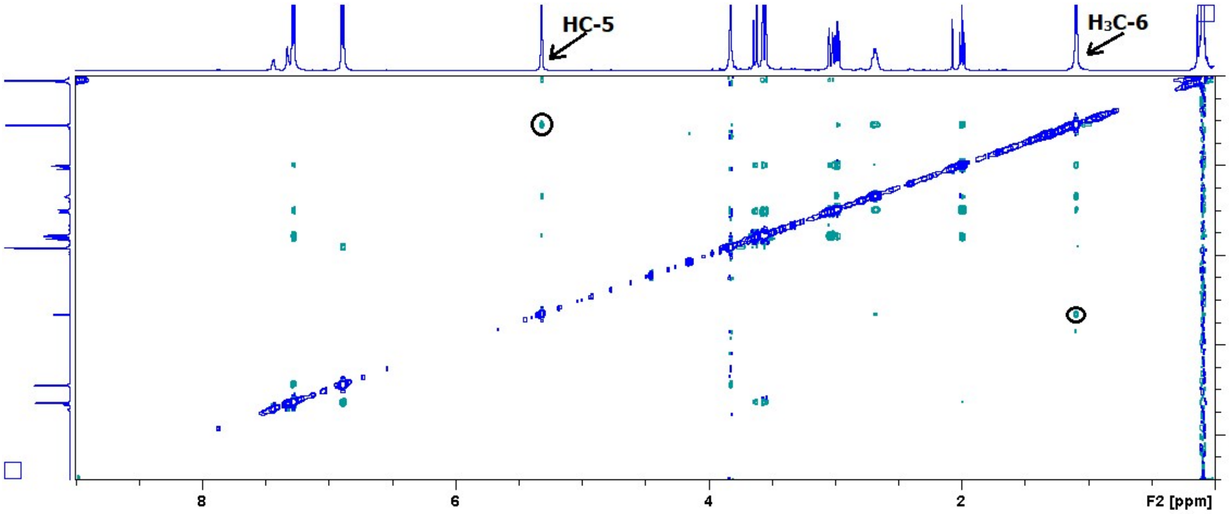

NOESY spectrum of (*Z*)-3-benzylidene-1-(4-chlorobenzyl)-4-methylpyrrolidine (**3b**)

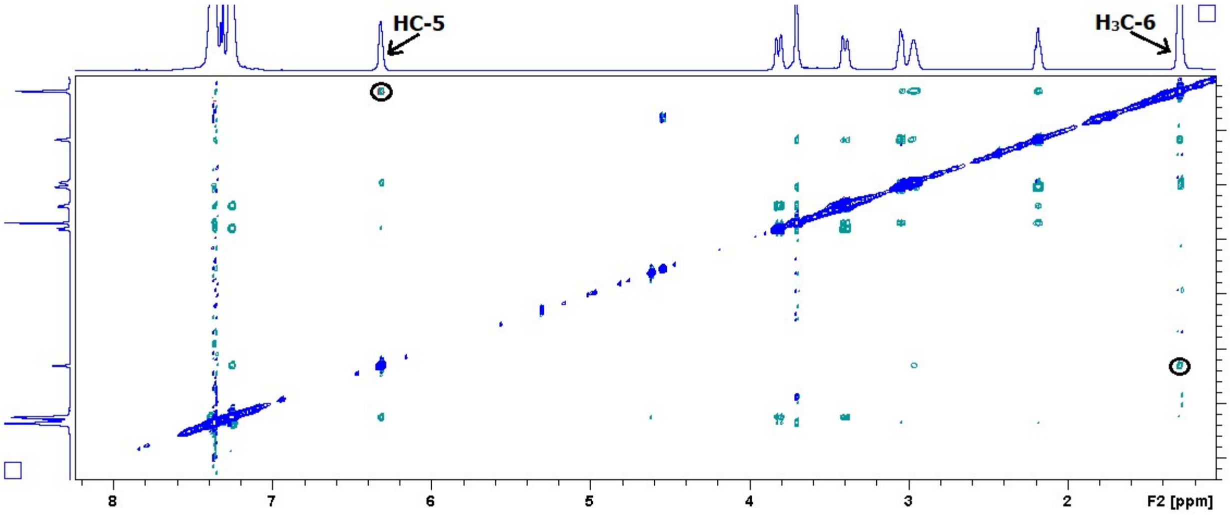

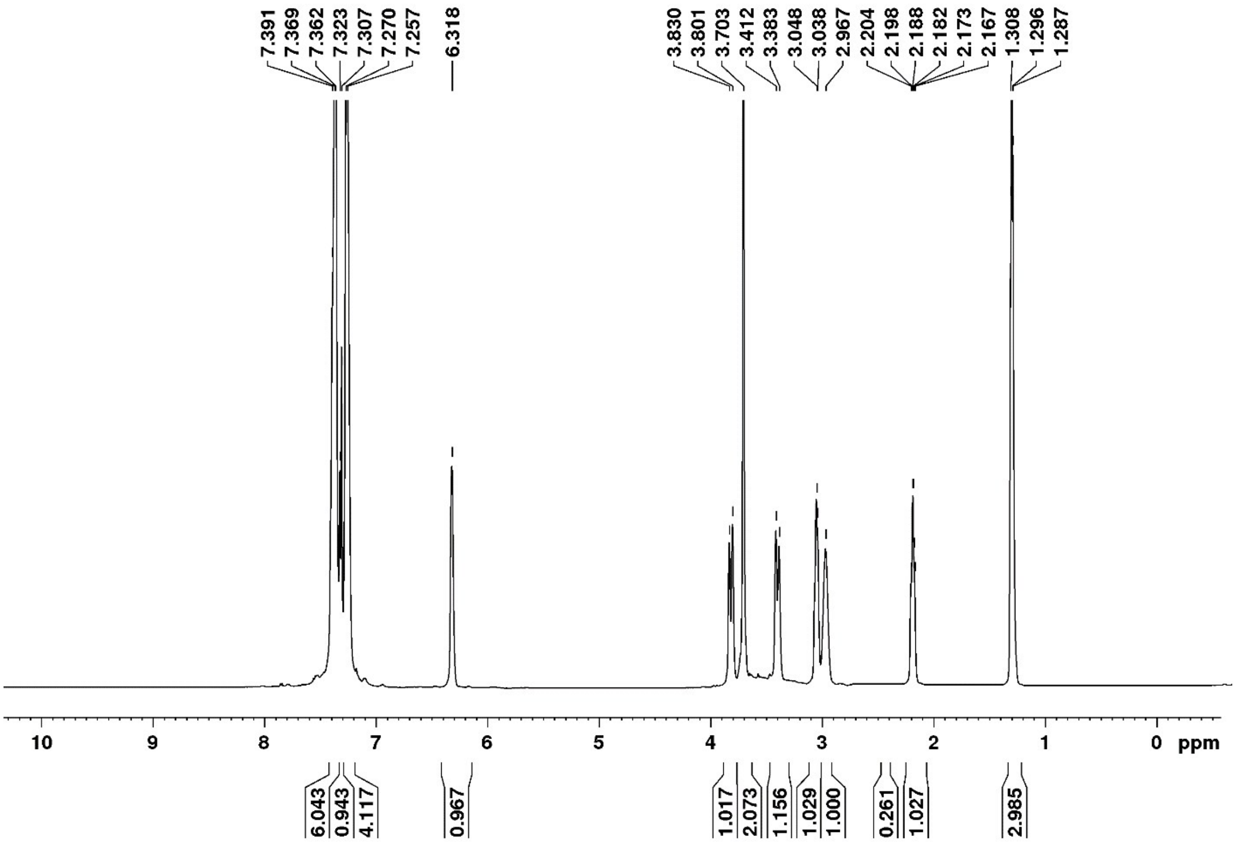

^{13}C-NMR spectrum of (*Z*)-3-benzylidene-1-(4-chlorobenzyl)-4-methylpyrrolidine (**3b**)

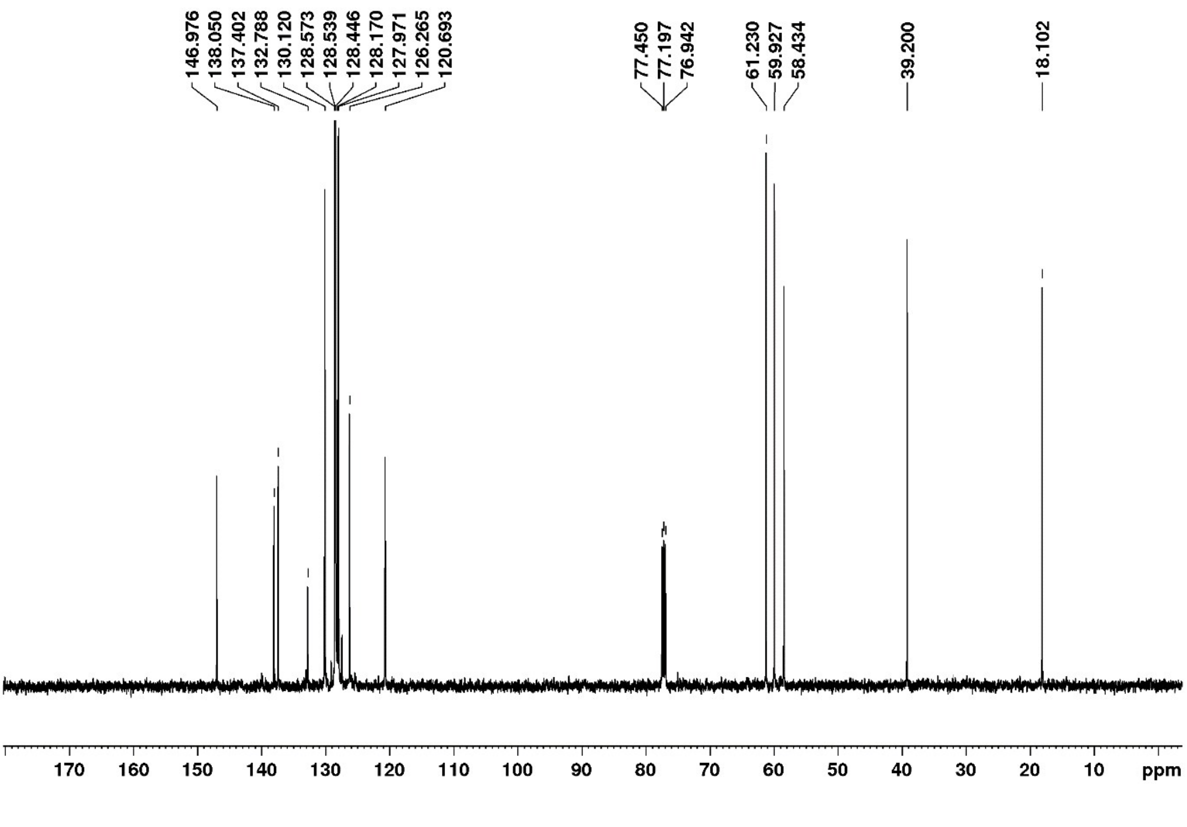

^{1}H-NMR spectrum of (*Z*)-3-benzylidene-4-methyl-1-(4-methylbenzyl)pyrrolidine (**3c**)

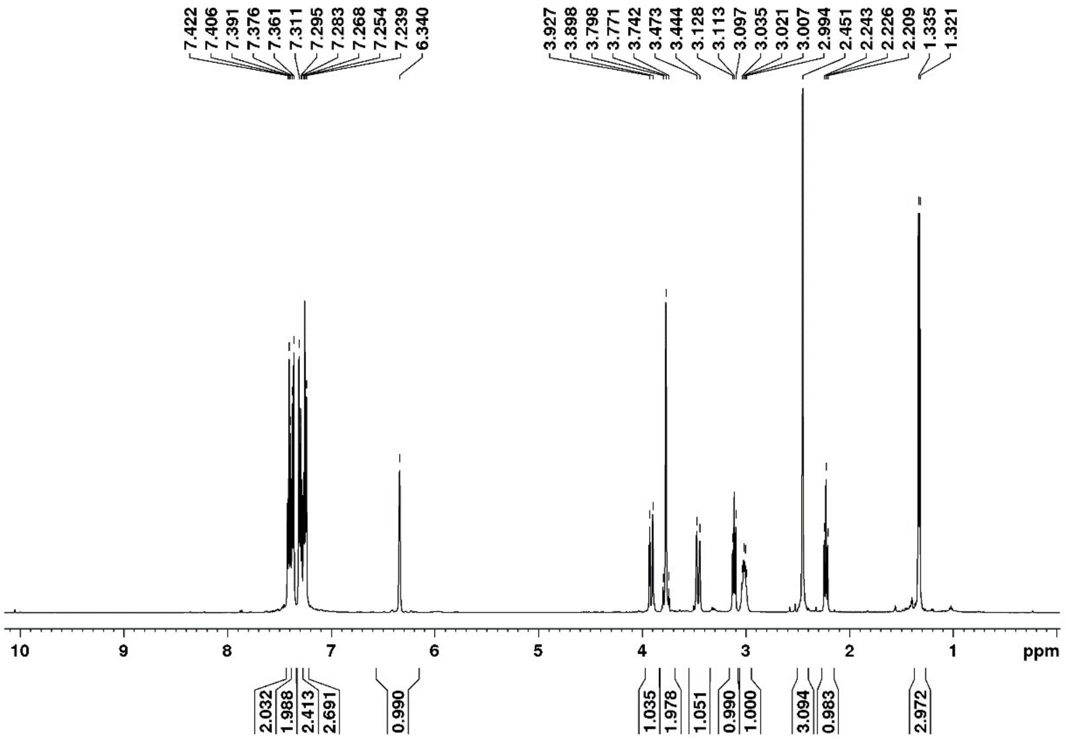

^{13}C-NMR spectrum of (*Z*)-3-benzylidene-4-methyl-1-(4-methylbenzyl)pyrrolidine (**3c**)

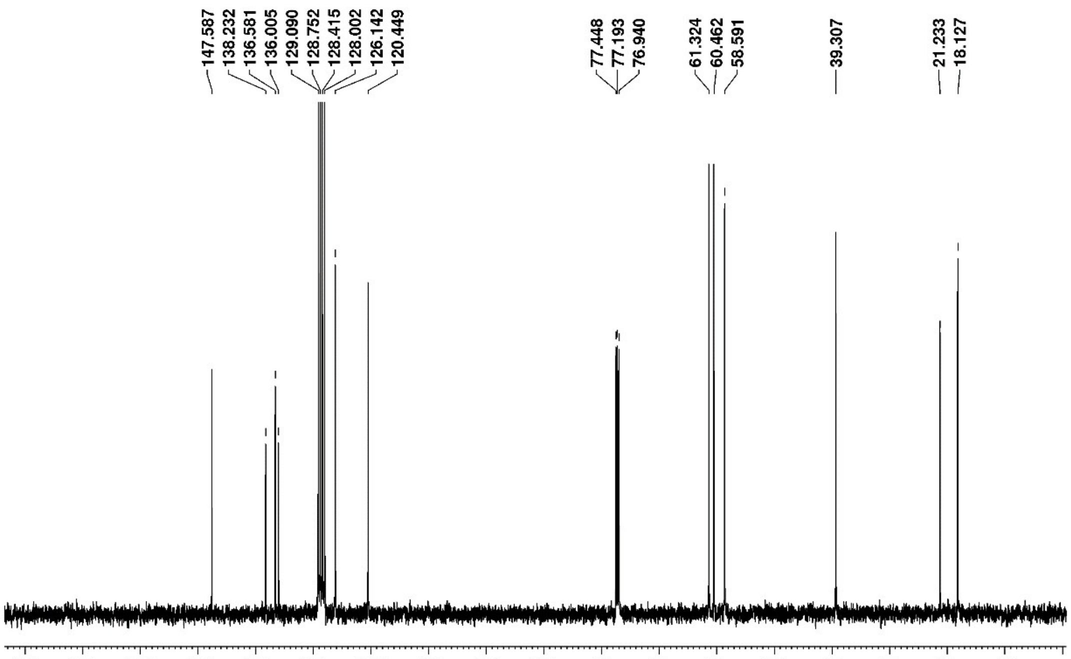

^{1}H-NMR spectrum of (*Z*)-3-methyl-1-(4-methylbenzyl)-4-pentylidenepyrrolidine (**3d**)

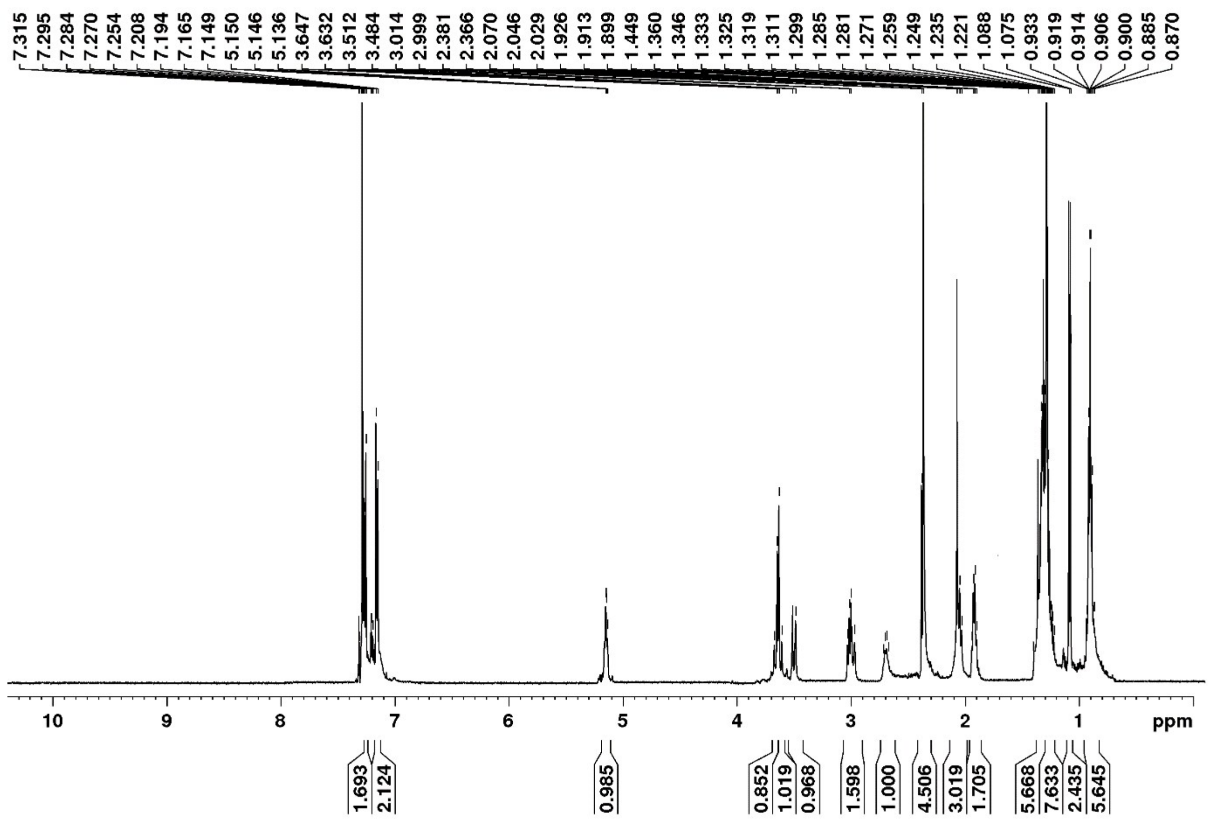

^{13}C-NMR spectrum of (*Z*)-3-methyl-1-(4-methylbenzyl)-4-pentylidenepyrrolidine (**3d**)

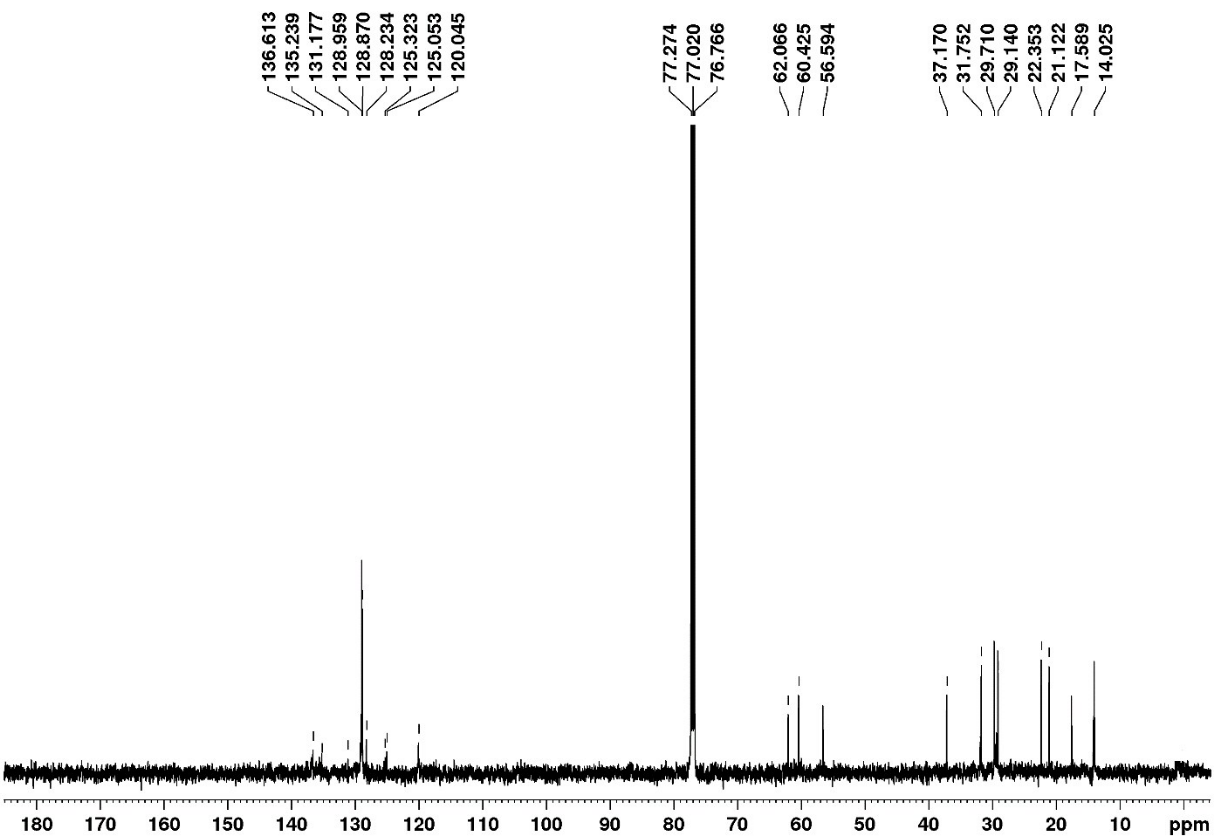

^{1}H-NMR spectrum of (*Z*)-1-(furan-2-ylmethyl)-3-methyl-4-((trimethylsilyl)methylene)pyrrolidine (**3e**)

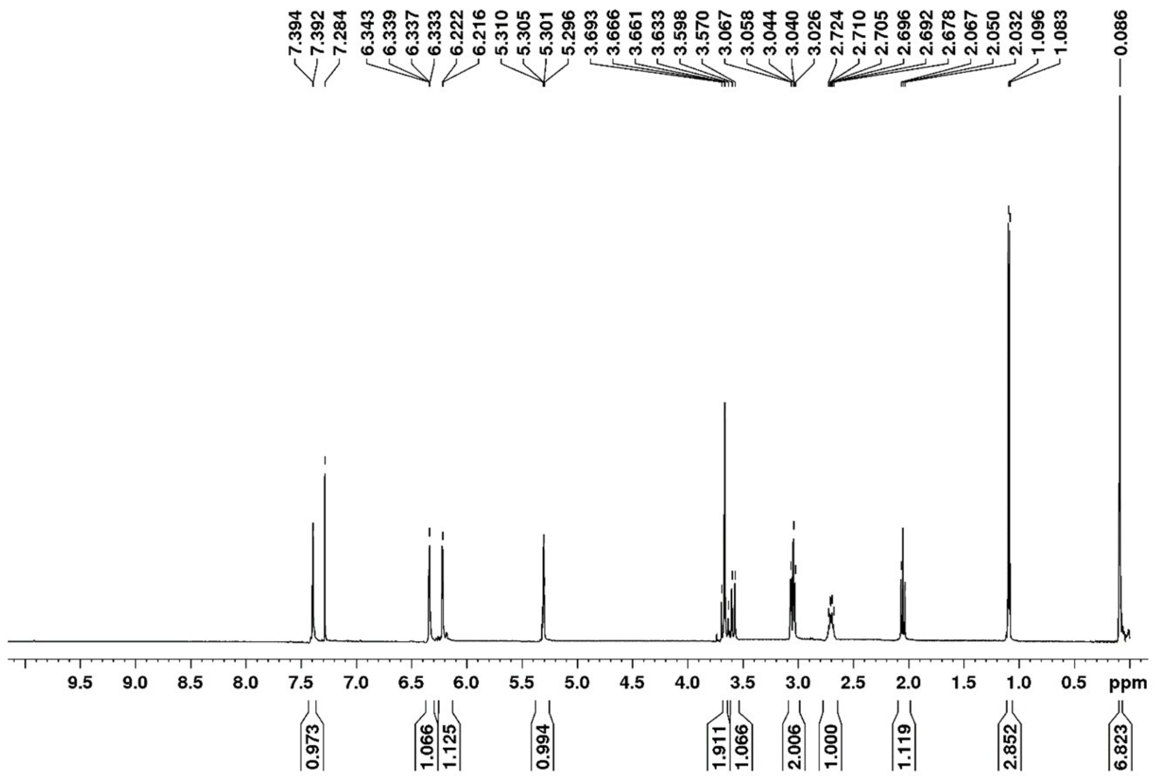

^{13}C-NMR spectrum of (Z)-1-(furan-2-ylmethyl)-3-methyl-4-((trimethylsilyl)methylene)pyrrolidine (**3e**)

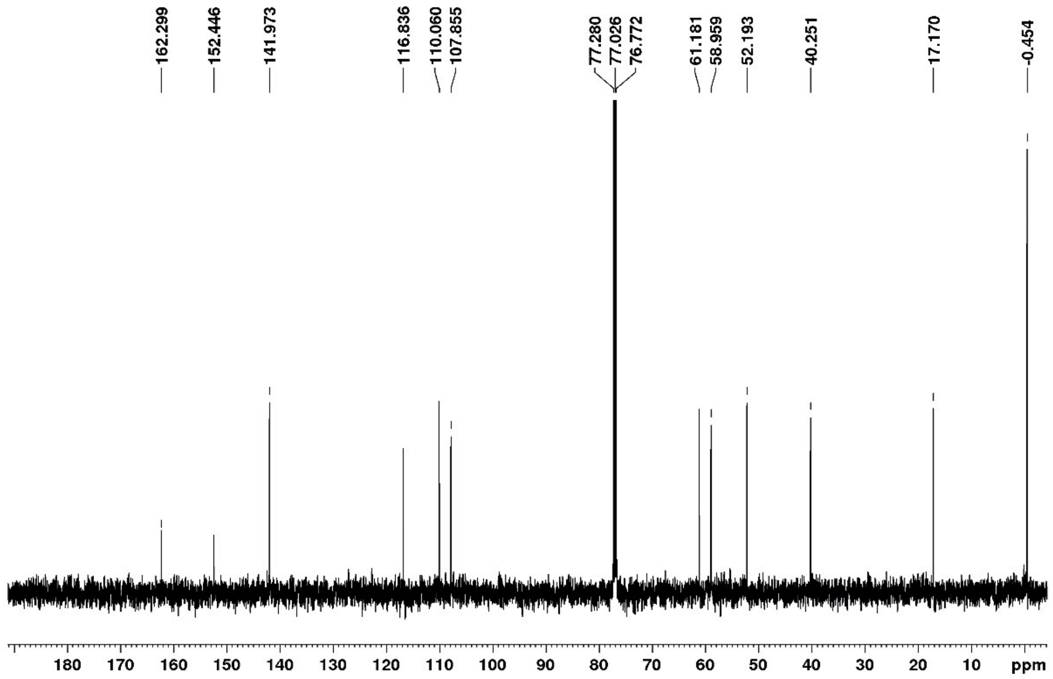

^{1}H-NMR spectrum of (*Z*)-3-methyl-1-(thiophen-2-ylmethyl)-4-((trimethylsilyl)methylene)pyrrolidine (**3f**)

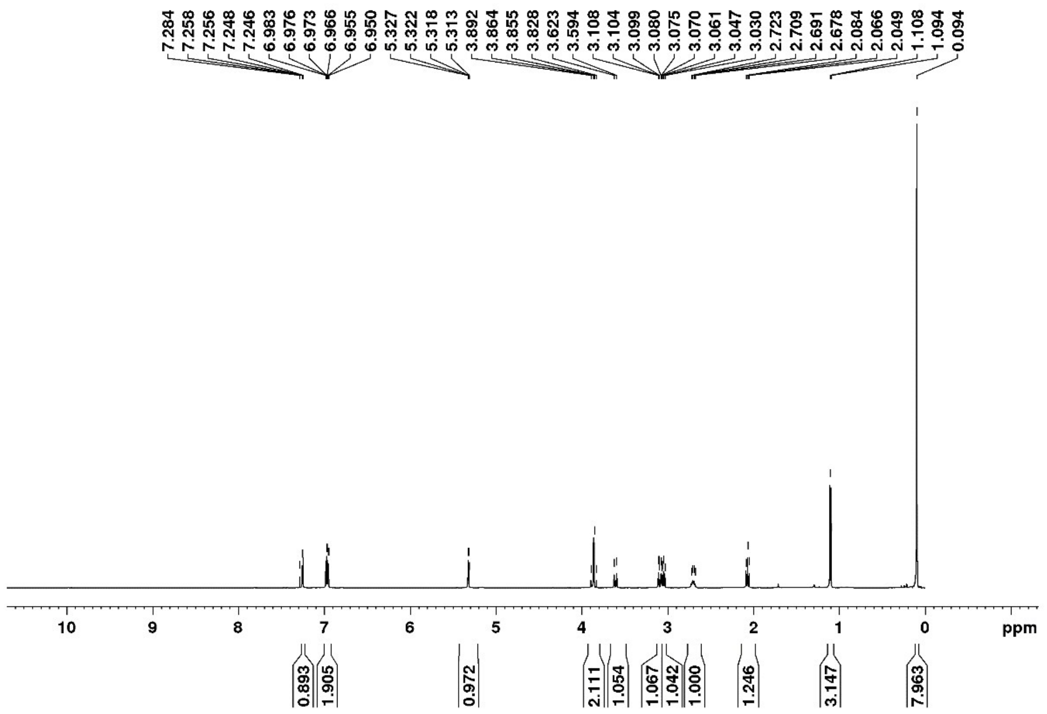

^{13}C-NMR spectrum of (*Z*)-3-methyl-1-(thiophen-2-ylmethyl)-4-((trimethylsilyl)methylene)pyrrolidine (**3f**)

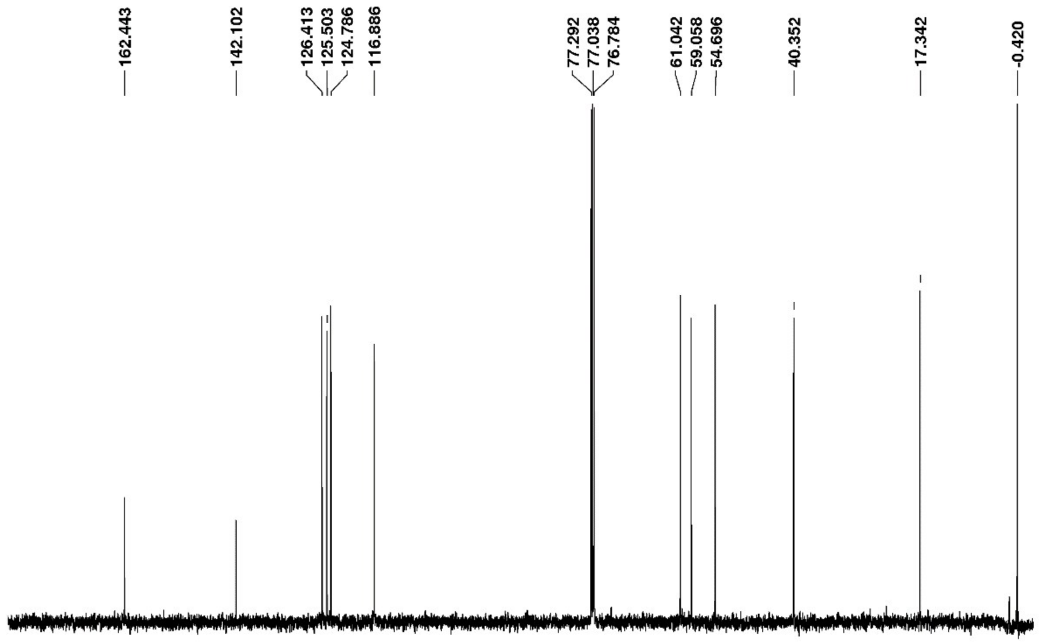

^{1}H-NMR spectrum of **(*Z*)-3-(methyl-*d*)-1-(thiophen-2-ylmethyl)-4-((trimethylsilyl)methylene-*d*)pyrrolidine (4f)**

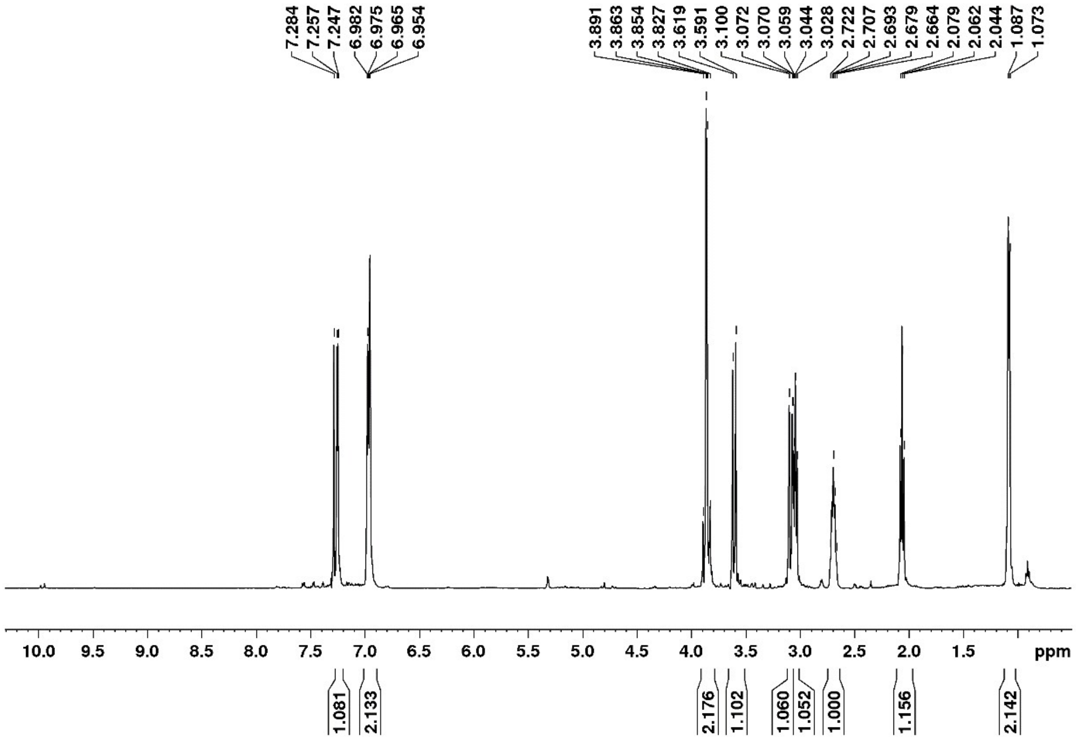

^{13}C-NMR spectrum of **(*Z*)-3-(methyl-*d*)-1-(thiophen-2-ylmethyl)-4-((trimethylsilyl)methylene-*d*)pyrrolidine (4f)**

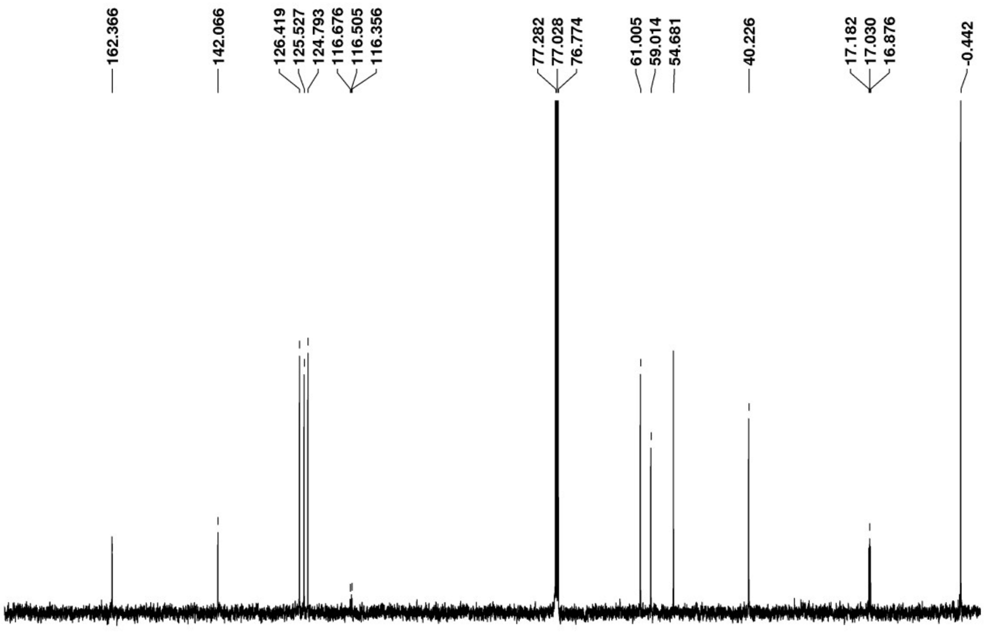

^{1}H-NMR spectrum of (*Z*)-1-(4-chlorobenzyl)-3-methyl-4-((trimethylsilyl)methylene)pyrrolidine (**3g**)

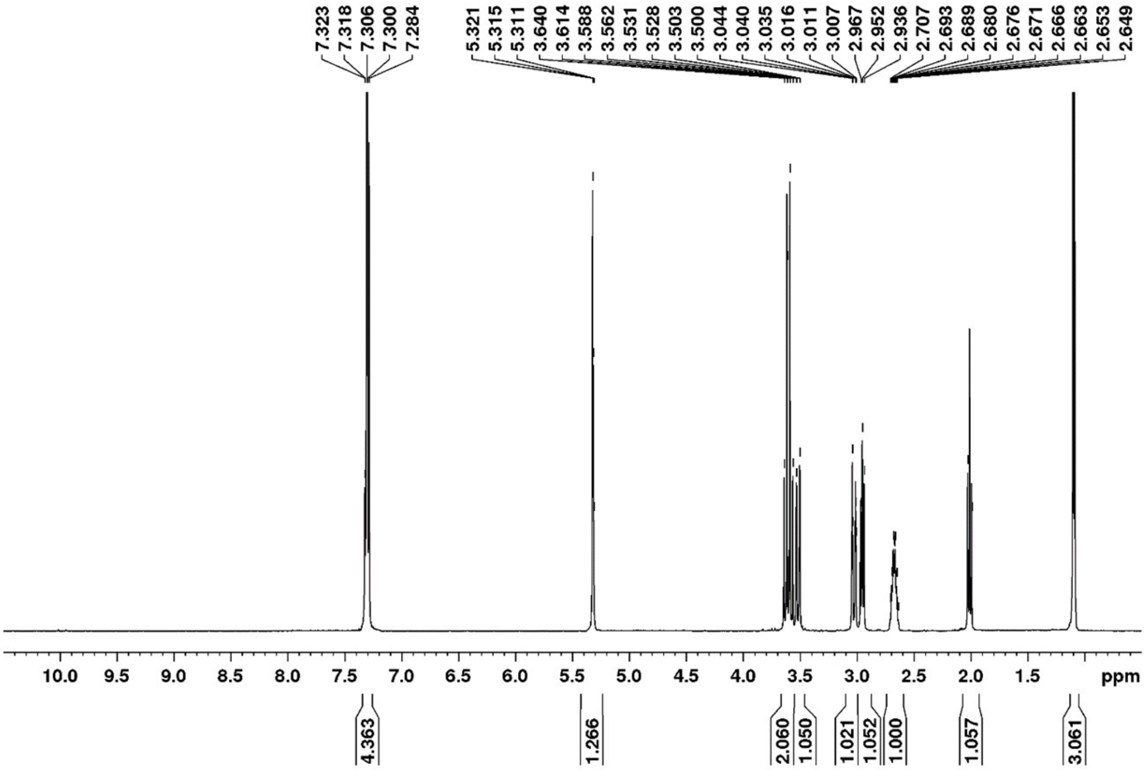

^{13}C-NMR spectrum of (*Z*)-1-(4-chlorobenzyl)-3-methyl-4-((trimethylsilyl)methylene)pyrrolidine (**3g**)

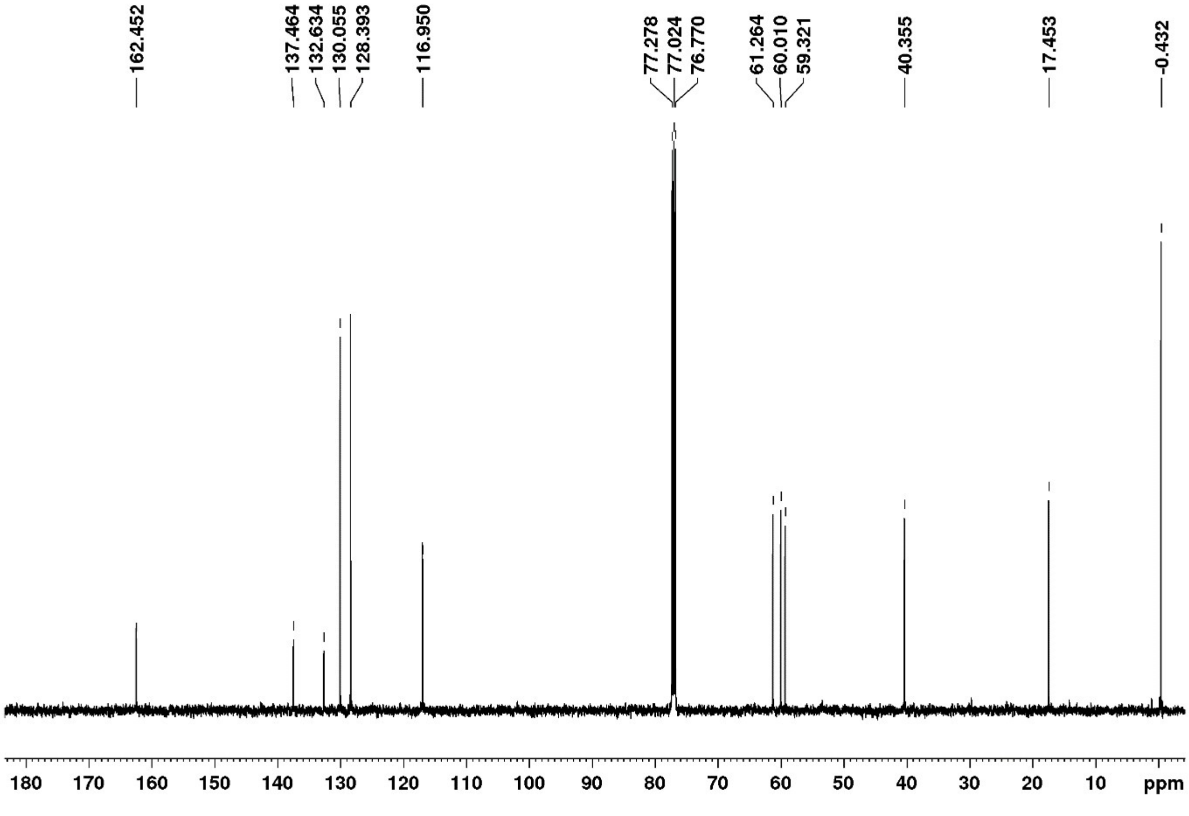

^{1}H-NMR spectrum of (*Z*)-3-(methyl-d)-1-(4-methylbenzyl)-4-((trimethylsilyl)methylene-d)pyrrolidine (**4h**)

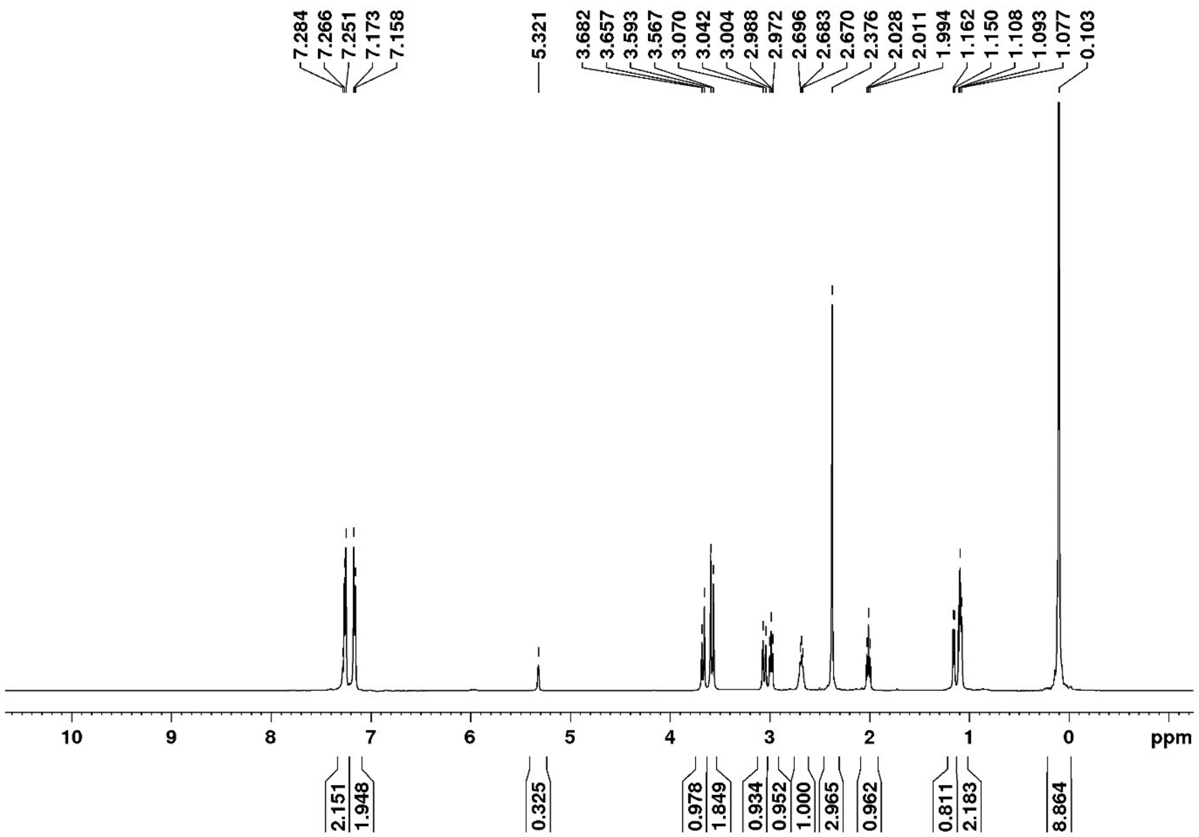

^{13}C-NMR spectrum of (*Z*)-3-(methyl-*d*)-1-(4-methylbenzyl)-4-((trimethylsilyl)methylene-d)pyrrolidine (**4h**)

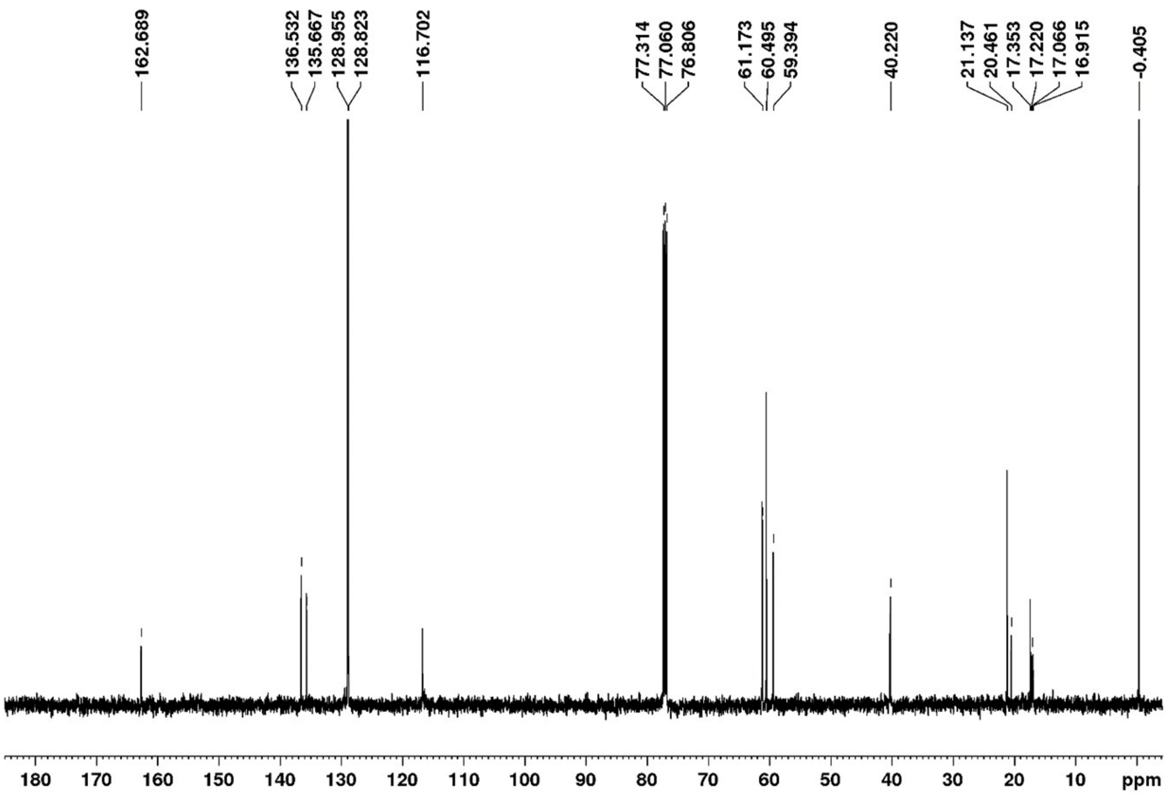

^{1}H-NMR spectrum of (*Z*)-3-methyl-1-(4-methylbenzyl)-4-(4-methylbenzylidene)pyrrolidine (**3i**)

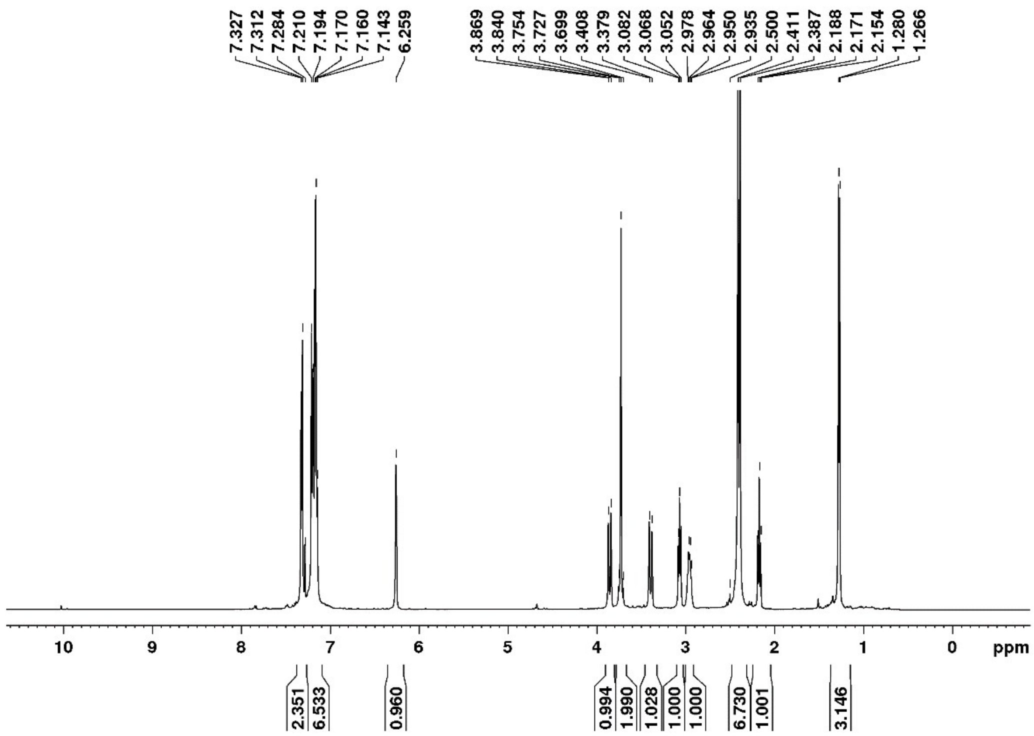

^{13}C-NMR spectrum of (*Z*)-3-methyl-1-(4-methylbenzyl)-4-(4-methylbenzylidene)pyrrolidine (**3i**)

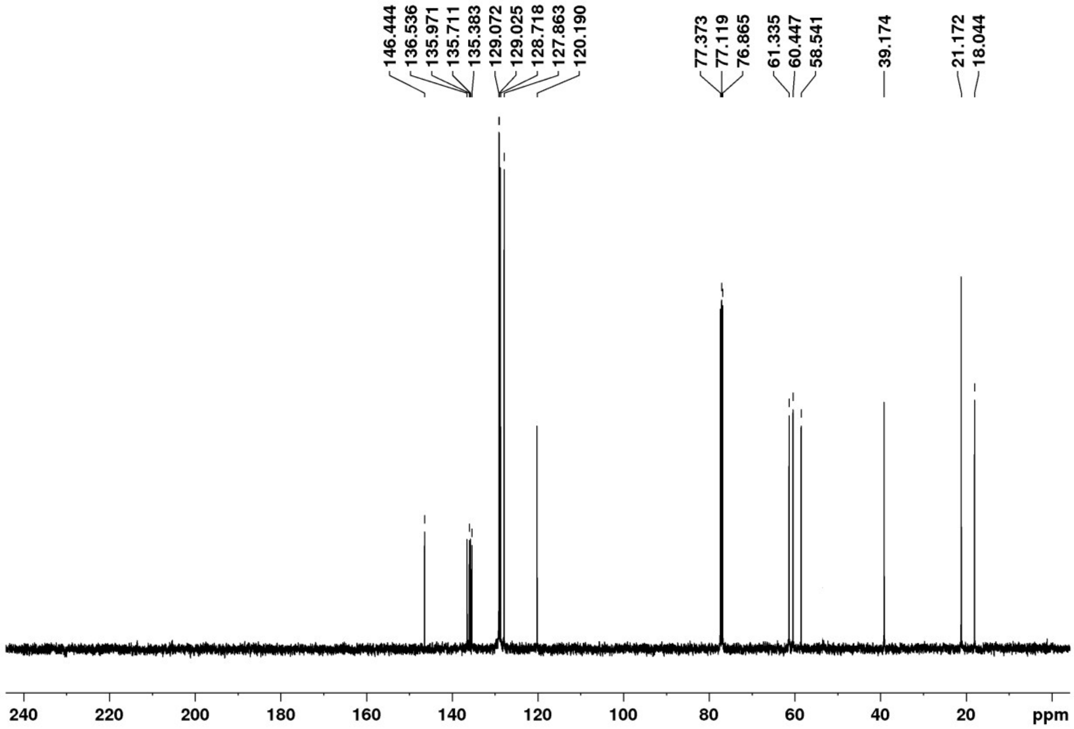

^{1}H-NMR spectrum of (*E*)-3-(iodo(trimethylsilyl)methylene)-4-(iodomethyl)-1-(4-methylbenzyl)pyrrolidin-2-one (**5h**)

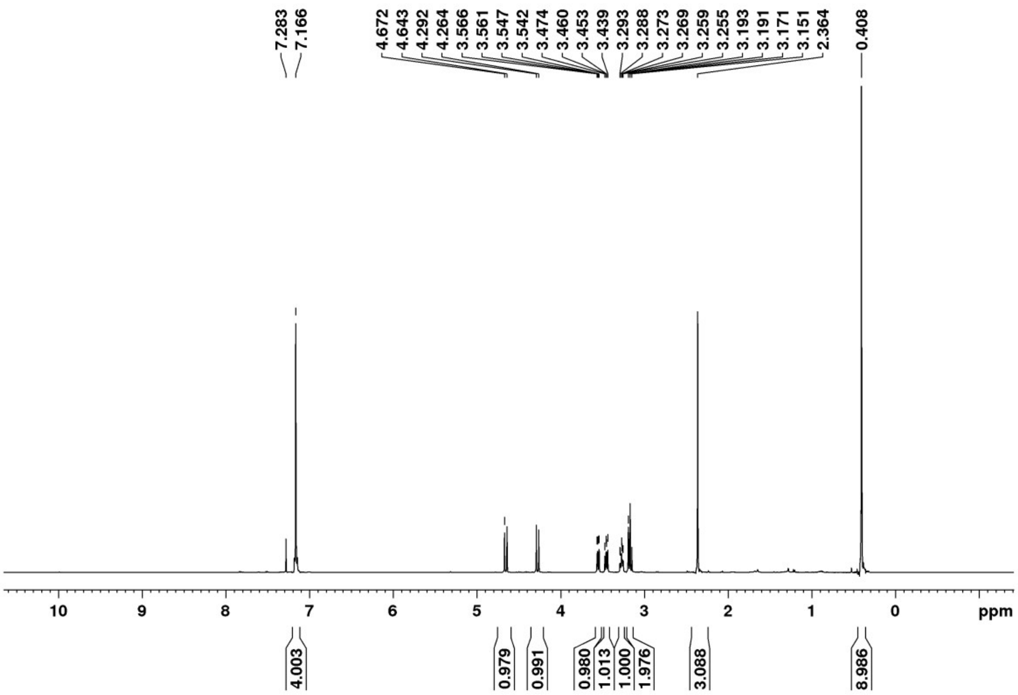

^{13}C-NMR spectrum of (*E*)-3-(iodo(trimethylsilyl)methylene)-4-(iodomethyl)-1-(4-methylbenzyl)pyrrolidin-2-one (**5h**)

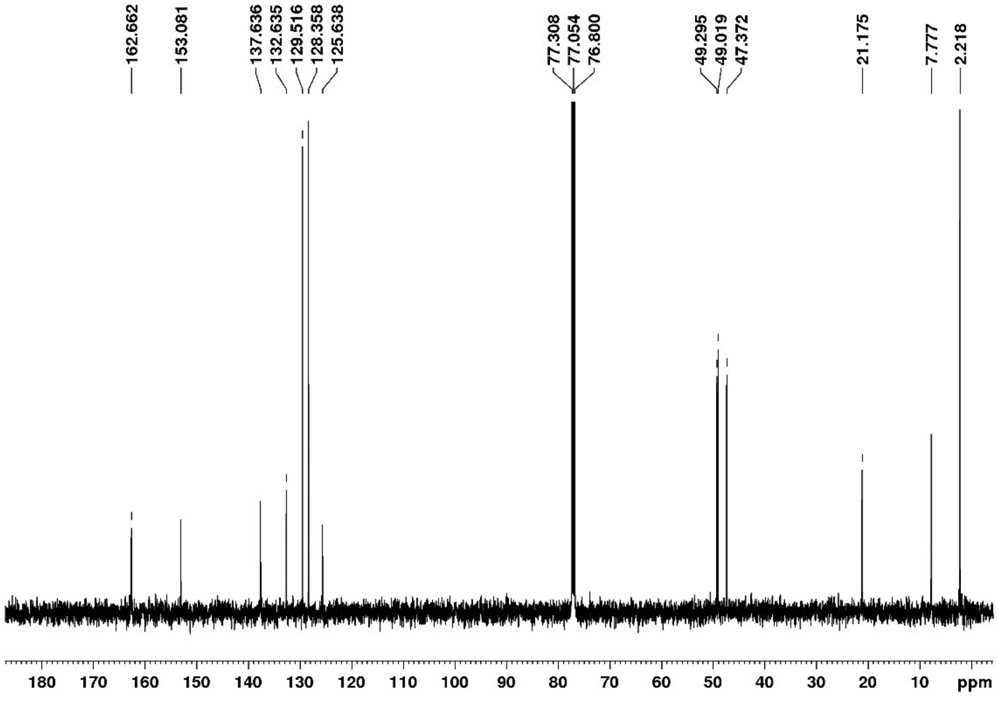

^{1}H-NMR spectrum of (*E*)-3-(iodo(trimethylsilyl)methylene)-4-(iodomethyl)-1-(4-methoxybenzyl)pyrrolidin-2-one (**5a**)

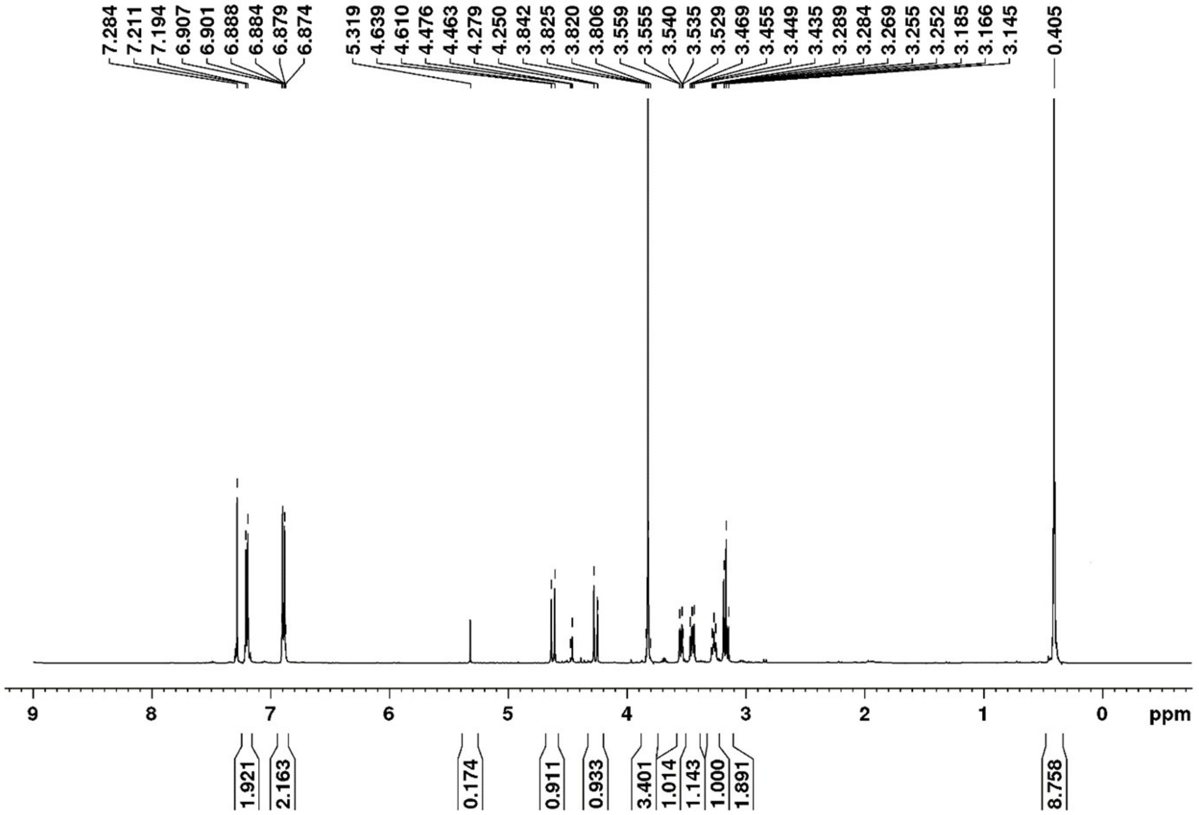

^{13}C-NMR spectrum of (*E*)-3-(iodo(trimethylsilyl)methylene)-4-(iodomethyl)-1-(4-methoxybenzyl)pyrrolidin-2-one (**5a**)

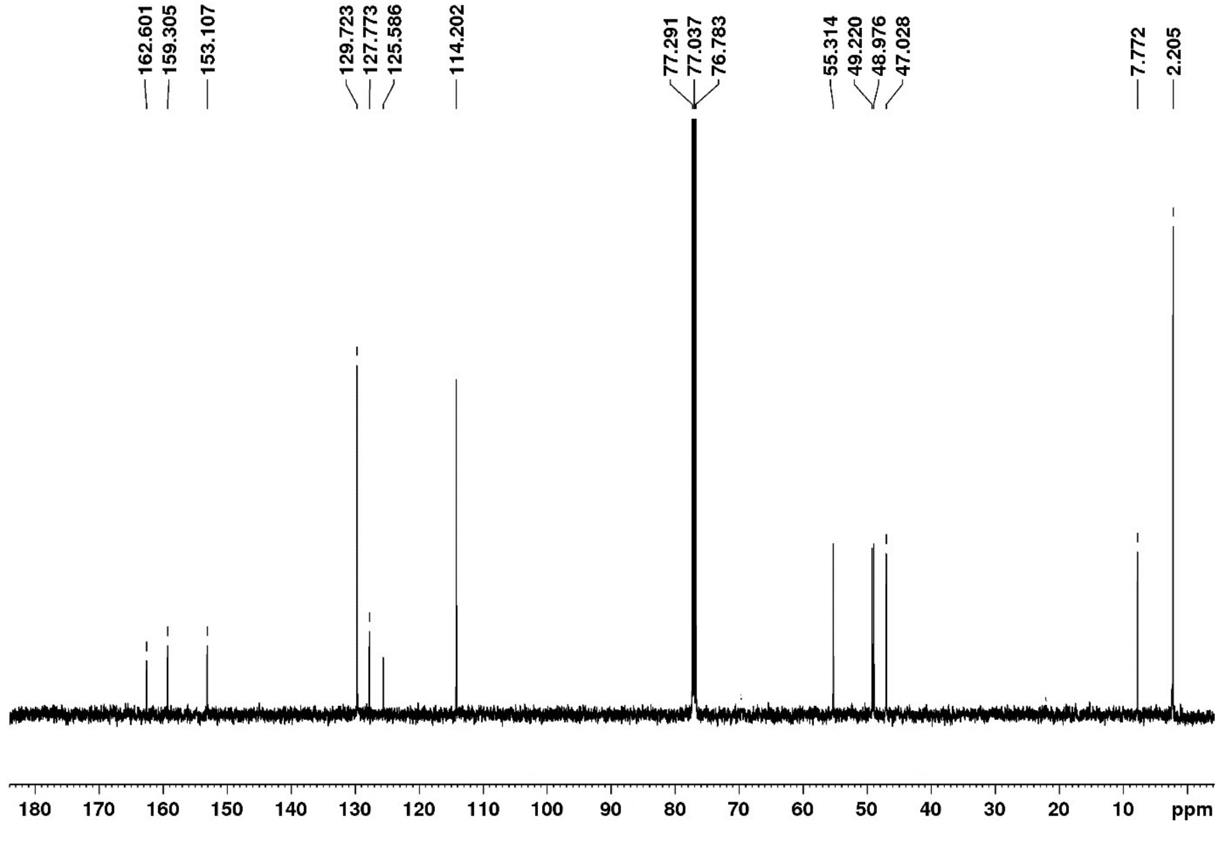

^{1}H-NMR spectrum of 1,4-bis(((*Z*)-3-methyl-4-((trimethylsilyl)methylene)pyrrolidin-1-yl)methyl)benzene (**7**)

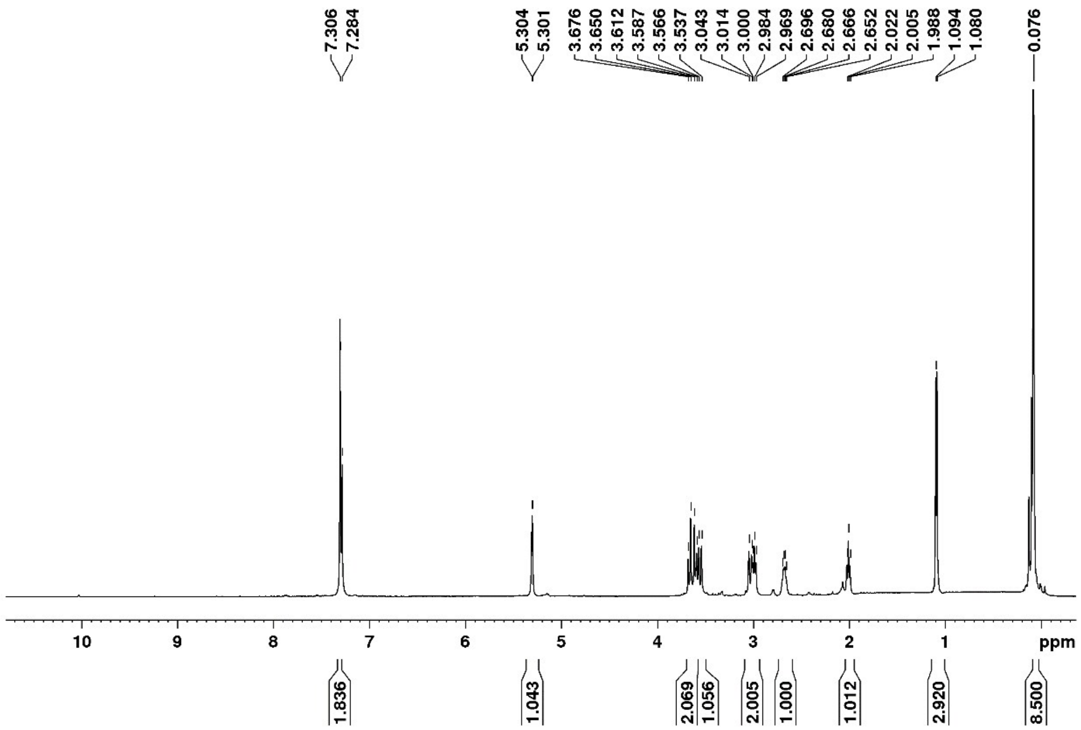

^{13}C-NMR spectrum of 1,4-bis(((*Z*)-3-methyl-4-((trimethylsilyl)methylene)pyrrolidin-1-yl)methyl)benzene (**7**)

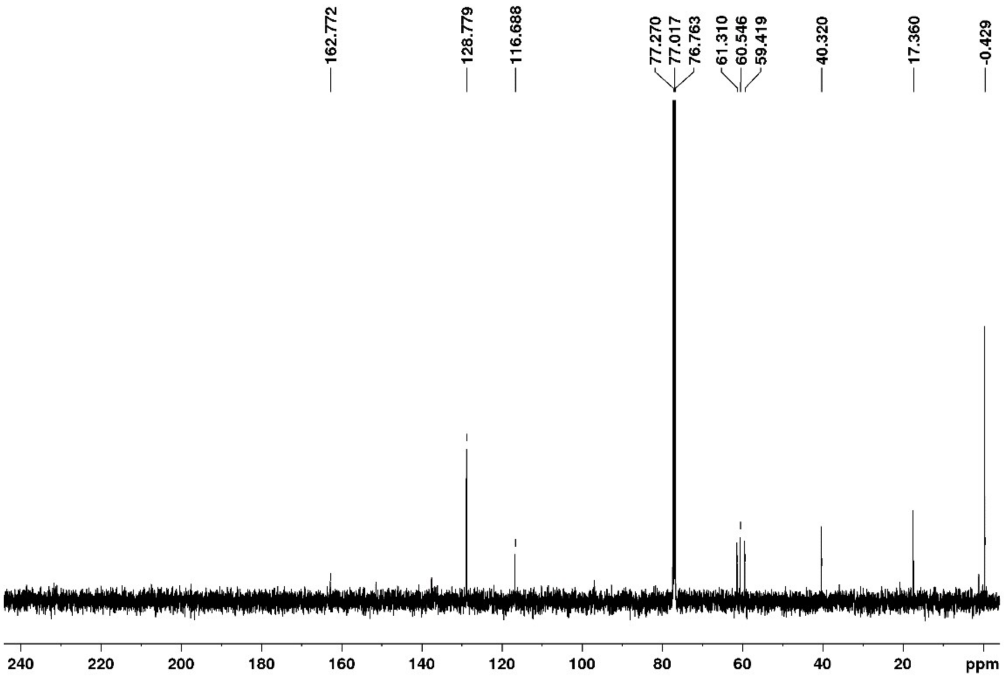

^{1}H-NMR spectrum of (4*Z*,4'*Z*)-4,4'-(octane-2,7-diylidene)bis(3-ethyl-1-(4-methylbenzyl)pyrrolidine) (**9**)

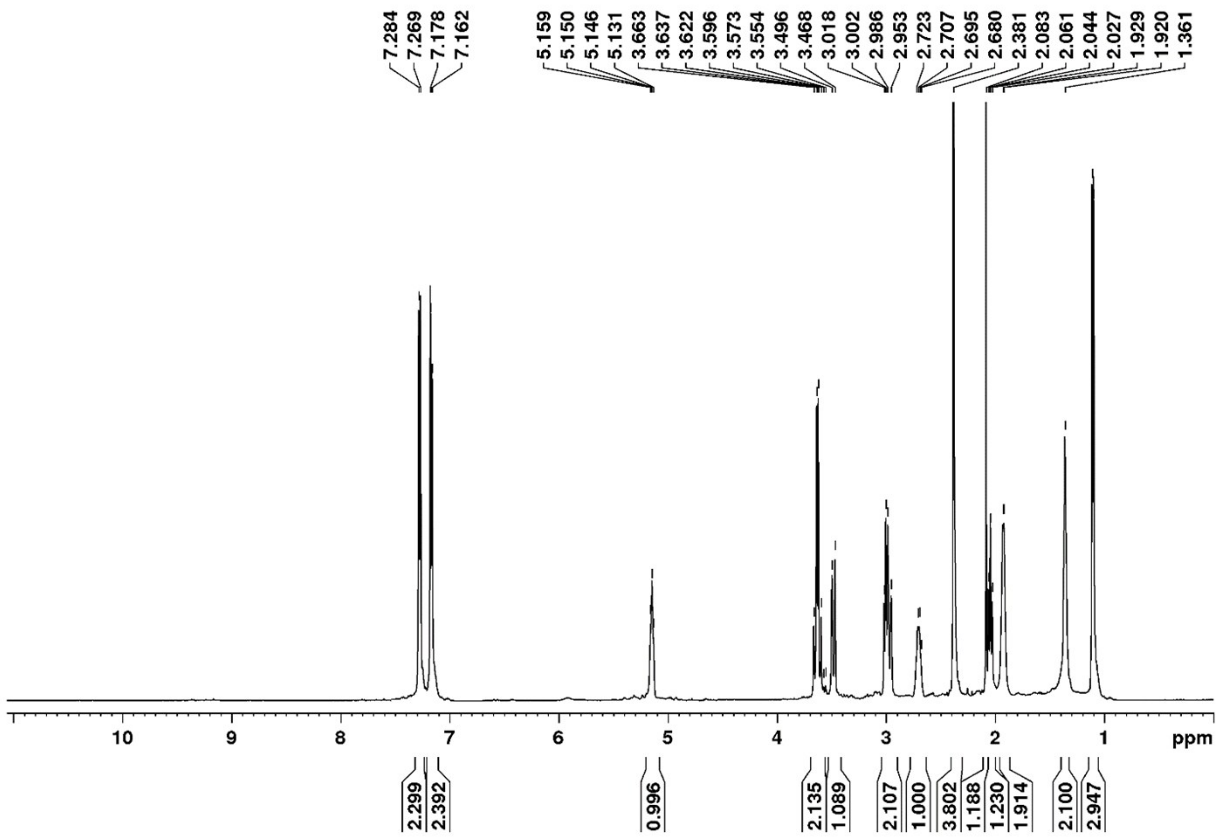

^{13}C-NMR spectrum of (4*Z*,4'*Z*)-4,4'-(octane-2,7-diylidene)bis(3-ethyl-1-(4-methylbenzyl)pyrrolidine) (**9**)

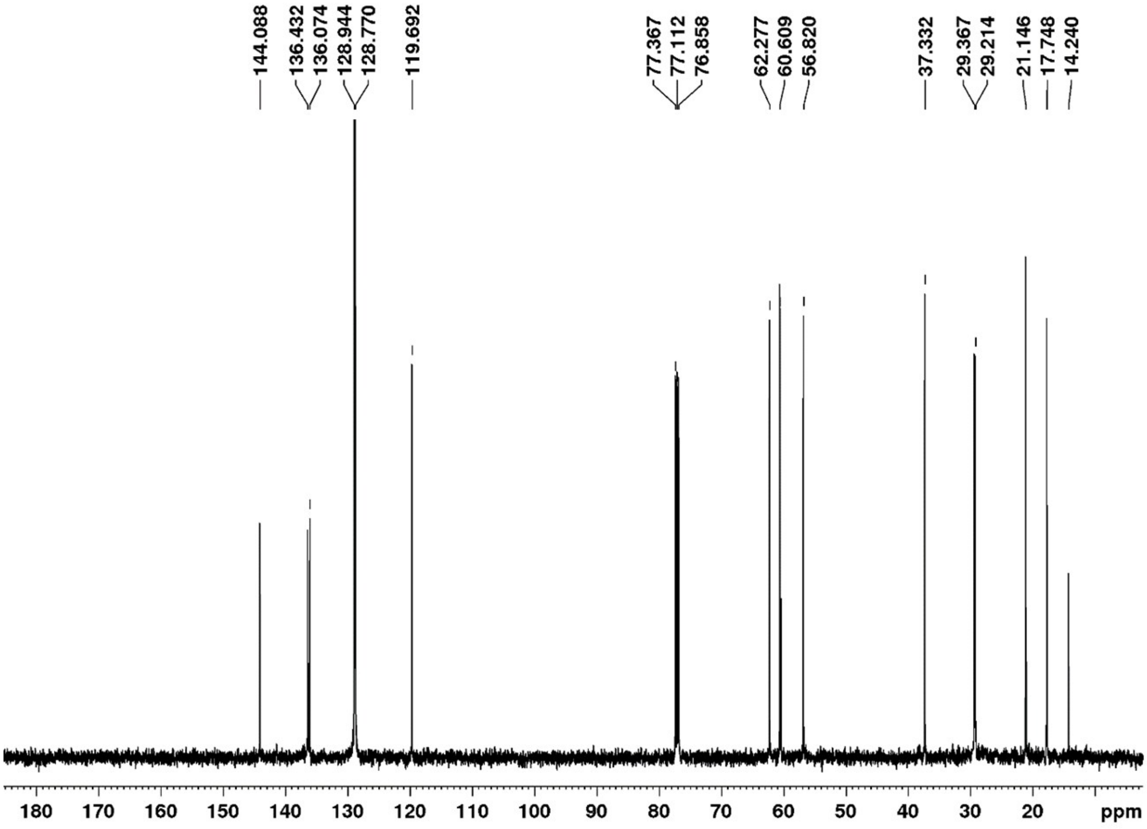

^{1}H-NMR spectrum of (*Z*)-*N*,*N*-dimethyl-2-(4-methyl-1-(4-methylbenzyl)pyrrolidin-3-ylidene)ethan-1-amine (**12a**)

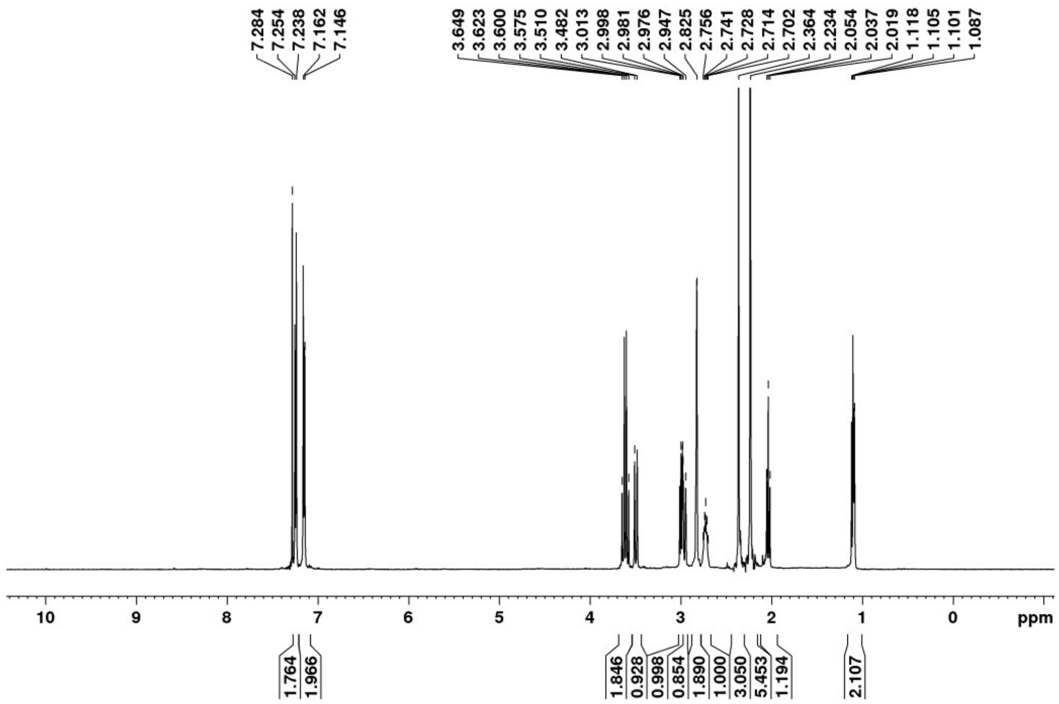

^{13}C-NMR spectrum of (*Z*)-*N*,*N*-dimethyl-2-(4-methyl-1-(4-methylbenzyl)pyrrolidin-3-ylidene)ethan-1-amine (**12a**)

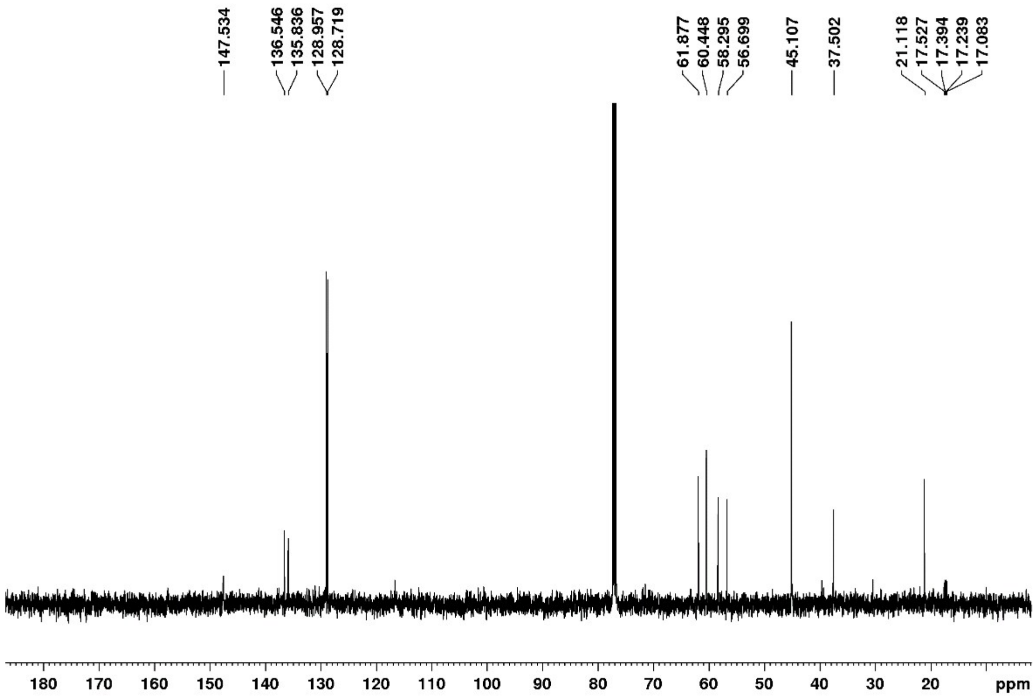

^{1}H-NMR spectrum of (*Z*)-1-(2-(1-(4-methoxybenzyl)-4-methylpyrrolidin-3-ylidene)ethyl)piperidine (**11c**)

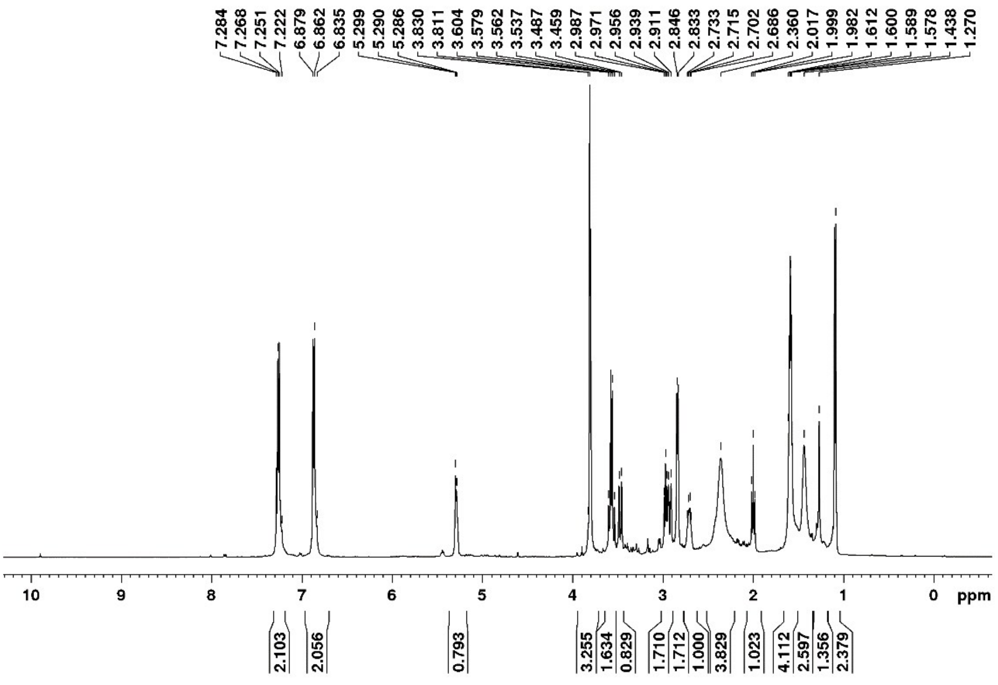

^{13}C-NMR spectrum of (*Z*)-1-(2-(1-(4-methoxybenzyl)-4-methylpyrrolidin-3-ylidene)ethyl)piperidine (**11c**)

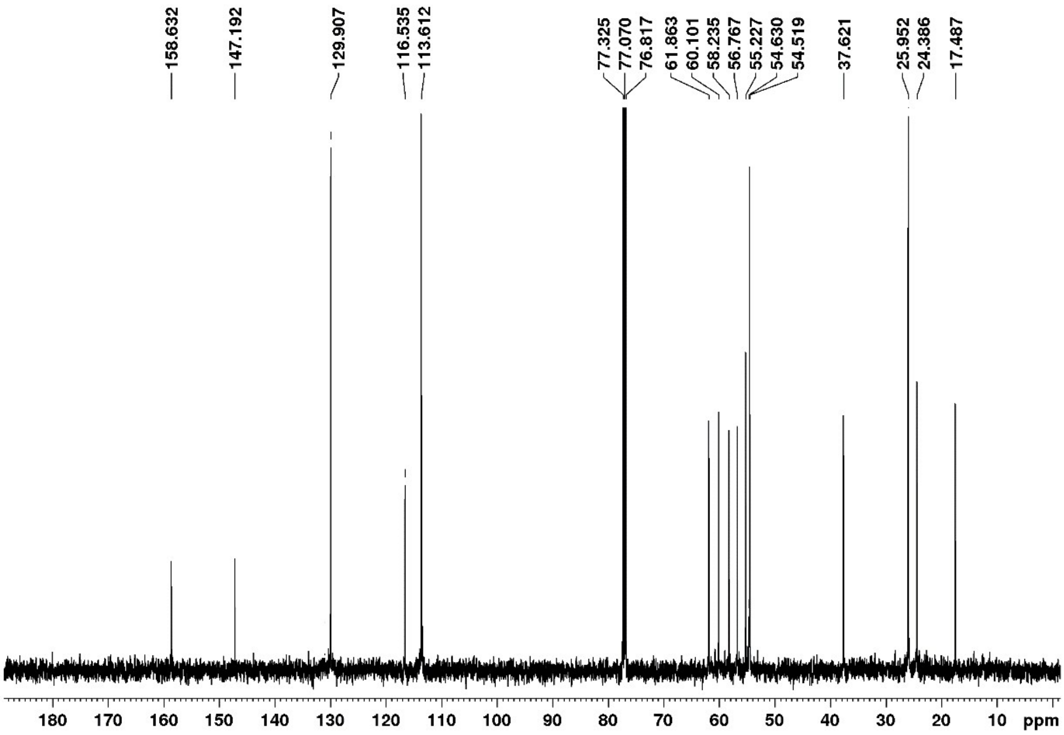

^{1}H-NMR spectrum of **(*Z*)-4-(2-(1-(4-methoxybenzyl)-4-methylpyrrolidin-3-ylidene)ethyl)morpholine (11b)**

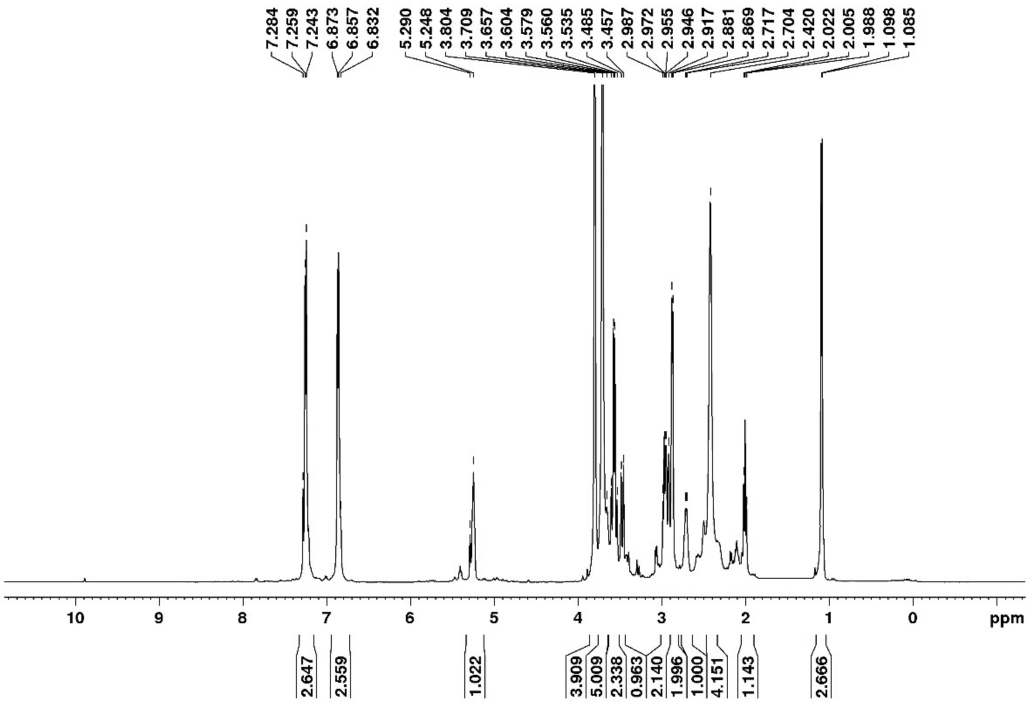

^{13}C-NMR spectrum of **(*Z*)-4-(2-(1-(4-methoxybenzyl)-4-methylpyrrolidin-3-ylidene)ethyl)morpholine (11b)**

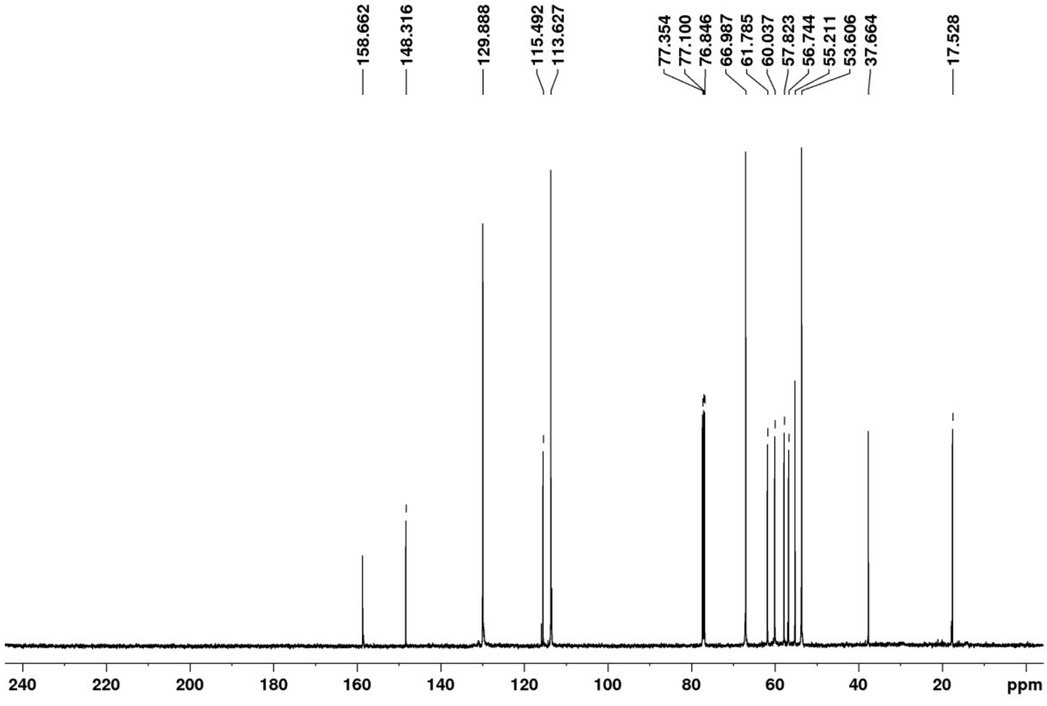

^{1}H-NMR spectrum of (*Z*)-3-(5-methoxypentylidene)-4-methyl-1-(4-methylbenzyl)pyrrolidine (**14a**)

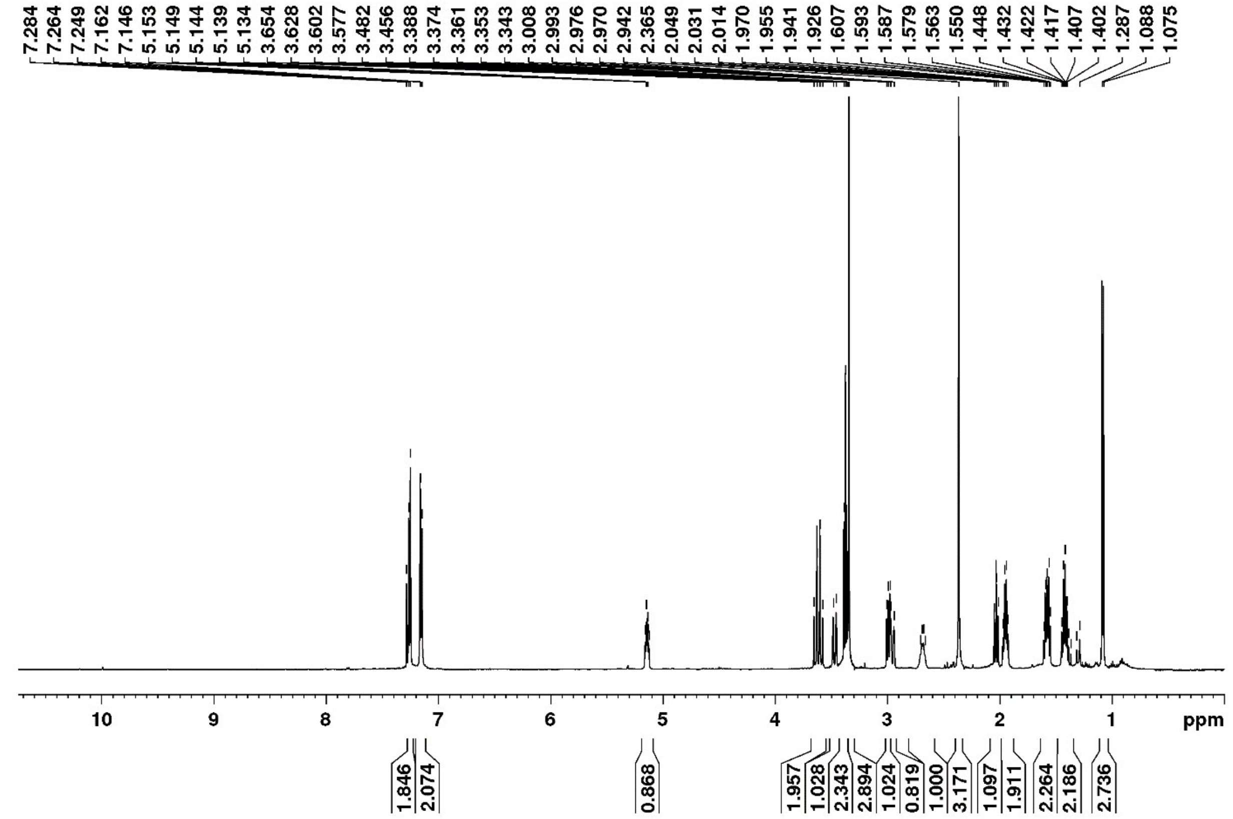

^{13}C-NMR spectrum of (*Z*)-3-(5-methoxypentylidene)-4-methyl-1-(4-methylbenzyl)pyrrolidine (**14a**)

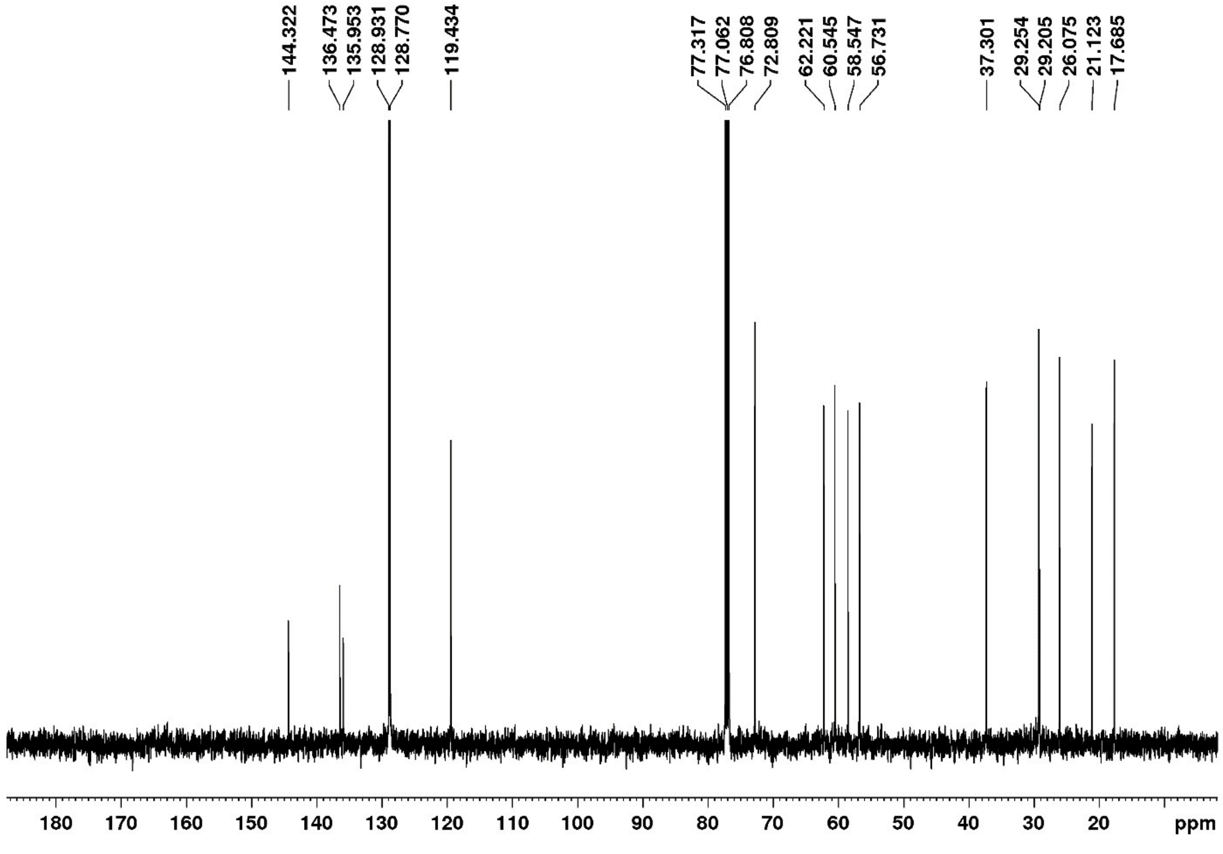

Supplement: RA-010-D0RA02677H-s001 [file RA-010-D0RA02677H-s001.pdf]
